# Enantioselective Benzylation and Allylation of α-Trifluoromethoxy Indanones under Phase-Transfer Catalysis

**DOI:** 10.3390/molecules24152774

**Published:** 2019-07-30

**Authors:** Yumeng Liang, Mayaka Maeno, Zhengyu Zhao, Norio Shibata

**Affiliations:** 1 Department of Nanopharmaceutical Sciences, Department of Life Science and Applied Chemistry, Nagoya Institute of Technology Gokiso, Showa-ku, Nagoya 466-8555, Japan; 2 Institute of Advanced Fluorine-Containing Materials, Zhejiang Normal University, 688 Yingbin Avenue, Jinhua 321004, China

**Keywords:** trifluoromethoxy, fluorine, enantioselective, phase-transfer catalyst, organo-catalysis

## Abstract

The organo-catalyzed enantioselective benzylation reaction of α-trifluoromethoxy indanones afforded α-benzyl-α-trifluoromethoxy indanones with a tetrasubstituted stereogenic carbon center in excellent yield with moderate enantioselectivity (up to 57% ee). Cinchona alkaloid-based chiral phase transfer catalysts were found to be effective for this transformation, and both enantiomers of α-benzyl-α-trifluoromethoxy indanones were accessed, depended on the use of cinchonidine and cinchonine-derived catalyst. The method was extended to the enantioselective allylation reaction of α-trifluoromethoxy indanones to give the allylation products in moderate yield with good enantioselectivity (up to 76% ee).

## 1. Introduction

The role of fluorine in medicinal chemistry is expanding rapidly after it was discovered that the introduction of fluorine into an organic molecule could productively influence its physical and chemical properties [1,2,3,4,5,6,7,8,9,10,11,12,13,14]. In particular, the trifluoromethoxy (OCF_3_) functional group has received extensive attention in recent years in the fields of pharmaceuticals and agrochemicals, owing to its unique three-dimensional electronic properties, suitable lipophilic properties, and good metabolic stability [15,16,17,18,19,20]. In fact, the OCF_3_ group is present in more than 1393 biologically active organic compounds according to a check of the PubChem database in June 2019 [21,22,23]. Compared to trifluoromethyl (CF_3_; πx = 0.88), methyl (CH_3_; πx = 0.52) and methoxy (OCH_3_) groups (πx = −0.02), the OCF_3_ group has the highest lipophilicity value (πx = 1.04) [24,25,26,27,28,29] resulting in the potential improvement of metabolic profiles, including permeability and absorption, when it is introduced into the appropriate position of parent molecules.

**Scheme 1:**
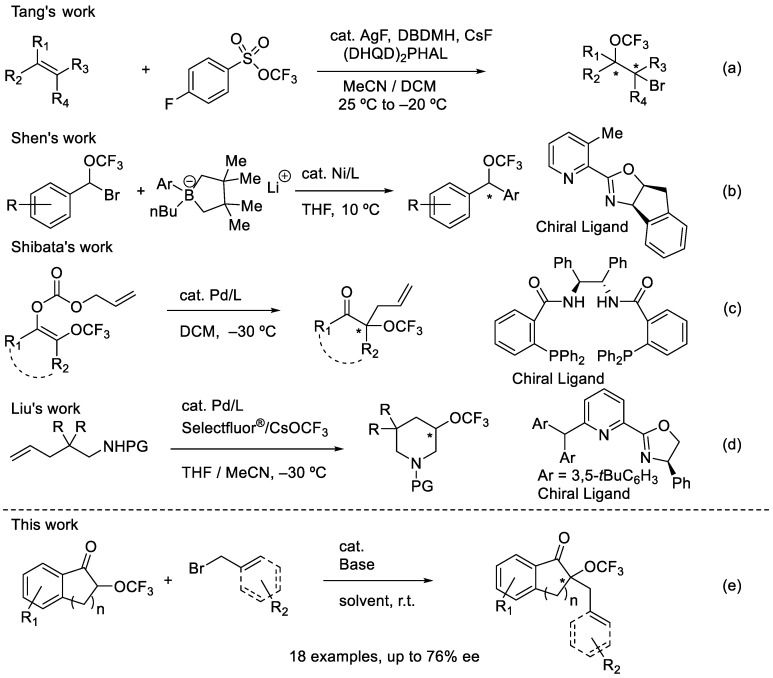
Enantioselective synthesis of trifluoromethoxy-containing compounds.

In contrast to the requirement of OCF_3_-containing drug candidates in medicinal chemistry, the synthesis of OCF_3_-containing organic compounds is relatively problematic. The OCF_3_ unit is traditionally synthesized from its chlorinated precursor, the trichloromethoxy (OCCl_3_) moiety, by a chlorine/fluorine exchange reaction under harsh reaction conditions [30,31,32,33,34,35]. The OCF_3_ anion is unstable and decomposes rapidly into difluorophosgene (O=CF_2_) and a fluoride anion (F^−^), which can make nucleophilic trifluoromethylation difficult [36]. The electrophilic trifluoromethylation of hydroxyl compounds is another strategy, but the method is somewhat limited. While the synthesis of OCF_3_-containing organic compounds has improved dramatically over the last five years [37,38,39,40,41,42], a method that can be used to construct a chiral “C*–OCF_3_” unit is still extremely scarce. This should be one of the reasons of no pharmaceuticals with chiral, aliphatic “C*–OCF_3_” unit reported. In 2017, Tang and co-workers reported the enantioselective bromo-trifluoromethoxylation of olefins by trifluoromethyl arylsulfonate (TFMS) under silver catalysis in the presence of 1,3-dibromo-5,5-dimethylhydantoin (DBDMH; Scheme 1a) [43]. Later, Shen and co-workers reported a method to construct chiral trifluoromethoxyl compounds by the Ni-catalyzed enantioselective Suzuki–Miyaura coupling of secondary benzyl bromides in good to high enantioselectivity (Scheme 1b) [44]. We developed a strategy for the synthesis of chiral, non-racemic α-OCF_3_-ketones with a tetrasubstituted carbon center via a Pd-catalyzed enantioselective Tsuji-allylation reaction with high enantioselectivity (Scheme 1c) [45,46]. Very recently, Liu and co-workers reported the Pd-catalyzed enantioselective intramolecular trifluoromethoxylation reaction of alkenes using CsOCF_3_ to furnish OCF_3_-compounds with a chiral stereogenic center (Scheme 1d) [47]. While these methods have broad substrate scopes with high enantioselectivity, all the methods require transition metal catalysts. Herein, we report the first example of constructing molecules with an OCF_3_ chiral center under non-metallic, organocatalytic conditions. The α-OCF_3_ indanones react with benzyl bromides in the presence of a cinchona alkaloid-derived chiral phase-transfer catalyst (PTC) to afford enantioenriched α-benzyl-α-OCF_3_ indanones in high yield with up to 57% ee. Access to both (*R*)- and (*S*)-enantiomers of α-benzyl-α-OCF_3_ indanones can be controlled by the catalysts. The method was expanded to the enantioselective allylation reaction with allyl bromide to provide α-allyl-α-OCF_3_ indanones with up to 76% ee (Scheme 1e).

## 2. Results and Discussion

The enantioselective benzylation of α-OCF_3_-substituted indanone **1a** with benzyl bromide (**2a**) was first examined (Table 1). The screening of representative cinchonine-derived PTCs, CN-1–CN-8 (entries 1–8) in toluene revealed that *N*-[4-(trifluoromethyl)benzyl]cinchoninium bromide CN-4 exhibited potential performance with 88% yield with 26% ee of (+)-**3aa**. We next examined solvents (*n*-hexane, dichloromethane (DCM), tetrahydrofuran (THF), diethyl ether (Et_2_O) and *N,N*-dimethylformamide (DMF)), but no satisfying consequences were observed (entries 9–13). Bases were next examined (potassium carbonate (K_2_CO_3_), sodium hydroxide (NaOH), cesium hydroxide monohydrate (CsOH·H_2_O), lithium hydroxide (LiOH), sodium hydride (NaH), potassium acetate (KOAc), dipotassium phosphate (K_2_HPO_4_) potassium *tert*-butoxide (KO*t*Bu) and potassium methoxide (KOMe; entries 14–22). A 50% KOH aqueous solution and the addition 2 equiv water with KOH were also tested (entries 23–24), the results showed H_2_O could not increase yield or enantioselectivity. Among these, CsOH·H_2_O exhibited the best performance (83% yield) with 43% ee of (+)-**3aa**. Additional solvent screening using CsOH·H_2_O (benzotrifluoride (PhCF_3_), toluene: CHCl_3_ = 7:3; entries 25–26) revealed no improvement in the results. Additional catalyst CN-9 was used, racemic product was obtained (entry 27). This result indicated that a free-OH group on the phase-transfer catalyst should play an important role for the induction of enantioselectivity, such as hydrogen bonding. The concentration of the reaction (0.1 M to 0.02 M) and the temperature affected selectivity (entries 28–29), and the best results obtained were 80% yield with 54% ee (entry 30). The product with an opposite configuration, (−)-3aa was obtained in 75% yield with 50% ee using CD-4 (entry 32). More optimization results using other PTC were shown in the supporting information (SI; Table S1, in SI). 

**Scheme 2:**
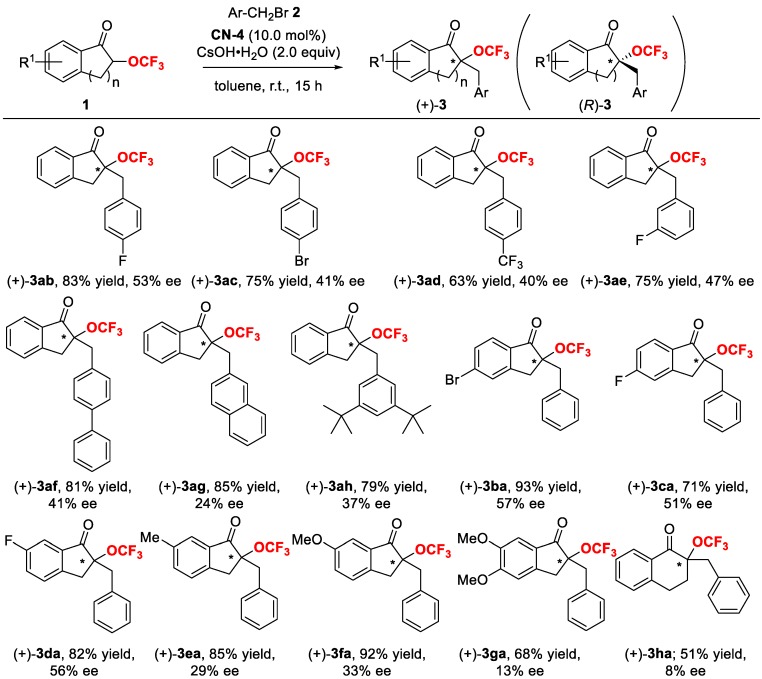
The enantioselective benzylation of α-trifluoromethoxy ketones **1** under **CN-4** catalysis.

With the optimal reaction conditions in hand (Table 1, entry 30), we explored the substrate scope of this enantioselective catalytic benzylation of α-OCF_3_ indanones 1 (Scheme 2). With a variety of benzyl bromides **2b**–**2g** under the optimal conditions, the desired OCF_3_ indanones (+)-**3ab**–**3ah** were obtained in good to high yield (63%–85%) with moderate ee (24%–53%). Indanones with an electron-withdrawing group on the aromatic ring (**1b**–**1d**) gave the desired products (+)-**3ba**–**3da** in high to excellent yield (73%–93%) with good ee (51%–57%). On the other hand, indanones with an electron-donating group (**1e**–**1g**) furnished products (+)-3**ea**–3**ga** in high to excellent yield, but the ee decreased (13%–33% ee). The method was unsuitable for the benzylation of α-OCF_3_ tetralone 1h under the same conditions; the corresponding product **3ha** was detected in 51% yield with 8% ee. 

**Scheme 3:**
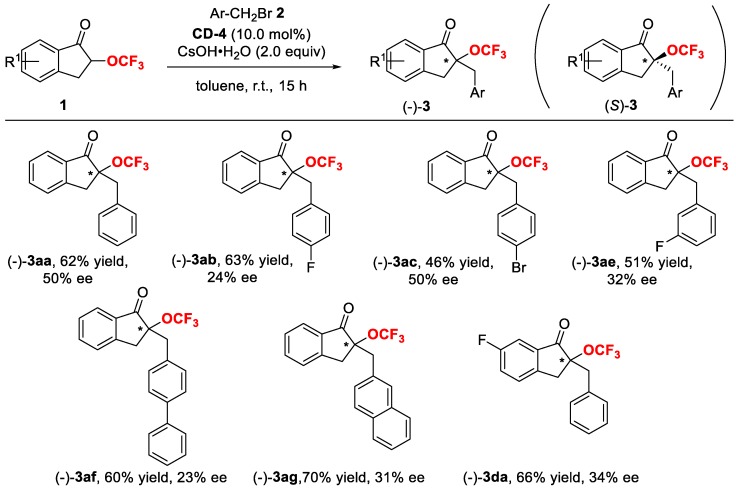
The enantioselective benzylation of α-trifluoromethoxy indanones **1** under **CD-4** catalysis.

**Scheme 4:**
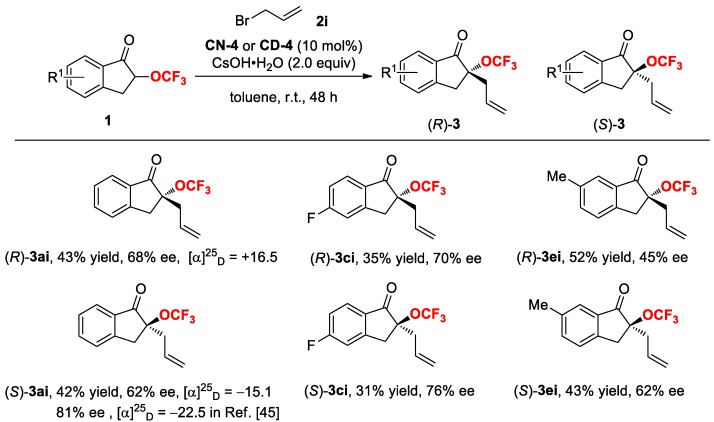
The enantioselective allylation of α-trifluoromethoxy indanones **1** under **CN-4** or **CD-4** catalysis. For (*R*)-**3**, **CN-4** was used. For (*S*)-**3**, **CD-4** was used.

The substrate scope of the enantioselective benzylation of α-OCF_3_ indanones **1** using a catalyst, **CN-4**, under the same reaction conditions furnished (−)-**3** ((*S*)-**3**) with an opposite configuration in similar yield and up to 50% ee (Scheme 3). The absolute stereochemistry of the (+)-**3** was temporality assigned to be (*R*) based on the results for the enantioselective allylation of **1** with allyl bromide (**2i**) as discussed below (see the later part of this paper, Scheme 4). 

It should be noted that the method could be applied for the enantioselective allylation of α-OCF_3_ indanones **1** with allyl bromide (**2i**) under **CN-4** or **CD-4** catalysis. The desired (+)- and (−)-α-allyl-α-OCF_3_ indanones **3ai** were obtained in moderate yield with up to 70% ee and 76% ee, respectively (Scheme 4). The absolute configurations of **3ai** were determined to be the (*R*)-configuration for (+)-**3ai** and the (*S*)-configuration for (−)-**3ai** by comparing to the optical rotation of reported (*S*)-**3ai** (${\left[\alpha \right]}_{D}^{25}$ = −22.5) [45]. The higher enantioselectivity with allylic substrates is most likely due to the less steric hindrance than benzyl bromides.

## 3. Materials and Methods

### 3.1. General Information

All reagents were used as received from commercial sources, unless specified otherwise. All reactions were performed in oven-dried glassware under a positive pressure of nitrogen. Solvents were transferred via syringe and were introduced into the reaction vessels though a rubber septum. All reactions were monitored by thin-layer chromatography (TLC) carried out on 0.25 mm Merck silica-gel (60-F254) (Kenilworth, NJ, USA). The TLC plates were visualized with UV light (254 nm) (Tokyo, Japan) and *p*-Anisaldehyde in ethanol/heat. Column chromatography was carried out on a column packed with silica-gel 60N spherical neutral size 63–210 μm or 40–63 μm. The ^1^H-NMR (300 MHz), ^19^F-NMR (282 MHz) and ^13^C-NMR (126 MHz) spectra for solution in CDCl_3_ were recorded on a Bruker Avance 500 (Karlsruhe, Germany), Varian Mercury 300 (Palo Alto, CA, USA). Chemical shifts (δ) are expressed in ppm downfield from tetramethylsilane (δH = 0.00 ppm) or tetramethylsilane (δC = 0.00 ppm) or hexafluorobenzene (δF = −162.20 ppm). Optical rotations were measured with a Horiba SEPA-300 operating at 589 nm (Kyoto, Japan). Mass spectra were recorded on an LCMS-2020EV (ESI-MS) system (Shimadzu Corporation, Kyoto, Japan). High resolution mass spectrometry (HRMS) was recorded on a Waters Synapt G2 HDMS (ESI-MS) (Milford, MA, USA). The wave numbers (*ν*) of recorded IR-signals are quoted in cm^−1^ on a JASCO FT/IR-4100 spectrometer (Tokyo, Japan). HPLC analyses were performed on a JASCOLC-2000 Plus series (Tokyo, Japan) using 4.6 × 250 mm CHIRALCEL^®^ series or CHIRALPAK series (Tokyo, Japan). The melting point was recorded on a BUCHI M-565 (Flawil, Switzerland). All solvents were dried and distilled before use. The ^1^H, ^13^C and ^19^F-NMR spectra of compounds **3** and HPLC data of compounds **3** are available in the Supplementary Material.

### 3.2. Preparation of α-OCF_3_-Substituted Indanones (General Procedure)

All the substrates, α-OCF_3_-indanones **1**, were prepared by following a reported procedure [45].

General Procedure: 

A mixture of the indanone (1.0 equiv) and KOH (3.0 equiv) in MeOH (0.4 M) was stirred for 15 min at 0 °C, and PhI(OAc)_2_ (1.1 equiv) was added in 4–5 portions during 5 min. The mixture was stirred at the same temperature for 1 h, then warmed to room temperature and stirred overnight. The mixture was concentrated, dissolved in Et_2_O, washed with NaHCO_3_ aq., dried over Na_2_SO_4_ and concentrated, then purified by silica-gel column chromatography. The pure product was then dissolved in EtOH (0.3 M), and 3N HCl aq. (1.0 M) was added. After stirring for 0.5 h at room temperature, the resulting mixture was extracted with Et_2_O, and the combined organic layer was washed with sat. NaHCO_3_ aq. and brine, then dried over Na_2_SO_4_. The residue can be used without further purification for the next reaction.

A flask was charged with hydroxyketone, AgOTf (3.0 equiv), KF (4.0 equiv) and Selectfluor^®^ (1.5 equiv) in a nitrogen-filled glovebox. Then ethyl acetate (0.2 M), 2-fluoropyridine (3.0 equiv) and Me_3_SiCF_3_ (3.0 equiv) were added successively under an Ar atmosphere. The resulting mixture was stirred overnight at room temperature. The reaction mixture was filtered through a pad of silica-gel and concentrated. The residue was purified by flash silica-gel column chromatography.

*2-(Trifluoromethoxy)-2,3-dihydro-1H-inden-1-one* (**1a**). The reaction was run according to the general procedure, and the product was consistent with previously reported characterization data [45]. ^1^H-NMR (300 MHz, CDCl_3_) δ: 7.82 (d, *J* = 6.9 Hz, 1H), 7.71–7.66 (m, 1H), 7.49–7.42 (m, 2H), 4.98–4.91 (m, 1H), 3.68 (dd, *J* = 17.0, 7.9 Hz, 1H) and 3.26 (dd, *J* = 17.0, 5.0 Hz, 1H) ppm. ^19^F-NMR (282 MHz, CDCl_3_) δ: −59.66 (s, 3F) ppm.

*5-Bromo-2-(trifluoromethoxy)-2,3-dihydro-1H-inden-1-one* (**1b**). The reaction was run according to the general procedure, and the product was consistent with previously reported characterization data [45]. ^1^H-NMR (300 MHz, CDCl_3_) δ: 7.70–7.58 (m, 3H), 4.94–4.90 (m, 1H), 3.66 (dd, *J* = 17.2, 7.9 Hz, 1H) and 3.25 (dd, *J* = 17.2, 4.9 Hz, 1H) ppm. ^19^F-NMR (282 MHz, CDCl_3_) δ: −59.82 (s, 3F) ppm.

*5-Fluoro-2-(trifluoromethoxy)-2,3-dihydro-1H-inden-1-one* (**1c**). The reaction was run according to the general procedure, and the product was consistent with previously reported characterization data [45]. ^1^H-NMR (300 MHz, CDCl_3_) δ: 7.85 (dd, *J* = 9.2, 5.4 Hz, 1H), 7.19–7.13 (m, 2H), 4.94 (dd, *J* = 7.9, 4.8 Hz, 1H), 3.67 (dd, *J* = 17.3, 7.9 Hz, 1H) and 3.26 (dd, *J* = 17.3, 4.9 Hz, 1H) ppm. ^19^F-NMR (282 MHz, CDCl_3_) δ: −59.81 (s, 3F) and −99.41 (q, *J* = 8.1 Hz, 1F) ppm.

*6-Fluoro-2-(trifluoromethoxy)-2,3-dihydro-1H-inden-1-one* (**1d**). The reaction was run according to the general procedure. ^1^H-NMR (300 MHz, CDCl_3_) δ: 7.48–7.37 (m, 3H), 4.96 (dd, *J* = 8.0, 4.8 Hz, 1H), 3.66 (dd, *J* = 16.8, 7.9 Hz, 1H), 3.22 (dd, *J* = 16.8, 5.0 Hz, 1H) ppm. ^19^F-NMR (282 MHz, CDCl_3_) δ: −59.85 (s, 3F), −111.30–−112.37 (m, 1F) ppm. ^13^C-NMR (126 MHz, CDCl_3_) δ: 196.73 (d, *J* = 3.0 Hz), 162.72 (d, *J* = 250.2 Hz), 145.06 (d, *J* = 2.4 Hz), 135.29 (d, *J* = 7.7 Hz), 128.34 (d, *J* = 7.8 Hz), 124.25 (d, *J* = 23.8 Hz), 121.88 (q, *J* = 256.9 Hz), 110.70 (d, *J* = 22.3 Hz), 76.85 (q, *J* = 2.5 Hz) and 33.19 ppm. MS (ESI): *m/z* 233 [M − H]^−^. m.p.: 93.4–95.1 °C.

*6-Methyl-2-(trifluoromethoxy)-2,3-dihydro-1H-inden-1-one* (**1e**). The reaction was run according to the general procedure, and the product was consistent with previously reported characterization data. [45] ^1^H-NMR (300 MHz, CDCl_3_) δ: 7.62 (s, 1H), 7.50 (d, *J* = 7.5, 1H), 7.35 (d, *J* = 7.9, 1H), 4.92 (dd, *J* = 7.9 Hz, 1H), 3.67 (dd, *J* = 17.3, 7.9 Hz, 1H) and 3.26 (dd, *J* = 17.3, 4.9 Hz, 1H) ppm. ^19^F-NMR (282 MHz, CDCl_3_) δ: −59.72 (s, 3F) ppm.

*6-Methoxy-2-(trifluoromethoxy)-2,3-dihydro-1H-inden-1-one* (**1f**). The reaction was run according to the general procedure, and the product was consistent with previously reported characterization data. [45] ^1^H-NMR (300 MHz, CDCl_3_) δ: 7.37 (d, *J* = 8.3, 1H), 7.29–7.23 (m, 2H), 4.97–4.92 (m, 1H), 3.86 (s, 3H), 3.61 (dd, *J* = 16.6, 7.8 Hz, 1H) and 3.18 (dd, *J* = 16.6, 4.7 Hz, 1H) ppm. ^19^F-NMR (282 MHz, CDCl_3_) δ: −59.73 (s, 3F) ppm.

*5,6-Dimethoxy-2-(trifluoromethoxy)-2,3-dihydro-1H-inden-1-one* (**1g**). The reaction was run according to the general procedure. ^1^H-NMR (300 MHz, CDCl_3_) δ: 7.24 (d, *J* = 14.6, 1H), 4.90 (dd, *J* = 7.6, 4.3 Hz, 1H), 3.99 (s, 3H), 3.92 (s, 3H), 3.58 (dd, *J* = 16.8, 7.6 Hz, 1H) and 3.17 (dd, *J* = 16.8, 4.3 Hz, 1H) ppm. ^19^F-NMR (282 MHz, CDCl_3_) δ: −59.61 (s, 3F) ppm. ^13^C-NMR (126 MHz, CDCl_3_) δ: 195.90, 156.89, 150.23, 145.38, 126.50, 121.97 (q, *J* = 256.5 Hz), 107.30, 104.91, 76.51 (q, *J* = 2.3 Hz), 56.44, 56.22 and 33.54 ppm. MS (ESI): *m/z* 277 [M + H]^+^. m.p.: 136.9–138.0 °C.

*2-(Trifluoromethoxy)-3,4-dihydronaphthalen-1(2H)-one* (**1h**). The reaction was run according to the general procedure, and the product was consistent with previously reported characterization data. [45] ^1^H-NMR (300 MHz, CDCl_3_) δ: 8.07 (d, *J* = 7.9, 1H), 7.54 (t, *J* = 7.4, 1H), 7.37 (t, *J* = 7.7, 1H), 4.85 (dd, *J* = 12.1, 4.8 Hz, 1H), 3.16 (dd, *J* = 8.1, 4.4 Hz, 2H) and 2.57–2.36 (m, 2H) ppm. ^19^F-NMR (282 MHz, CDCl_3_) δ: −59.05 (s, 3F) ppm.

### 3.3. Representative Procedure for the Enantioselective Catalytic Phase Transfer Benzylation

A flask was charged with α-OCF_3_-indanone **1** (0.10 mmol, 1.0 equiv), CsOH·H_2_O (0.20 mmol, 2.0 equiv) and cat. **4** (0.010 mmol, 10.0 mol%) in a nitrogen-filled glovebox. Then anhydrous toluene (5.0 mL, 0.02 M) and **2** (0.15 mmol, 1.5 equiv) was added under an Ar atmosphere. The resulting mixture was stirred overnight or 48 h at room temperature. After that, the solvent was removed under reduced pressure and the residue was purified by flash silica-gel column chromatography.

*(+)-2-Benzyl-2-(trifluoromethoxy)-2,3-dihydro-1H-inden-1-one* ((+)-**3aa**). The reaction was run according to the general procedure. Eluent (*n*-hexane/ethyl acetate = 15/1). Slightly yellow oil; 24.5 mg; 80% yield. The enantiomeric excess (54% ee) was determined by chiral HPLC using CHIRALCEL^®^ OJ-H column (*n*-hexane/isopropanol = 99.0/1.0, flow rate 1.0 mL/min, λ = 254 nm) t (major) = 16.525 min, t (minor) = 11.367 min). ${\left[\alpha \right]}_{D}^{25}$ = +37.7 (CH_2_Cl_2_, *c* = 0.62).^1^H-NMR (300 MHz, CDCl_3_) δ: 7.78 (d, *J* = 7.7 Hz, 1H), 7.59 (t, *J* = 7.4 Hz, 1H), 7.40–7.32 (m, 2H), 7.22–7.17 (m, 5H), 3.43 (s, 2H), 3.31 (d, *J* = 13.8 Hz, 1H) and 3.01 (d, *J* = 13.8 Hz, 1H) ppm. ^13^C-NMR (126 MHz, CDCl_3_) δ: 200.07, 149.53, 136.16, 133.82, 133.42, 130.51, 128.31, 128.25, 127.38, 126.27, 124.97, 121.14 (q, *J* = 258.8 Hz), 87.04, 42.22 and 35.52 ppm. ^19^F-NMR (282 MHz, CDCl_3_) δ: −51.67 (s, 3F) ppm. IR (NaCl): *ν* = 3033, 2929, 1730, 1608, 1496, 1456, 1265, 1043, 757 and 701 cm^−1^. HRMS (ESI) *m/z*: [M + Na]^+^ Calcd. for C_17_H_13_F_3_NaO_2_^+^ 329.0760; found 329.0765.

*(+)-2-(4-Fluorobenzyl)-2-(trifluoromethoxy)-2,3-dihydro-1H-inden-1-one* ((+)-**3ab**). The reaction was run according to the general procedure. Eluent (*n*-hexane/ethyl acetate = 15/1). Slightly yellow oil; 26.9 mg; 83% yield. The enantiomeric excess (53% ee) was determined by chiral HPLC using CHIRALCEL^®^ OJ-H (*n*-hexane/isopropanol = 99.0/1.0, flow rate 1.0 mL/min, λ = 254 nm) t (major) = 19.383 min, t (minor) = 11.833 min). ${\left[\alpha \right]}_{D}^{25}$ = +29.6 (CH_2_Cl_2_, *c* = 0.76). ^1^H-NMR (300 MHz, CDCl_3_) δ: 7.77 (d, *J* = 7.7 Hz, 1H), 7.61 (t, *J* = 7.5 Hz, 1H), 7.41–7.33 (m, 2H), 7.17–7.12 (m, 2H), 6.91 (t, *J* = 8.4 Hz, 2H), 3.48–3.35 (m, 2H), 3.27 (d, *J* = 14.0 Hz, 1H) and 3.00 (d, *J* = 14.0 Hz, 1H) ppm. ^13^C-NMR (126 MHz, CDCl_3_) δ: 199.84, 162.16 (d, *J* = 246.2 Hz), 149.37, 136.30, 133.36, 132.02 (d, *J* = 8.0 Hz), 129.50 (d, *J* = 3.2 Hz), 128.38, 126.29, 124.98, 121.12 (q, *J* = 258.8 Hz), 115.24 (d, *J* = 21.4 Hz), 86.87, 41.50 and 35.56 ppm. ^19^F-NMR (282 MHz, CDCl_3_) δ: −51.72 (s, 3F) and −115.49–−115.59 (m, 1F) ppm. IR (NaCl): *ν* = 3045, 2931, 1730, 1606, 1512, 1469, 1265, 1159, 838 and 744 cm^−1^. HRMS (ESI) *m/z*: [M + Na]^+^ Calcd. for C_17_H_12_F_4_NaO_2_^+^ 347.0666; found 347.0669.

*(+)-2-(4-Bromobenzyl)-2-(trifluoromethoxy)-2,3-dihydro-1H-inden-1-one* ((+)-**3ac**). The reaction was run according to the general procedure. Eluent (*n*-hexane/ethyl acetate = 15/1). Slightly yellow semi-solid; 28.9 mg; 75% yield. The enantiomeric excess (41% ee) was determined by chiral HPLC using CHIRALCEL^®^ OJ-H (*n*-hexane/isopropanol = 99.0/1.0, flow rate 1.0 mL/min, λ = 254 nm) t (major) = 33.433 min, t (minor) = 13.825 min). ${\left[\alpha \right]}_{D}^{25}$ = +22.8 (CH_2_Cl_2_, *c* = 0.84). ^1^H-NMR (300 MHz, CDCl_3_) δ: 7.79 (d, *J* = 7.7 Hz, 1H), 7.62 (t, *J* = 7.5 Hz, 1H), 7.43–7.34 (m, 4H), 7.07 (d, *J* = 8.2, 2H), 3.47–3.33 (m, 2H), 3.25 (d, *J* = 14.0 Hz, 1H) and 2.95 (d, *J* = 14.0 Hz, 1H) ppm. ^13^C-NMR (126 MHz, CDCl_3_) δ: 199.66, 149.29, 136.37, 133.22, 132.86, 132.16, 131.47, 128.45, 126.35, 125.07, 121.61, 121.08 (q, *J* = 259.0 Hz), 86.63, 41.65 and 35.54 ppm. ^19^F-NMR (282 MHz, CDCl_3_) δ: −51.75 (s, 3F) ppm. IR (NaCl): *ν* = 3074, 2929, 1730, 1608, 1489, 1265, 1201, 1153, 1012 and 519 cm^−1^. HRMS (ESI) *m/z*: [M + Na]^+^ Calcd. for C_17_H_12_BrF_3_NaO_2_^+^ 406.9865; found 406.9865.

*(+)-2-(Trifluoromethoxy)-2-(4-(trifluoromethyl)benzyl)-2,3-dihydro-1H-inden-1-one* ((+)-**3ad**). The reaction was run according to the general procedure. Eluent (*n*-hexane/ethyl acetate = 15/1). Slightly yellow oil; 23.6 mg; 63% yield. The enantiomeric excess (40% ee) was determined by chiral HPLC using CHIRALCEL^®^ OJ-H (*n*-hexane/isopropanol = 99.0/1.0, flow rate 1.0 mL/min, λ = 254 nm) t (major) = 48.208 min, t (minor) = 33.525 min). ${\left[\alpha \right]}_{D}^{25}$ = +18.8 (CH_2_Cl_2_, *c* = 0.50). ^1^H-NMR (300 MHz, CDCl_3_) δ: 7.80 (d, *J* = 7.7 Hz, 1H), 7.63 (t, *J* = 7.5, 1H), 7.51 (d, *J* = 8.0 Hz, 2H), 7.42 (t, *J* = 7.4 Hz, 1H), 7.39–7.32 (m, 3H), 3.49–3.34 (m, 3H) and 3.04 (d, *J* = 13.9 Hz, 1H) ppm. ^13^C-NMR (126 MHz, CDCl_3_) δ: 199.42, 149.15, 138.06, 136.45, 133.13, 130.87, 129.72 (q, *J* = 32.5 Hz), 128.54, 126.39, 125.25 (q, *J* = 3.7 Hz), 125.15, 124.00 (q, *J* = 272.1 Hz), 121.07 (q, *J* = 259.2 Hz), 86.57, 42.03 and 35.65 ppm. ^19^F-NMR (282 MHz, CDCl_3_) δ: −51.82 (s, 3F) and −63.14 (s, 3F) ppm. IR (NaCl): *ν* = 3076, 2937, 1732, 1610, 1419, 1327, 1267, 1162, 1068 and 748 cm^−1^. HRMS (ESI) *m/z*: [M + Na]^+^ Calcd. for C_18_H_12_F_6_NaO_2_^+^ 397.0634; found 397.0638.

*(+)-2-(3-Fluorobenzyl)-2-(trifluoromethoxy)-2,3-dihydro-1H-inden-1-one* ((+)-**3ae**). The reaction was run according to the general procedure. Eluent (*n*-hexane/ethyl acetate = 15/1). Slightly yellow oil; 24.3 mg; 75% yield. The enantiomeric excess (47% ee) was determined by chiral HPLC using CHIRALCEL^®^ OJ-H (*n*-hexane/isopropanol = 99.0/1.0, flow rate 1.0 mL/min, λ = 254 nm) t (major) = 14.050 min, t (minor) = 8.758 min). ${\left[\alpha \right]}_{D}^{25}$ = +26.8 (CH_2_Cl_2_, *c* = 0.19). ^1^H-NMR (300 MHz, CDCl_3_) δ: 7.80 (d, *J* = 7.9 Hz, 1H), 7.65–7.59 (m, 1H), 7.43–7.35 (m, 2H), 7.25–7.17 (m, 1H), 6.98–6.89 (m, 3H), 3.50–3.36 (m, 2H), 3.31 (d, *J* = 13.9 Hz, 1H) and 2.99 (d, *J* = 13.9 Hz, 1H) ppm. ^13^C-NMR (126 MHz, CDCl_3_) δ: 199.66, 162.49 (d, *J* = 246.1 Hz), 149.33, 136.33, 136.27 (d, *J* = 7.4 Hz), 133.26, 129.79 (d, *J* = 8.4 Hz), 128.42, 126.34, 126.25 (d, *J* = 2.9 Hz), 125.07, 121.09 (q, *J* = 258.9 Hz), 117.40 (d, *J* = 21.6 Hz), 114.43 (d, *J* = 20.9 Hz), 86.72, 41.90 and 35.58 ppm. ^19^F-NMR (282 MHz, CDCl_3_) δ: −51.75 (s, 3F) and −113.50–−113.58 (m, 1F) ppm. IR (NaCl): *ν* = 3070, 2931, 1732, 1610, 1489, 1448, 1265, 1149 and 785 cm^−1^. HRMS (ESI) *m/z*: [M + Na]^+^ Calcd. for C_17_H_12_F_4_NaO_2_^+^ 347.0666; found 347.0672.

*(+)-2-([1,1′-Biphenyl]-4-ylmethyl)-2-(trifluoromethoxy)-2,3-dihydro-1H-inden-1-one* ((+)-**3af**). The reaction was run according to the general procedure. Eluent (*n*-hexane/ethyl acetate = 15/1). Slightly yellow oil; 31.0 mg; 81% yield. The enantiomeric excess (41% ee) was determined by chiral HPLC using CHIRALPAK^®^ ID (*n*-hexane/isopropanol = 99.0/1.0, flow rate 1.0 mL/min, λ = 254 nm) t (major) = 10.408 min, t (minor) = 8.192 min). ${\left[\alpha \right]}_{D}^{25}$ = +27.3 (CH_2_Cl_2_, *c* = 0.84). ^1^H-NMR (300 MHz, CDCl_3_) δ: 7.80 (d, *J* = 7.7 Hz, 1H), 7.62–7.31 (m, 10H), 7.27–7.25 (m, 2H), 3.47 (s, 2H), 3.36 (d, *J* = 13.9 Hz, 1H) and 3.05 (d, *J* = 13.9 Hz, 1H) ppm. ^13^C-NMR (126 MHz, CDCl_3_) δ: 200.04, 149.54, 140.49, 140.19, 136.21, 133.38, 132.89, 130.93, 128.75, 128.30, 127.34, 126.99, 126.96, 126.33, 125.01, 121.16 (q, *J* = 258.8 Hz), 87.06, 41.88 and 35.61 ppm. ^19^F-NMR (282 MHz, CDCl_3_) δ: −51.64 (s, 3F) ppm. IR (NaCl): *ν* = 3032, 2937, 1730, 1608, 1487, 1265, 1201, 1151 and 748 cm^−1^. HRMS (ESI) *m/z*: [M + Na]^+^ Calcd. for C_23_H_17_F_3_NaO_2_^+^ 405.1073; found 405.1077.

*(+)-2-(Naphthalen-2-ylmethyl)-2-(trifluoromethoxy)-2,3-dihydro-1H-inden-1-one* ((+)-**3ag**). The reaction was run according to the general procedure. Eluent (*n*-hexane/ethyl acetate = 15/1). Slightly yellow oil; 30.3 mg; 85% yield. The enantiomeric excess (24% ee) was determined by chiral HPLC using CHIRALPAK ^®^ ID (*n*-hexane/isopropanol = 99.5/0.5, flow rate 1.0 mL/min, λ = 254 nm) t (major) = 9.708 min, t (minor) = 8.233 min). ${\left[\alpha \right]}_{D}^{25}$ = +18.1 (CH_2_Cl_2_, *c* = 0.77). ^1^H-NMR (300 MHz, CDCl_3_) δ: 7.82–7.26 (m, 11H), 3.54–3.39 (m, 3H) and 3.16 (d, *J* = 13.9 Hz, 1H) ppm. ^13^C-NMR (126 MHz, CDCl_3_) δ: 200.07, 149.55, 136.21, 133.33, 133.10, 132.47, 131.57, 129.53, 128.39, 128.29, 127.98, 127.65, 127.56, 126.33, 126.14, 125.95, 125.02, 121.16 (q, *J* = 258.9 Hz), 87.15, 42.26 and 35.50 ppm. ^19^F-NMR (282 MHz, CDCl_3_) δ: −51.64 (s, 3F) ppm. IR (NaCl): *ν* = 3057, 2927, 1730, 1608, 1509, 1468, 1263, 1153, 1162, 1045 and 742 cm^−1^. HRMS (ESI) *m/z*: [M + Na]^+^ Calcd. for C_21_H_15_F_3_NaO_2_^+^ 379.0916; found 379.0917.

*(+)-2-(3,5-di-tert-Butylbenzyl)-2-(trifluoromethoxy)-2,3-dihydro-1H-inden-1-one* ((+)-**3ah**). The reaction was run according to the general procedure. Eluent (n-hexane/ethyl acetate = 20/1). Yellow oil; 33.1 mg; 79% yield. The enantiomeric excess (37% ee) was determined by chiral HPLC using CHIRALPAK^®^ IF column (*n*-hexane/TBME = 95.0/5.0, flow rate 0.5 mL/min, λ = 254 nm) t (major) = 12.842 min, t (minor) = 11.972 min). ${\left[\alpha \right]}_{D}^{25}$ = +24.3 (CH_2_Cl_2_, *c* = 0.87). ^1^H-NMR (300 MHz, CDCl_3_) δ: 7.69 (d, *J* = 7.8 Hz, 1H), 7.51 (t, *J* = 7.5 Hz, 1H), 7.32–7.15 (m, 3H), 6.94 (s, 2H), 3.45 (s, 2H), 3.30 (d, *J* = 13.3 Hz, 1H), 3.14 (d, *J* = 13.3 Hz, 1H) and 1.22 (s, 18H) ppm. ^13^C-NMR (126 MHz, CDCl_3_) δ: 200.38, 150.55, 149.71, 135.88, 133.85, 132.40, 127.95, 125.99, 124.70, 124.59, 121.26 (q, *J* = 258.5k Hz), 120.95, 87.41, 43.01, 35.97, 35.96, 34.61 and 31.30 ppm. ^19^F-NMR (282 MHz, CDCl_3_) δ: −51.56 (s, 3F) ppm. IR (NaCl): *ν* = 2961, 2867, 2359, 2336, 1730, 1465, 1368, 1263, 1199, 1153 and 830,720 cm^−1^. MS (ESI): *m/z* 436 [M + NH_4_]^+^.

*(+)-2-Benzyl-5-bromo-2-(trifluoromethoxy)-2,3-dihydro-1H-inden-1-one* ((+)-**3ba**). The reaction was run according to the general procedure. Eluent (*n*-hexane/ethyl acetate = 15/1). Slightly yellow solid; *35.8* mg; *93*% yield. The enantiomeric excess (57% ee) was determined by chiral HPLC using CHIRALCEL^®^ OJ-H (*n*-hexane/isopropanol = 99.0/1.0, flow rate 1.0 mL/min, λ = 254 nm) t (major) = 13.833 min, t (minor) = 18.725 min). ${\left[\alpha \right]}_{D}^{25}$ = +12.5 (CH_2_Cl_2_, *c* = 0.31). ^1^H-NMR (300 MHz, CDCl_3_) δ: 7.63 (d, *J* = 8.4 Hz, 1H), 7.53–7.51 (m, 2H), 7.32–7.06 (m, 5H), 3.41 (s, 2H), 3.30 (d, *J* = 13.8 Hz, 1H) and 3.02 (d, *J* = 13.8 Hz, 1H) ppm. ^13^C-NMR (126 MHz, CDCl_3_) δ: 198.92, 151.00, 133.42, 132.26, 131.99, 131.66, 130.47, 129.58, 128.43, 127.55, 126.09, 121.09 (q, *J* = 259.1 Hz), 86.77, 42.15 and 35.21 ppm. ^19^F-NMR (282 MHz, CDCl_3_)) δ: −51.71 (s, 3F) ppm. IR (NaCl): *ν* = 3031, 2929, 1734, 1263, 1203, 1151, 1058, 704 and 573 cm^−1^. HRMS (ESI) *m/z*: [M + Na]^+^ Calcd. for C_17_H_12_BrF_3_NaO_2_^+^ 406.9865; found 406.9864. m.p.: 53.6–55.8 °C.

*(+)-2-Benzyl-5-fluoro-2-(trifluoromethoxy)-2,3-dihydro-1H-inden-1-one* ((+)-**3ca**). The reaction was run according to the general procedure. Eluent (*n*-hexane/ethyl acetate = 15/1). Slightly yellow oil; 23.0 mg; 71% yield. The enantiomeric excess (51% ee) was determined by chiral HPLC using CHIRALCEL^®^ OJ-H (*n*-hexane/isopropanol = 99.0/1.0, flow rate 1.0 mL/min, λ = 254 nm) t (major) = 28.833 min, t (minor) = 27.167 min). ${\left[\alpha \right]}_{D}^{25}$ = +27.0 (CH_2_Cl_2_, *c* = 0.77). ^1^H-NMR (300 MHz, CDCl_3_) δ: 7.81–7.76 (m, 1H), 7.23–7.17 (m, 5H), 7.07 (t, *J* = 8.8 Hz, 1H), 6.99 (d, *J* = 8.2 Hz, 1H), 3.42 (s, 2H), 3.31 (d, *J* = 13.8 Hz, 1H) and 3.05 (d, *J* = 13.8 Hz, 1H) ppm. ^13^C-NMR (126 MHz, CDCl_3_) δ: 198.16, 167.78 (d, *J* = 259.1 Hz), 152.48 (d, *J* = 10.5 Hz), 133.47, 130.46, 129.94 (d, *J* = 1.9 Hz), 128.39, 127.51, 127.48 (d, *J* = 10.5 Hz), 121.12 (q, *J* = 259.0 Hz), 116.73 (d, *J* = 23.7 Hz), 113.09 (d, *J* = 22.7 Hz), 86.97, 42.20 and 35.58 ppm. ^19^F-NMR (282 MHz, CDCl_3_) δ: −51.72 (s, 3F) and −99.80 (q, *J* = 7.1 Hz, 1F) ppm. IR (NaCl): ν = 3028, 2929, 1734, 1616, 1595, 1263, 1200, 1151 and 702 cm^−1^. HRMS (ESI) *m/z*: [M + Na]^+^ Calcd. for C_17_H_12_F_4_NaO_2_^+^ 347.0666; found 347.0672.

*(+)-2-Benzyl-6-fluoro-2-(trifluoromethoxy)-2,3-dihydro-1H-inden-1-one* ((+)-**3da**). The reaction was run according to the general procedure. Eluent (*n*-hexane/ethyl acetate = 15/1). Slightly yellow oil; 26.6 mg; 82% yield. The enantiomeric excess (56% ee) was determined by chiral HPLC using CHIRALCEL^®^ OJ-H (*n*-hexane/isopropanol = 99.0/1.0, flow rate 1.0 mL/min, λ = 254 nm) t (major) = 13.792 min, t (minor) = 9.725 min). ${\left[\alpha \right]}_{D}^{25}$ = +22.2 (CH_2_Cl_2_, *c* = 0.80). ^1^H-NMR (300 MHz, CDCl_3_) δ: 7.40 (d, *J* = 7.0 Hz, 1H), 7.29 (d, *J* = 5.1 Hz, 2H), 7.23–7.14 (m, 5H), 3.40 (s, 2H), 3.30 (d, *J* = 13.8 Hz, 1H) and 3.05 (d, *J* = 13.7 Hz, 1H) ppm. ^13^C-NMR (126 MHz, CDCl_3_) δ: 199.35, 162.43 (d, *J* = 249.8 Hz), 145.02 (d, *J* = 2.3 Hz), 135.14 (d, *J* = 7.5 Hz), 133.45, 130.44, 128.38, 127.84 (d, *J* = 7.8 Hz), 127.51, 123.92 (d, *J* = 23.7 Hz), 121.12 (q, *J* = 258.9 H z), 110.69 (d, *J* = 22.2 Hz), 87.49, 42.27 and 35.10 ppm. ^19^F-NMR (282 MHz, CDCl_3_) δ: −51.73 (s, 3F) and −112.86 (q, *J* = 6.9 Hz, 1F) ppm. IR (NaCl): *ν* = 3033, 2947, 1736, 1614, 1489, 1265, 1200, 1155, 775 and 702 cm^−1^. HRMS (ESI) *m/z*: [M + Na]^+^ Calcd. for C_17_H_12_F_4_NaO_2_^+^ 347.0666; found 347.0671.

*(+)-2-Benzyl-6-methyl-2-(trifluoromethoxy)-2,3-dihydro-1H-inden-1-one* ((+)-**3ea**). The reaction was run according to the general procedure. Eluent (*n*-hexane/ethyl acetate = 15/1). Slightly yellow oil; 27.2 mg; 85% yield. The enantiomeric excess (29% ee) was determined by chiral HPLC using CHIRALCEL^®^ OJ-H (*n*-hexane/isopropanol = 99.0/1.0, flow rate 1.0 mL/min, λ = 254 nm) t (major) = 17.217 min, t (minor) = 8.958 min). ${\left[\alpha \right]}_{D}^{25}$ = +20.0 (CH_2_Cl_2_, *c* = 0.82). ^1^H-NMR (300 MHz, CDCl_3_) δ: 7.58 (s, 1H), 7.41 (d, *J* = 7.8 Hz, 1H), 7.26–7.17 (m, 6H), 3.37 (s, 2H), 3.30 (d, *J* = 13.8 Hz, 1H), 2.98 (d, *J* = 13.9 Hz, 1H) and 2.38 (s, 3H) ppm. ^13^C-NMR (126 MHz, CDCl_3_) δ: 200.12, 146.90, 138.35, 137.45, 133.98, 133.47, 130.54, 128.29, 127.33, 125.98, 124.84, 121.14 (q, *J* = 258.5 Hz), 87.37, 42.22, 35.14 and 21.11 ppm. ^19^F-NMR (282 MHz, CDCl_3_) δ: −51.68 (s, 3F) ppm. IR (NaCl): *ν* = 3034, 2929, 1727, 1495, 1265, 1153 and 702 cm^−1^. HRMS (ESI) *m/z*: [M + Na]^+^ Calcd. for C_18_H_15_F_3_NaO_2_^+^ 343.0916; found 343.0913.

*(+)-2-Benzyl-6-methoxy-2-(trifluoromethoxy)-2,3-dihydro-1H-inden-1-one* ((+)-**3fa**). The reaction was run according to the general procedure. Eluent (*n*-hexane/ethyl acetate = 10/1). Yellow solid; 30.9 mg; 92% yield. The enantiomeric excess (33% ee) was determined by chiral HPLC using CHIRALCEL^®^ OJ-H (*n*-hexane/isopropanol = 99.0/1.0, flow rate 1.0 mL/min, λ = 254 nm) t (major) = 26.942 min, t (minor) = 16.517 min). ${\left[\alpha \right]}_{D}^{25}$ = +29.2 (CH_2_Cl_2_, *c* = 0.67). ^1^H-NMR (300 MHz, CDCl_3_) δ: 7.26–7.17 (m, 8H), 3.83 (s, 3H), 3.35 (s, 2H), 3.30 (d, *J* = 13.8 Hz, 1H) and 3.00 (d, *J* = 13.8 Hz, 1H) ppm. ^13^C-NMR (126 MHz, CDCl_3_) δ: 200.13, 159.80, 142.42, 134.48, 133.91, 130.50, 128.29, 127.37, 127.09, 125.69, 121.14 (q, *J* = 258.8 Hz), 105.79, 87.65, 55.64, 42.33 and 34.87 ppm. ^19^F-NMR (282 MHz, CDCl_3_) δ: −51.73 (s, 3F) ppm. IR (NaCl): *ν* = 3032, 2945, 1728, 1618, 1493, 1435, 1271, 1028, 769 and 702 cm^−1^. HRMS (ESI) *m/z*: [M + Na]^+^ Calcd. for C_18_H_15_F_3_NaO_3_^+^ 359.0866; found 359.0869. m.p.: 93.5–95.7 °C.

*(+)-2-Benzyl-5,6-dimethoxy-2-(trifluoromethoxy)-2,3-dihydro-1H-inden-1-one* ((+)-**3ga**). The reaction was run according to the general procedure. Eluent (*n*-hexane/ethyl acetate = 5/1). Orange solid; 24.9 mg; 68% yield. The enantiomeric excess (13% ee) was determined by chiral HPLC using a series of CHIRALPAK^®^ IF and CHIRALPAK^®^ IA-3 (*n*-hexane/isopropanol = 90.0/10.0, flow rate 1.0 mL/min, λ = 254 nm) t (major) = 15.475 min, t (minor) = 23.808 min). ${\left[\alpha \right]}_{D}^{25}$ = −4.5 (CH_2_Cl_2_, *c* = 0.83). ^1^H-NMR (300 MHz, CDCl_3_) δ: 7.27–7.18 (m, 6H), 6.72 (s, 1H), 3.93 (s, 3H), 3.90 (s, 3H), 3.34–3.29 (m, 3H) and 3.02 (d, *J* = 13.8 Hz, 1H) ppm. ^13^C-NMR (126 MHz, CDCl_3_) δ: 198.42, 156.63, 149.97, 145.30, 134.08, 130.51, 128.27, 127.32, 126.22, 121.16 (q, *J* = 258.5 Hz), 107.02, 104.96, 87.37, 56.34, 56.14, 42.31 and 35.24 ppm. ^19^F-NMR (282 MHz, CDCl_3_) δ: −51.72 (s, 3F) ppm. IR (NaCl): ν = 3030, 2939, 1716, 1591, 1502, 1268, 1196, 1146, 782 and 702 cm^−1^. HRMS (ESI) *m/z*: [M + Na]^+^ Calcd. for C_19_H_17_F_3_NaO_4_^+^ 389.0971; found 389.0976. m.p.: 101.4–105.2 °C.

*(+)-2-Benzyl-2-(trifluoromethoxy)-3,4-dihydronaphthalen-1(2H)-one* ((+)-**3ha**). The reaction was run according to the general procedure. Eluent (*n*-hexane/ethyl acetate = 10/1). Slightly yellow oil; 16.3 mg; 51% yield. The enantiomeric excess (8% ee) was determined by chiral HPLC using CHIRALCEL^®^ OJ-H (*n*-hexane/isopropanol = 99.0/1.0, flow rate 1.0 mL/min, λ=254 nm) t (major) = 22.442 min, t (minor) = 8.583 min). ${\left[\alpha \right]}_{D}^{25}$ = −0.1 (CH_2_Cl_2_, *c* = 0.30). ^1^H-NMR (300 MHz, CDCl_3_) δ: 8.13 (dd, *J* = 7.8, 1.5 Hz, 1H), 7.55 (td, *J* = 7.5, 1.5 Hz, 1H), 7.38 (t, *J* = 7.5 Hz, 1H), 7.35–7.26 (m, 6H), 3.29–3.16 (m, 2H), 3.11 (t, *J* = 6.3 Hz, 2H), 2.49 (dt, *J* = 14.1, 7.1 Hz, 1H) and 2.14 (dt, *J* = 13.7, 5.1 Hz, 1H) ppm. ^13^C-NMR (126 MHz, CDCl_3_) δ: 192.65, 142.15, 134.27, 134.25, 130.68, 130.63, 128.71, 128.68, 128.38, 127.33, 127.25, 121.09 (q, *J* = 258.4 Hz), 85.72, 39.78, 30.41 and 26.01 ppm. ^19^F-NMR (282 MHz, CDCl_3_) δ: −50.57 (s, 3F) ppm. IR (NaCl): *ν* = 3032, 2949, 1699, 1603, 1454, 1273, 1201, 1146, 908 and 706 cm^−1^. HRMS (ESI) *m/z*: [M + Na]^+^ Calcd. for C_18_H_15_F_3_NaO_2_^+^ 343.0916; found 343.0922.

*(R)-2-Allyl-2-(trifluoromethoxy)-2,3-dihydro-1H-inden-1-one* (**3ai**). The reaction was run according to the general procedure, and the product is consistent with previously reported characterization data [46]. Eluent (*n*-hexane/ethyl acetate = 15/1). Colorless oil; 11.0 mg; 43% yield. The enantiomeric excess (68% ee) was determined by chiral HPLC using a CHIRALCEL^®^ OJ-H column (*n*-hexane/isopropanol = 98.0/2.0, flow rate 0.5 mL/min, λ = 254 nm) t (major) = 11.167 min, t (minor) = 9.975 min). ${\left[\alpha \right]}_{D}^{25}$ = +16.5 (CHCl_3_, *c =* 0.37). ^1^H-NMR (300 MHz, CDCl_3_) δ: 7.82 (d, *J* = 7.7 Hz, 1H), 7.68 (t, *J* = 7.5 Hz, 1H), 7.46–7.42 (m, 2H), 5.67 (ddt, *J* = 17.3, 10.4, 7.2 Hz, 1H), 5.19–5.11 (m, 2H), 3.54 (d, *J* = 17.8 Hz, 1H), 3.41 (d, *J* = 17.8 Hz, 1H), 2.75 (dd, *J* = 14.0, 6.7 Hz, 1H), 2.55 (dd, *J* = 14.0, 7.5 Hz, 1H) ppm. ^19^F-NMR (282 MHz, CDCl_3_) δ: −51.90 (s, 3F) ppm.

*(R)-2-Allyl-5-fluoro-2-(trifluoromethoxy)-2,3-dihydro-1H-inden-1-one* (**3ci**). The reaction was run according to the general procedure, and the product is consistent with previously reported characterization data. [46] Eluent (*n*-hexane/ethyl acetate = 20/1). Colorless oil; 9.6 mg; 35% yield. The enantiomeric excess (70% ee) was determined by chiral HPLC using CHIRALPAK^®^ IF column (*n*-hexane/TBME = 90.0/10.0, flow rate 0.5 mL/min, λ = 254 nm) t (major) = 15.192 min, t (minor) = 13.042 min). ${\left[\alpha \right]}_{D}^{25}$ = +19.7 (CH_2_Cl_2_, *c* = 0.31). ^1^H-NMR (300 MHz, CDCl_3_) δ: 7.85 (d, *J* = 4.5 Hz, 1H), 7.18–7.12 (m, 2H), 5.67–5.60 (m, 1H), 5.21–5.12 (m, 2H), 3.53 (d, *J* = 18.0 Hz, 1H), 3.40 (d, *J* = 18.1 Hz, 1H), 2.79–2.72 (m, 1H), 2.60–2.52 (m, 1H) ppm. ^19^F-NMR (282 MHz, CDCl_3_) δ: −51.95 (s, 3F) and −99.62–−99.73 (m, 1F) ppm. 

*(R)-2-Allyl-6-methyl-2-(trifluoromethoxy)-2,3-dihydro-1H-inden-1-one* (**3ei**). The reaction was run according to the general procedure, and the product was consistent with previously reported characterization data [46]. Eluent (*n*-hexane/ethyl acetate = 15/1). Colorless oil; 14.0 mg; 52% yield. The enantiomeric excess (45% ee) was determined by chiral HPLC using CHIRALPAK^®^ IF column (*n*-hexane/TBME = 90.0/10.0, flow rate 0.5 mL/min, λ = 254 nm) t (major) = 15.992 min, t (minor) = 13.083 min). ${\left[\alpha \right]}_{D}^{25}$ = +16.2 (CH_2_Cl_2_, *c* = 0.46). ^1^H-NMR (300 MHz, CDCl_3_) δ: 7.61 (s, 1H), 7.49 (d, *J* = 7.9 Hz, 1H), 7.32 (d, *J* = 7.7 Hz, 1H), 5.70–5.59 (m, 1H), 5.18– 5.10 (m, 2H), 3.48 (d, *J* = 17.7 Hz, 1H), 3.35 (d, *J* = 17.5 Hz, 1H), 2.76–2.69 (m, 1H), 2.57–2.50 (m, 1H) and 2.41 (s, 3H) ppm. ^19^F-NMR (282 MHz, CDCl_3_) δ: −51.90 (s, 3F) ppm.

## 4. Conclusions

In conclusion, we disclose the organo-catalytic enantioselective benzylation reaction of α-OCF_3_-indanones **1**. α-Benzyl-α-OCF_3_-indanones **3** was synthesized in good to high yield with moderate enantioselectivity, up to 57% ee, and both enantiomers of **3** could be accessed by the selection of chiral PTC, CN-4 or CD-4. The method was extended to the enantioselective allylation of **1**, and both enantiomers of α-allyl-α-OCF_3_-indanones were also obtained in moderate yield with good ee, as much as 76% ee. To our knowledge, this is the first example of the asymmetric synthesis of trifluoromethoxylated compounds with a stereogenic OCF_3_-carbon center, without the use of transition metals. Extension of this methodology to other OCF_3_ ketones is underway, and will be reported in due course [48].

## Figures and Tables

**Table 1:**
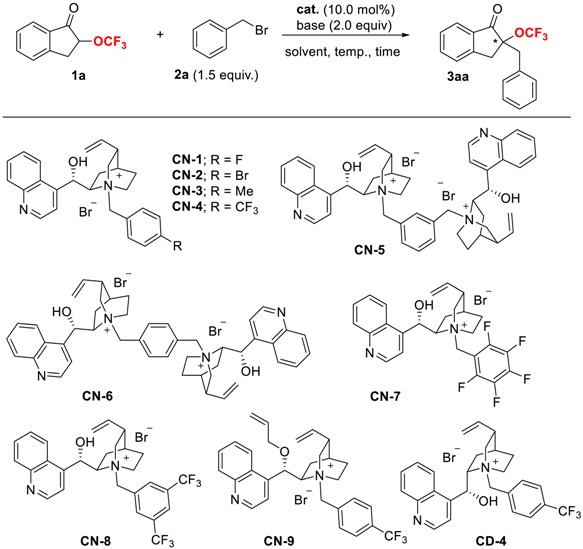
Optimal condition screening ^1^.

Entry	Cat.	Base	Solvent	Time	Yield (%) ^7^	ee (%) ^8^
1	CN-1	KOH	Toluene	15	73	1
2	CN-2	KOH	Toluene	15	75	3
3	CN-3	KOH	Toluene	15	82	14
4	CN-4	KOH	Toluene	15	88	26
5	CN-5	KOH	Toluene	15	82	14
6	CN-6	KOH	Toluene	15	89	0
7	CN-7	KOH	Toluene	15	67	0
8	CN-8	KOH	Toluene	15	78	0
9	CN-4	KOH	*n*-hexane	24	38	2
10	CN-4	KOH	DCM	24	65	5
11	CN-4	KOH	THF	24	68	5
12	CN-4	KOH	Et_2_O	24	40	19
13	CN-4	KOH	DMF	15	20	1
14	CN-4	K_2_CO_3_	Toluene	48	11	n.d.
15	CN-4	NaOH	Toluene	48	58	4
16	CN-4	CsOH·H_2_O	Toluene	15	83	43
17	CN-4	LiOH	Toluene	48	NR	-
18	CN-4	NaH	Toluene	48	57	0
19	CN-4	KOAc	Toluene	48	NR	-
20	CN-4	K_2_HPO_4_	Toluene	48	NR	-
21	CN-4	KO*t*Bu	Toluene	24	26	17
22	CN-4	KOMe	Toluene	24	6	n.d.
23	CN-4	50% KOH (aq)	Toluene	15	44	6
24 ^2^	CN-4	KOH	Toluene	15	68	24
25	CN-4	CsOH·H_2_O	PhCF_3_	15	96	9
26	CN-4	CsOH·H_2_O	Toluene:CHCl_3_ = 7:3	24	39	35
27	CN-9	CsOH·H_2_O	Toluene	15	77	1
28 ^3^	CN-4	CsOH·H_2_O	Toluene	15	92	49
29 ^4^	CN-4	CsOH·H_2_O	Toluene	15	83	50
30 ^5^	CN-4	CsOH·H_2_O	Toluene	15	80	54
31 ^5,6^	CN-4	CsOH·H_2_O	Toluene	72	75	57
32 ^5^	CD-4	CsOH·H_2_O	Toluene	15	75	−50

^1^ Reaction conditions: **1a** (0.1 mmol, 1.0 equiv), BnBr **2a** (0.15 mmol 1.5 equiv), base (0.2 mmol, 2.0 equiv) and cat. (10.0 mol%) were stirred in 1.0 mL of anhydrous toluene at room temperature. ^2^ H_2_O (2.0 equiv) was added. ^3^ 2.0 mL of toluene was used. ^4^ 3.0 mL of toluene was used. ^5^ 5.0 mL of toluene was used. ^6^ Stirred at 0 °C. ^7^ Isolated yields. ^8^ ee was determined by chiral HPLC. CN = cinchonine, CD = cinchonidine.

## Supplementary Materials

The following are available online, ^1^H, ^13^C and ^19^F-NMR spectra for desired compounds **3** and HPLC data for desired compounds **3**.
