# Supplementary material for: Enantioselective Benzylation and Allylation of α-Trifluoromethoxy Indanones under Phase-Transfer Catalysis
Source: Molecules. 2019 Jul 30;24(15):2774. doi: 10.3390/molecules24152774 (PMC6696116; doi:10.3390/molecules24152774)
Supplement: Supplementary file 1 [file molecules-24-02774-s001.pdf]

Electronic Supplementary Information

Enantioselective Benzylation and Allylation of α-Trifluoromethoxy Indanones under Phase-Transfer Catalysis

Yumeng Liang [1], Mayaka Maeno [1], Zhengyu Zhao [1] and Norio Shibata [1,2,*]

[1] Department of Nanopharmaceutical Sciences, Department of Life Science and Applied Chemistry, Nagoya Institute of Technology Gokiso, Showa-ku, Nagoya 466-8555, Japan

[2] Institute of Advanced Fluorine-Containing Materials, Zhejiang Normal University, 688 Yingbin Avenue, Jinhua 321004, China.

* Correspondence: nozshiba@nitech.ac.jp; Tel. /Fax: +81-52-735-7543

Table of Contents

1. Optimization of Reaction Condition 3

2.^{1}H, ^{13}C and ^{19}F NMR spectra for desired compounds 3 4

3. HPLC data for desired compounds (+)-3. 30

4. HPLC data for desired compounds (−)-3 in Scheme 3. 37

5. HPLC data for desired compounds 3 in Scheme 4. 40

1. Optimization of Reaction Condition

Table S1. Optimal condition screening [1]

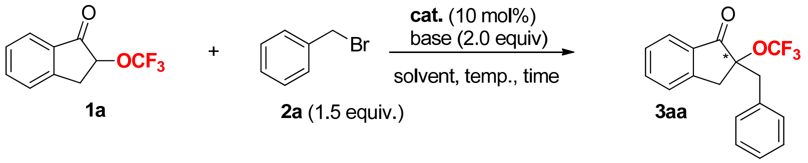

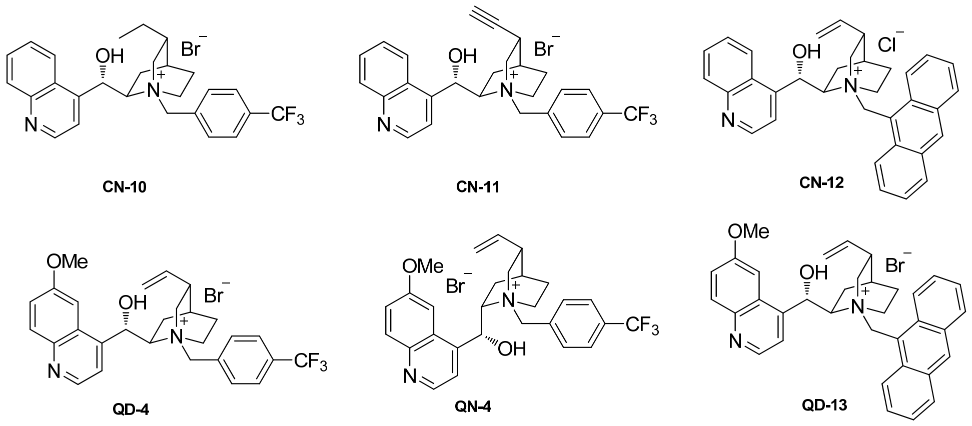

Entry	Cat.	Base	Solvent	Time	Yield (%)[2]	Ee (%)[3]
1	TBAB	KOH	toluene	15	70	-
2	-	KOH	toluene	24	trace	-
3	CN-10	$CsOH{\cdot}H_2O$	toluene	15	88	36
4	CN-11	$CsOH{\cdot}H_2O$	toluene	12	93	26
5	CN-12	$CsOH{\cdot}H_2O$	toluene	15	16	19
6	QD-4	$CsOH{\cdot}H_2O$	toluene	15	67	49
7	QN-4	$CsOH{\cdot}H_2O$	toluene	15	71	-50
8	QD-13	$CsOH{\cdot}H_2O$	toluene	15	18	17

[1] Reaction conditions: **1a** (0.1mmol, 1.0 equiv.), BnBr **2a** (0.15mmol 1.5 equiv.), base (0.2mmol, 2.0 equiv.) and **cat.** (10.0 mol%) were stirred in anhydrous toluene 5.0 mL at room temperature. [2] Isolated yields. [3] Ee was determined by chiral HPLC. CN = Cinchonine, QD = Quinidine, QN = Quinine.

2. ^{1}H, ^{13}C and ^{19}F NMR spectra for desired compounds 3

*2-Benzyl-2-(trifluoromethoxy)-2,3-dihydro-1H-inden-1-one (**3aa**).*

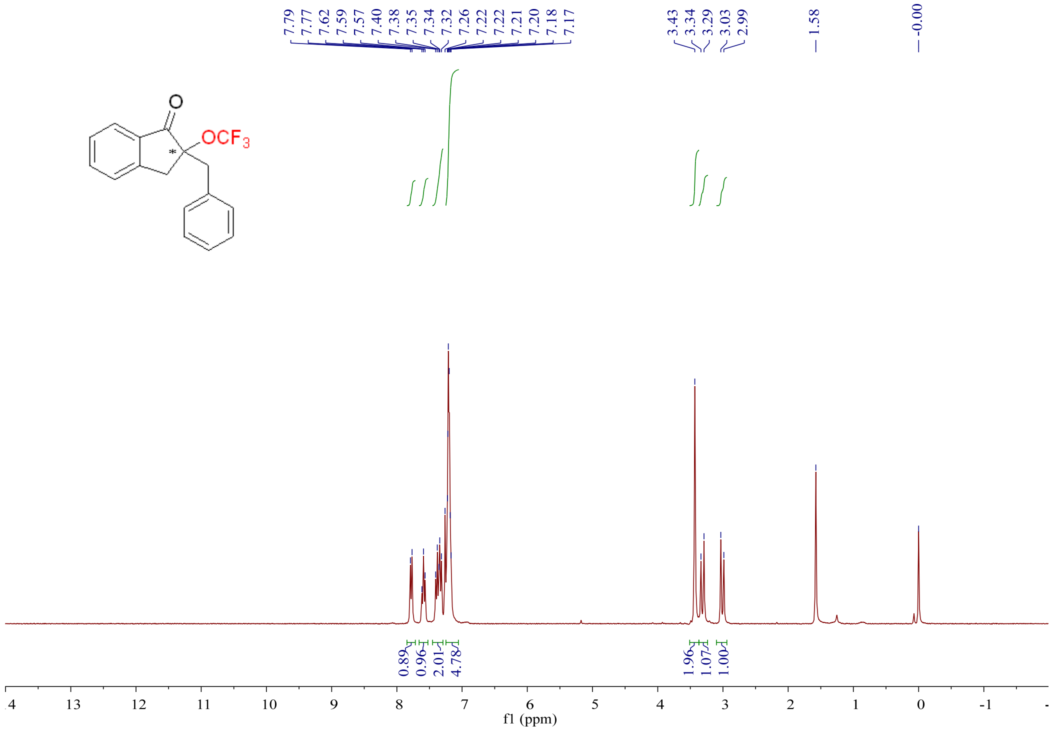

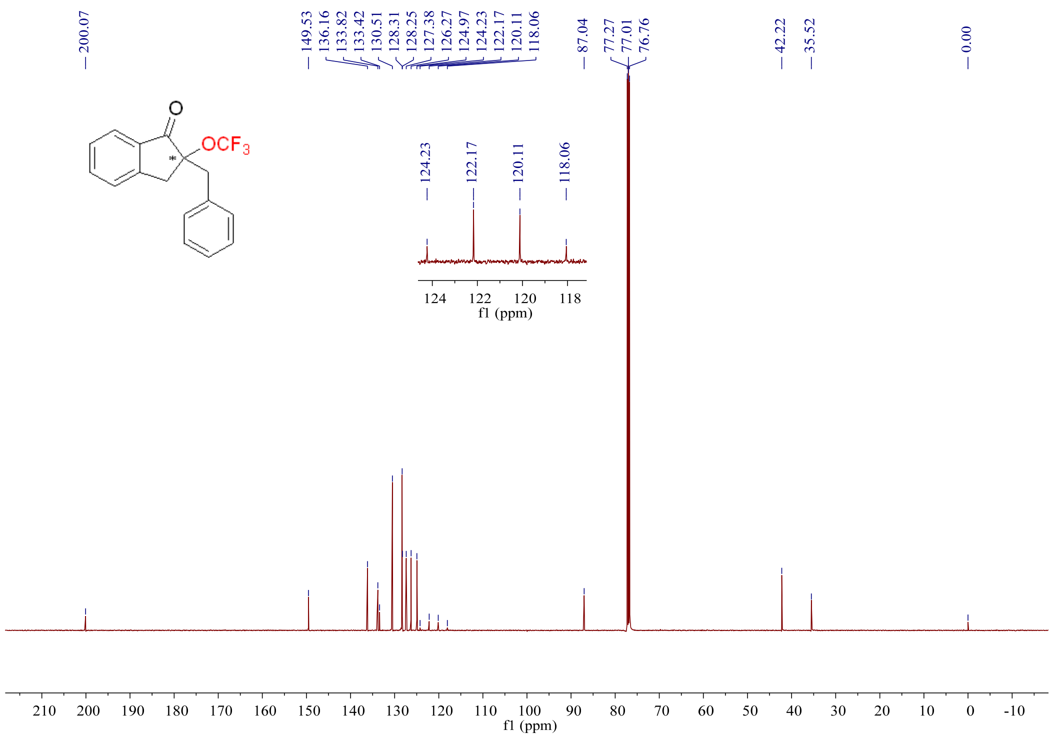

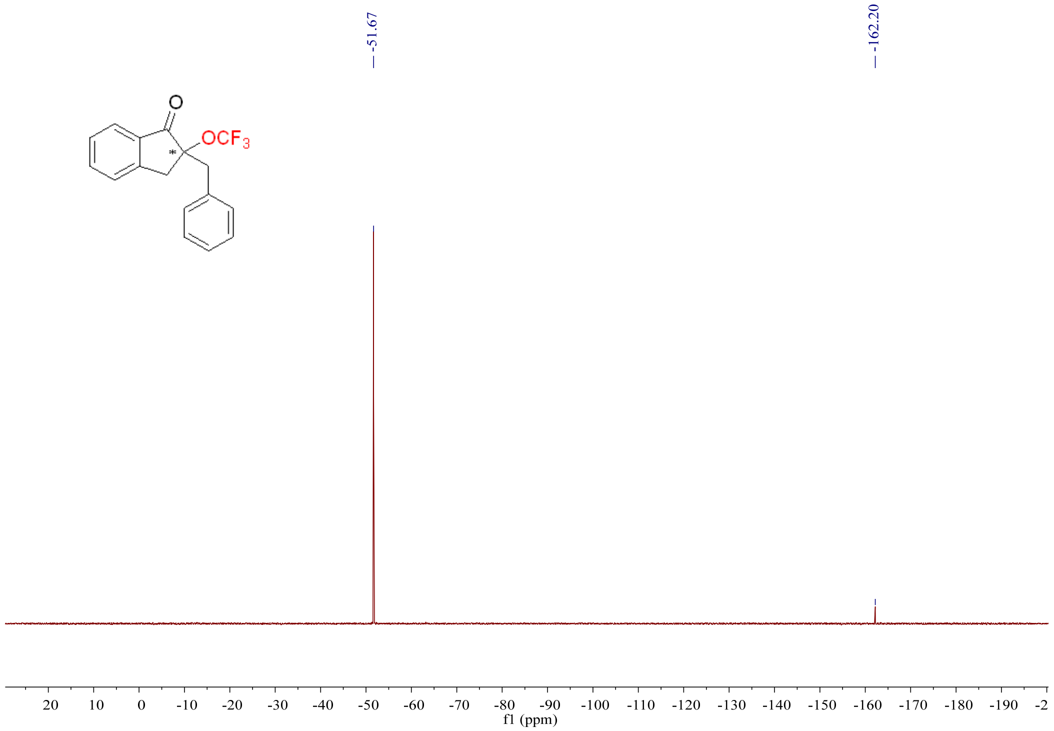

*2-(4-Fluorobenzyl)-2-(trifluoromethoxy)-2,3-dihydro-1H-inden-1-one (**3ab**).*

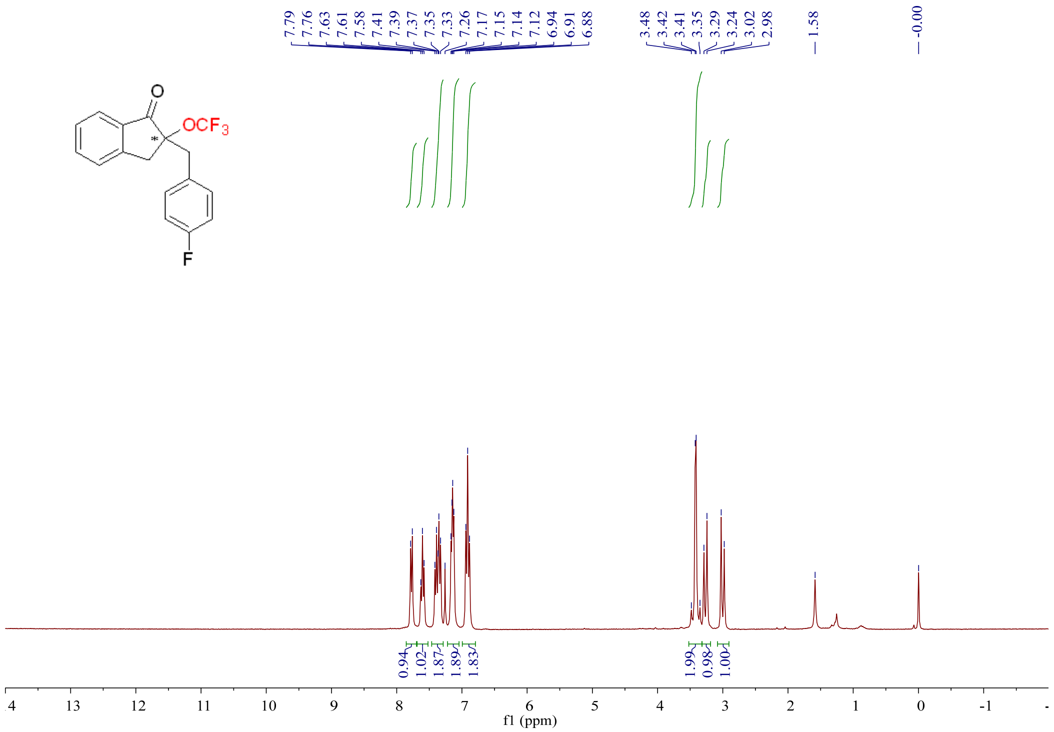

7.79
7.76
7.63
7.61
7.58
7.41
7.39
7.37
7.35
7.33
7.26
7.17
7.15
7.14
7.12
6.94
6.91
6.88
3.48
3.42
3.41
3.35
3.29
3.24
3.02
2.98
1.58
-0.00
O
OCF3
F
0.94
1.02
1.87
1.89
1.83
1.99
0.98
1.00
f1 (ppm)

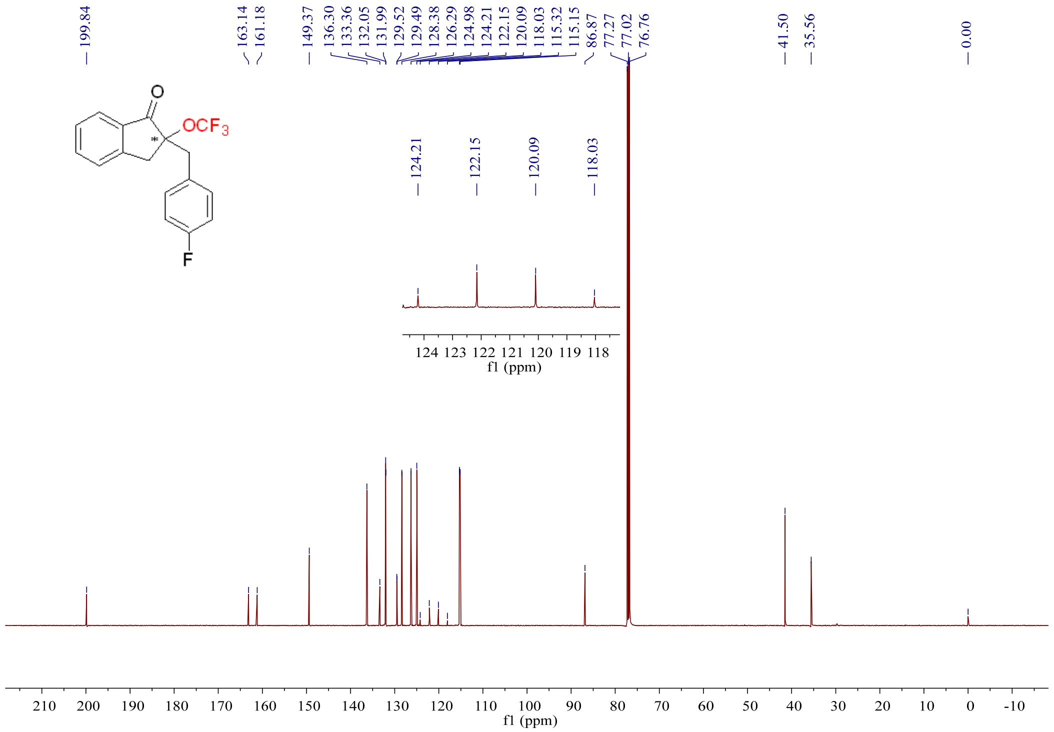

199.84
163.14
161.18
149.37
136.30
133.36
132.05
131.99
129.52
129.49
128.38
126.29
124.98
124.21
122.15
120.09
118.03
115.32
115.15
86.87
77.27
77.02
76.76
41.50
35.56
0.00
O
OCF3
F
124.21
122.15
120.09
118.03
f1 (ppm)
f1 (ppm)

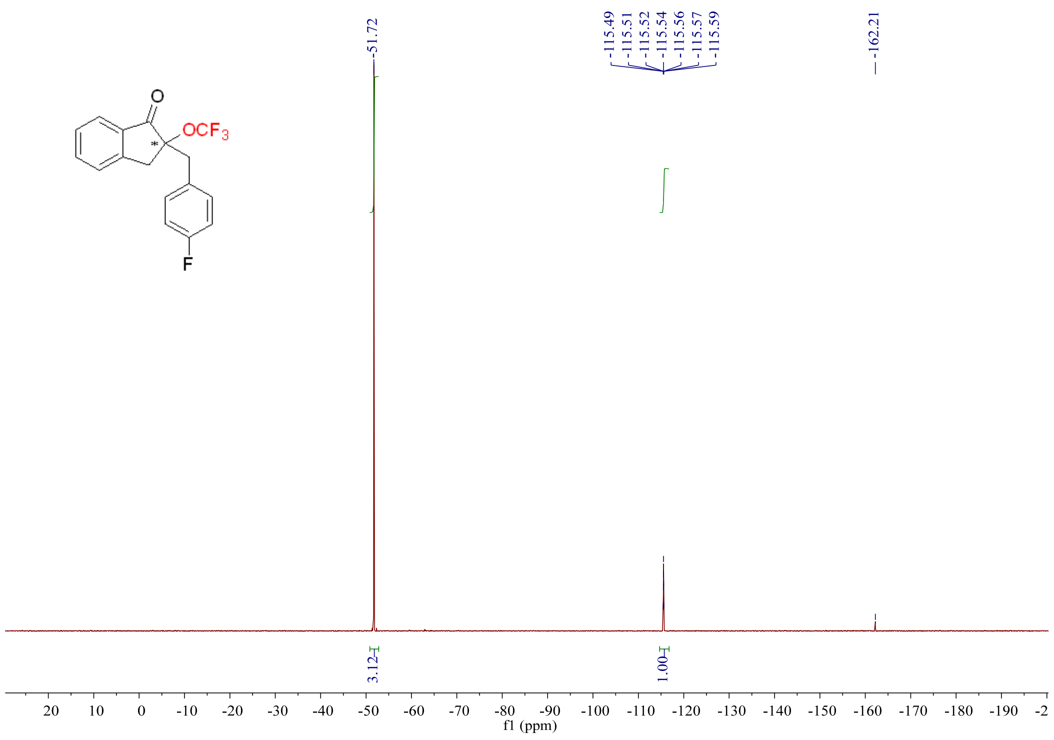

*2-(4-Bromobenzyl)-2-(trifluoromethoxy)-2,3-dihydro-1H-inden-1-one (**3ac**).*

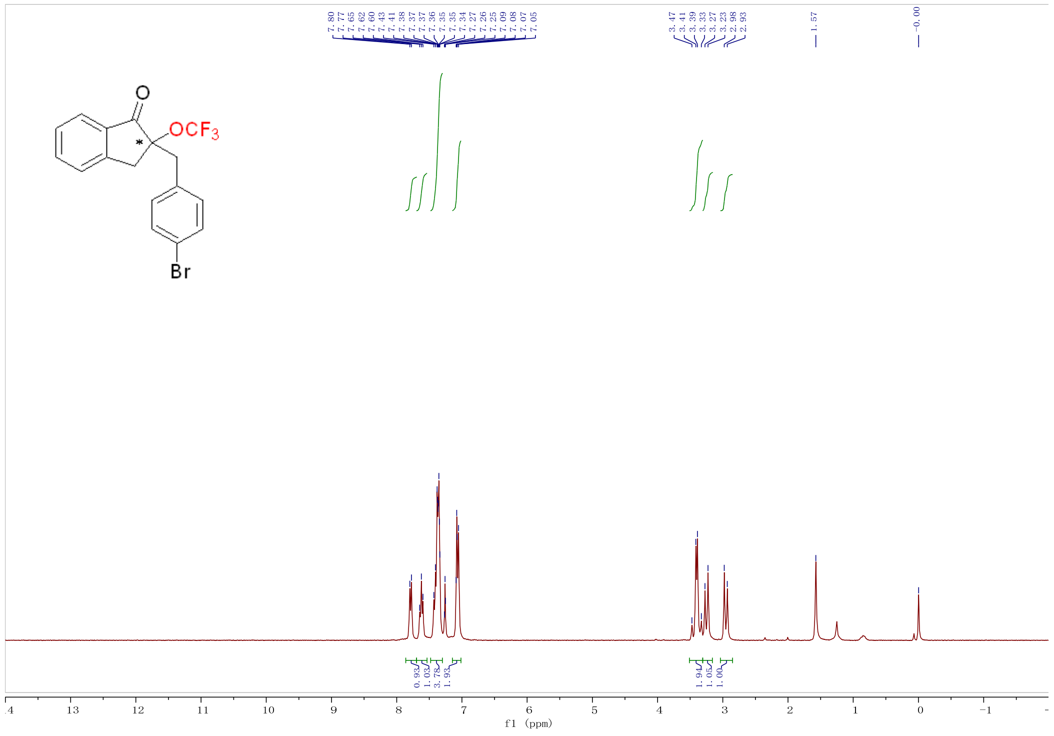

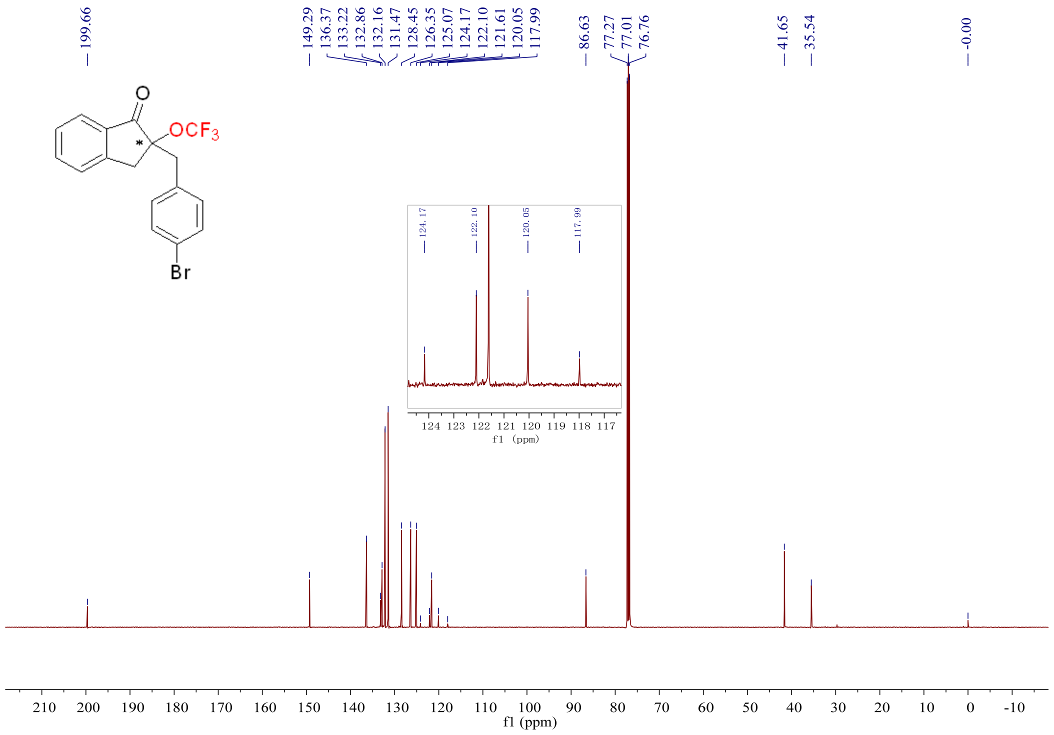
199.66
149.29
136.37
133.22
132.86
132.16
131.47
128.45
126.35
125.07
124.17
122.10
121.61
120.05
117.99
86.63
77.27
77.01
76.76
41.65
35.54
-0.00
O
OCF3
*
Br
124.17
122.10
120.05
117.99
124 123 122 121 120 119 118 117
f1 (ppm)
210 200 190 180 170 160 150 140 130 120 110 100 90 80 70 60 50 40 30 20 10 0 -10
f1 (ppm)

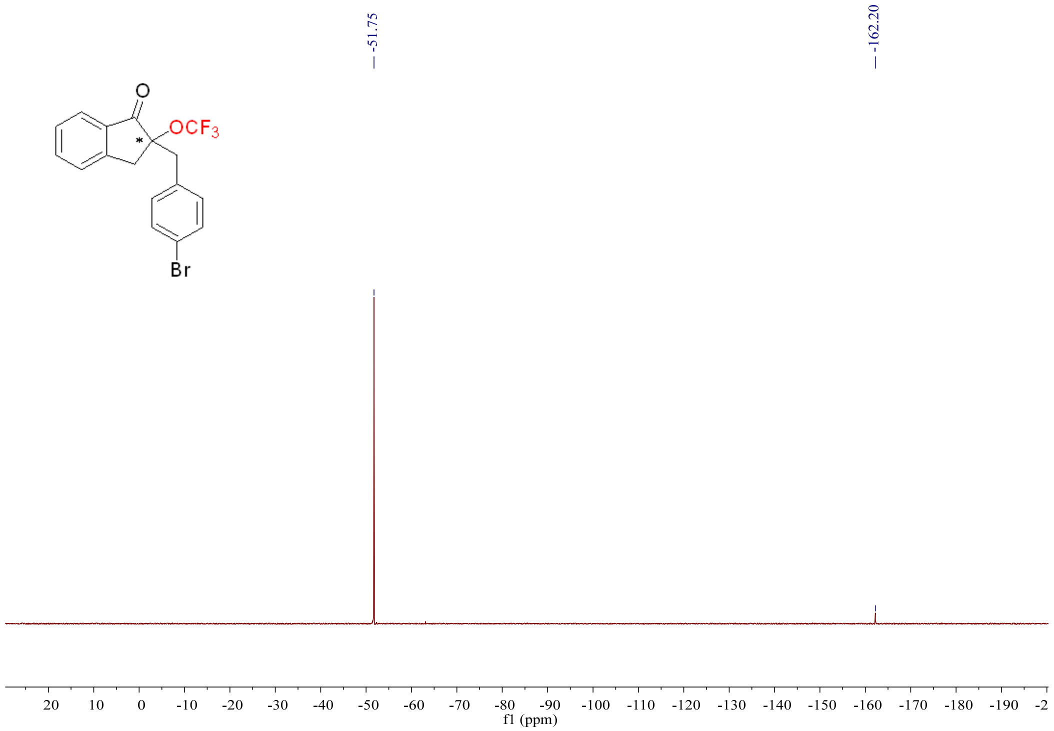
-51.75
-162.20
O
OCF3
*
Br
20 10 0 -10 -20 -30 -40 -50 -60 -70 -80 -90 -100 -110 -120 -130 -140 -150 -160 -170 -180 -190 -2
f1 (ppm)

*2-(Trifluoromethoxy)-2-(4-(trifluoromethyl)benzyl)-2,3-dihydro-1H-inden-1-one (**3ad**).*

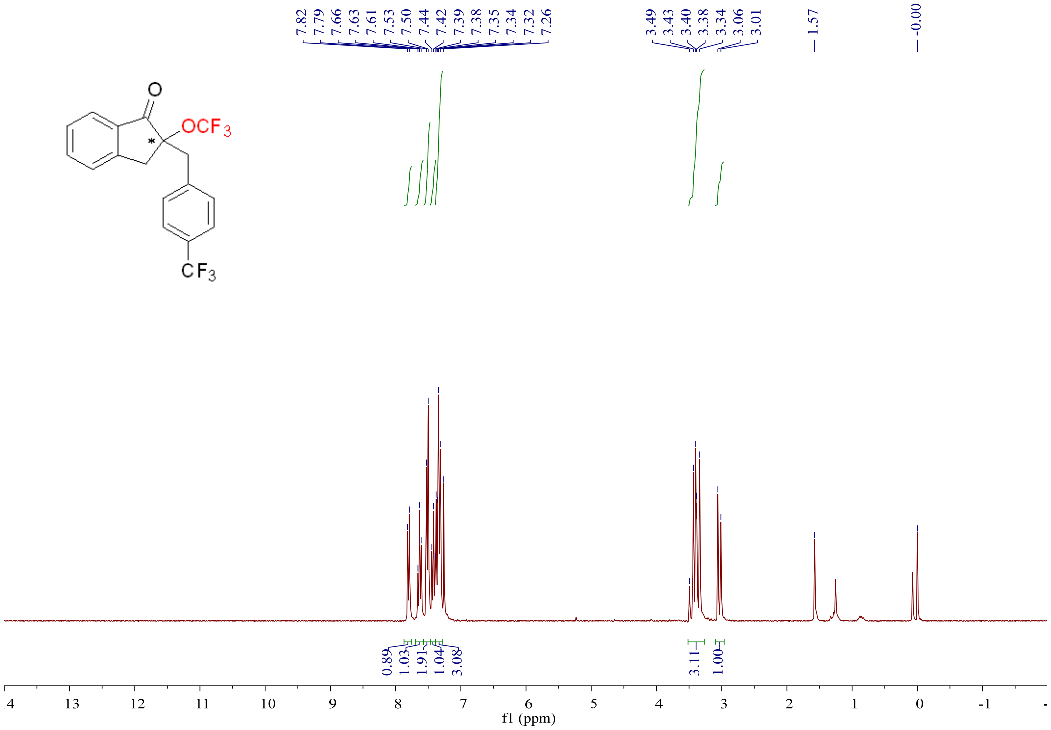

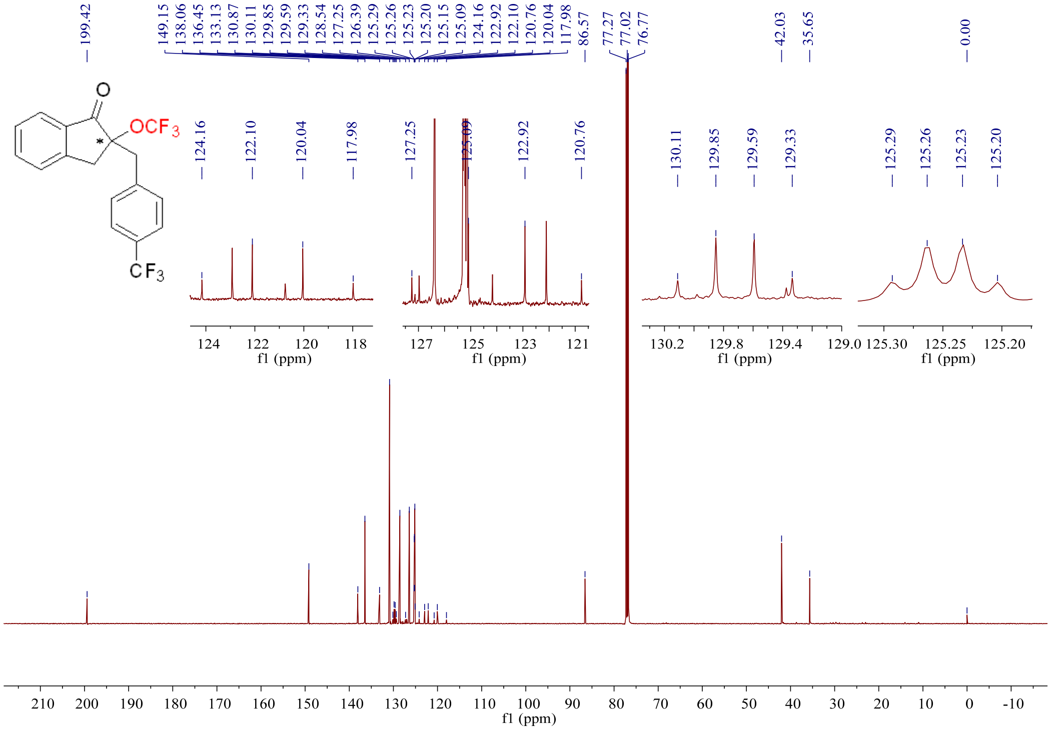

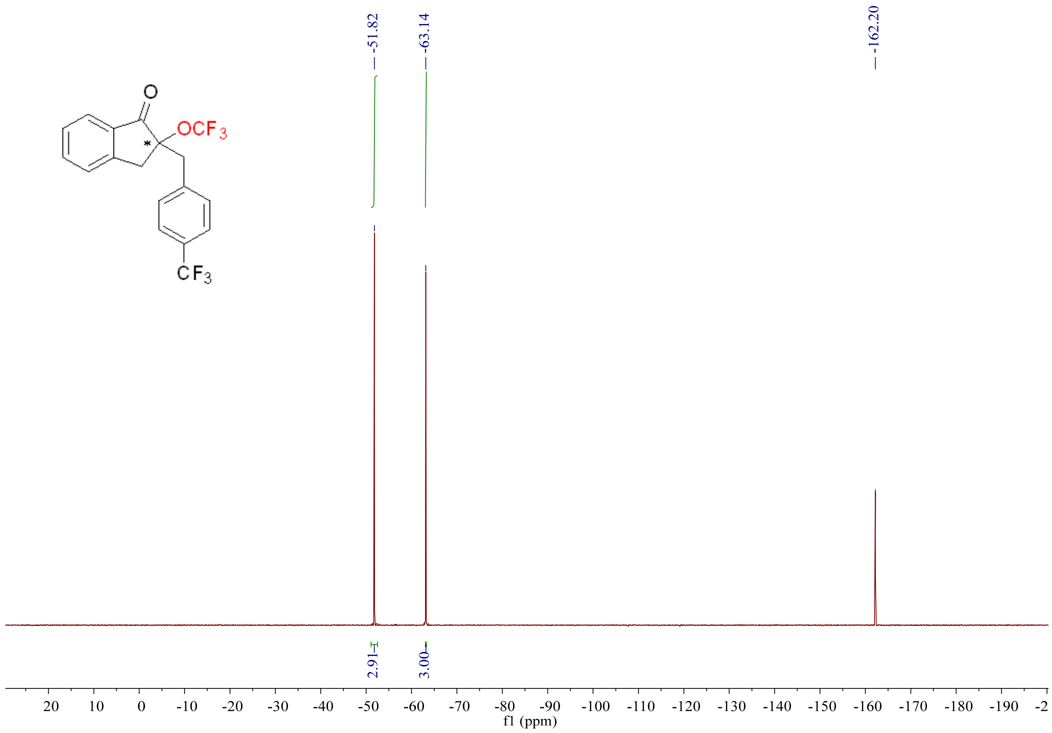

2-(3-Fluorobenzyl)-2-(trifluoromethoxy)-2,3-dihydro-1H-inden-1-one (**3ae**).

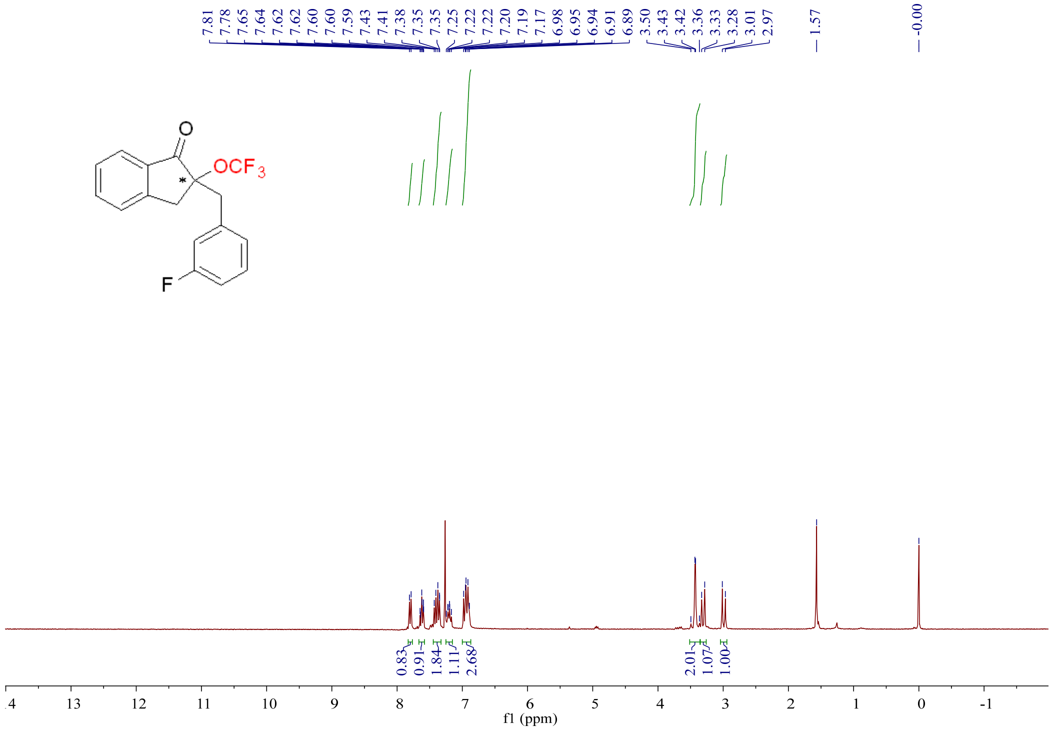

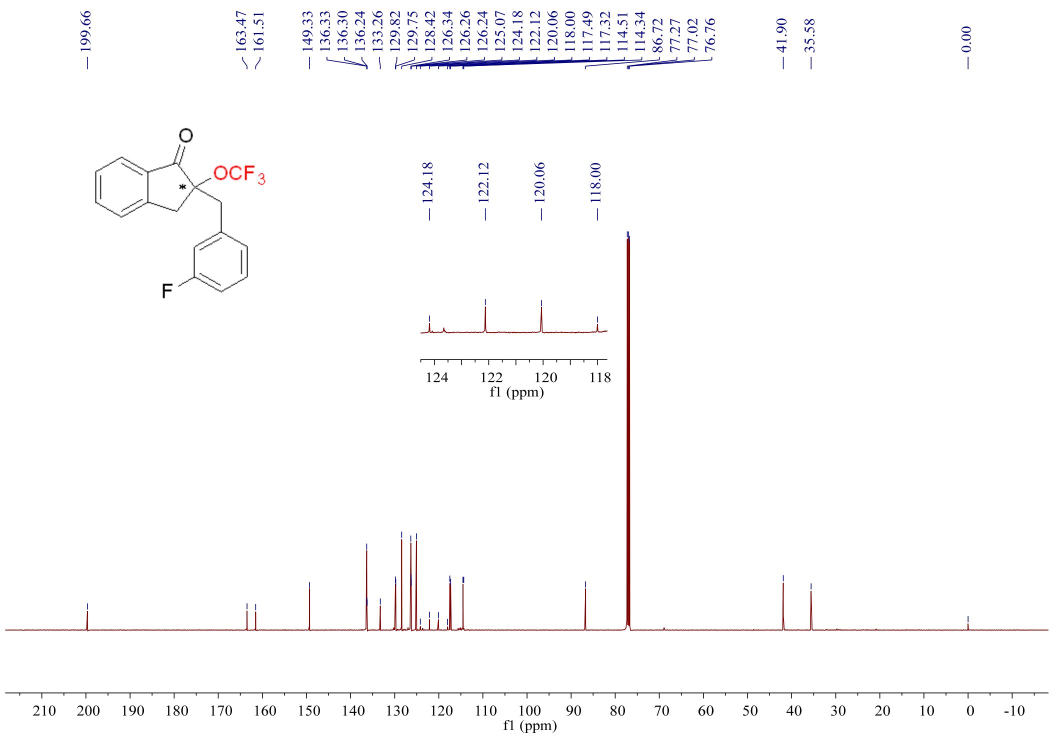

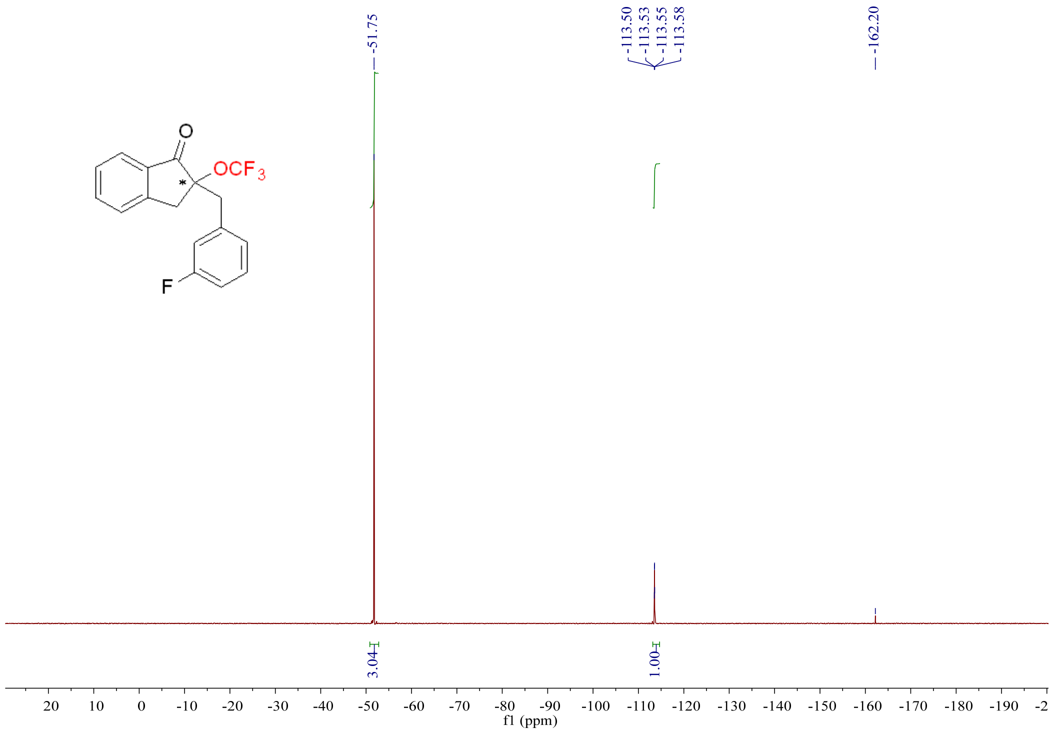

*2-([1,1'-Biphenyl]-4-ylmethyl)-2-(trifluoromethoxy)-2,3-dihydro-1H-inden-1-one (**3af**).*

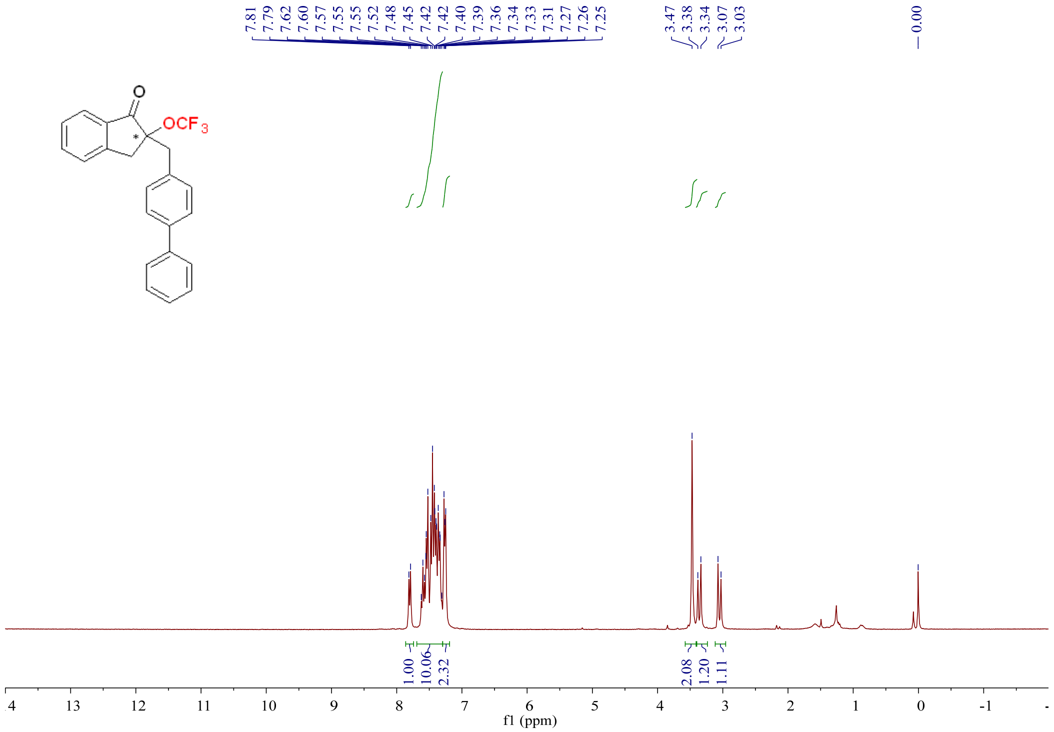
OCF3
*
7.81 7.79 7.62 7.60 7.57 7.55 7.55 7.52 7.48 7.45 7.42 7.42 7.40 7.39 7.36 7.34 7.33 7.31 7.27 7.26 7.25
3.47 3.38 3.34 3.07 3.03
0.00
1.00 10.06 2.32
2.08 1.20 1.11
f1 (ppm)

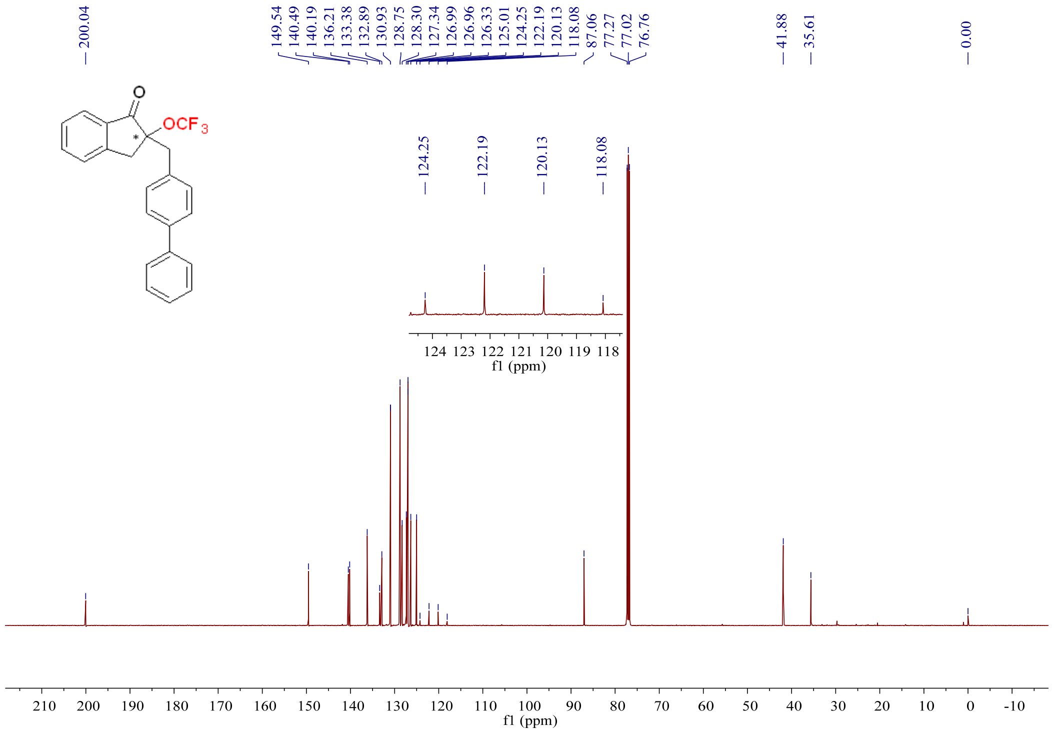
OCF3
*
200.04
149.54 140.49 140.19 136.21 133.38 132.89 130.93 128.75 128.30 127.34 126.99 126.96 126.33 125.01 124.25 122.19 120.13 118.08 87.06 77.27 77.02 76.76
41.88 35.61
0.00
124.25 122.19 120.13 118.08
f1 (ppm)

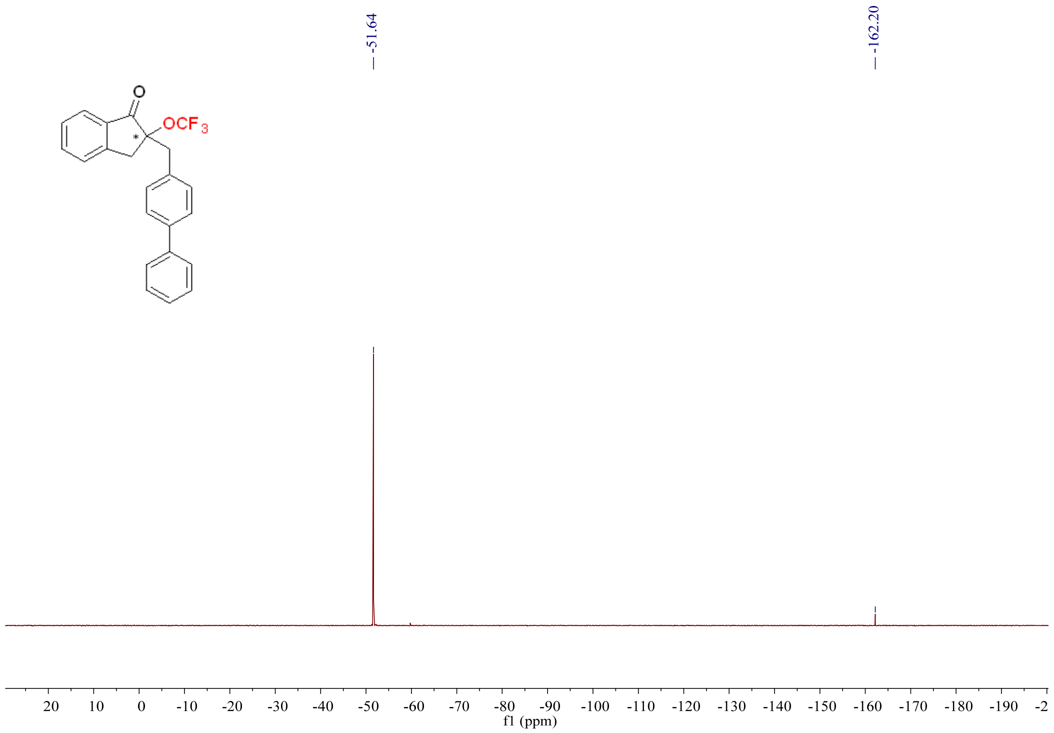

*2-(Naphthalen-2-ylmethyl)-2-(trifluoromethoxy)-2,3-dihydro-1H-inden-1-one (**3ag**).*

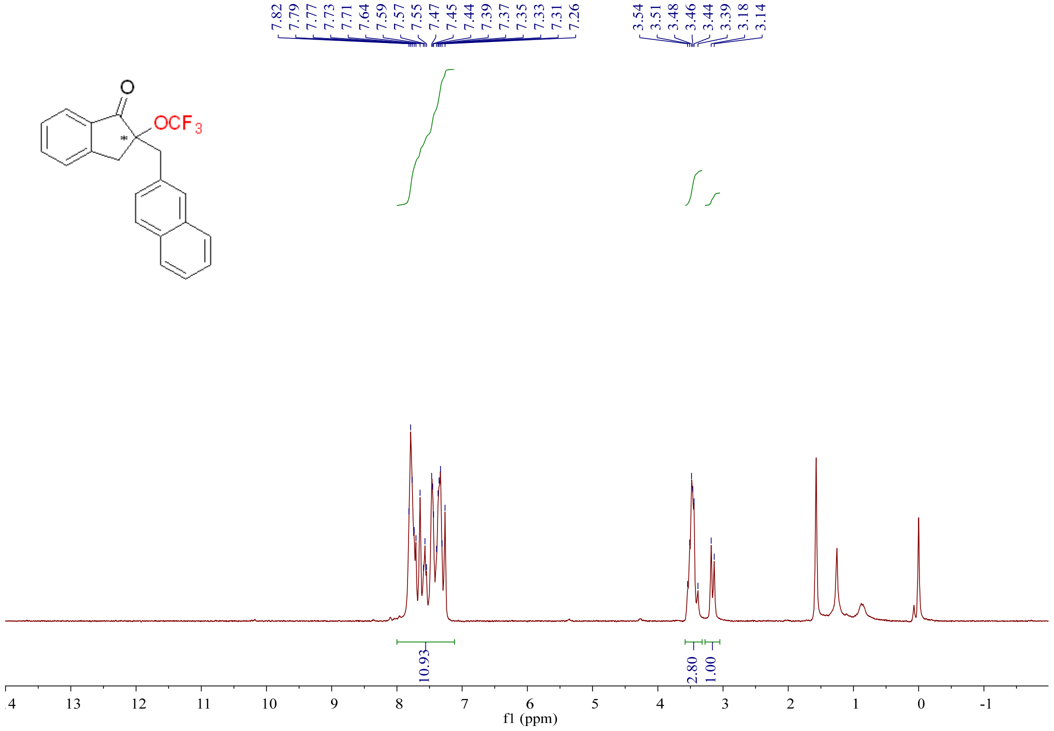

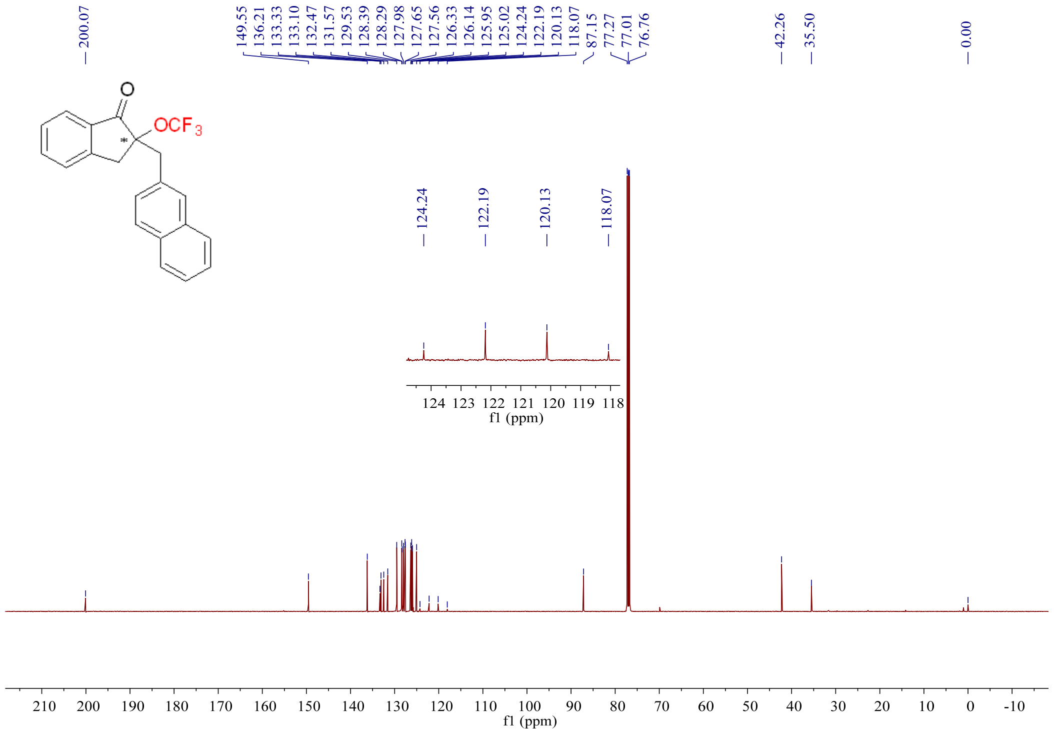

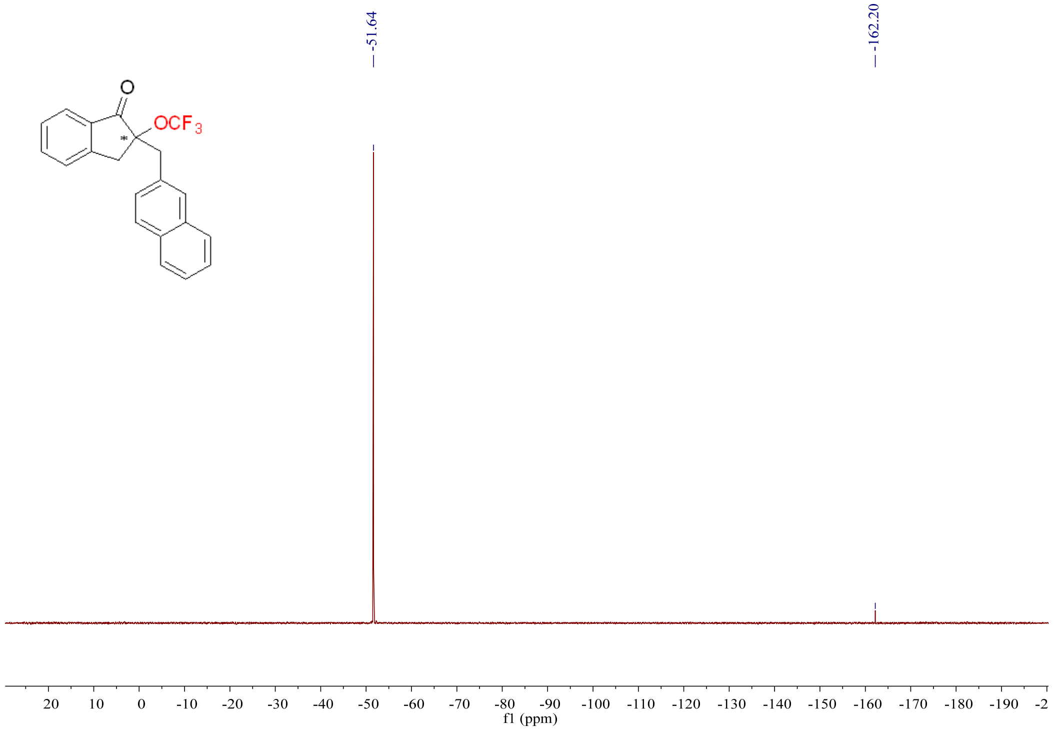

*2-(3,5-di-tert-butylbenzyl)-2-(trifluoromethoxy)-2,3-dihydro-1H-inden-1-one (**3ah**).*

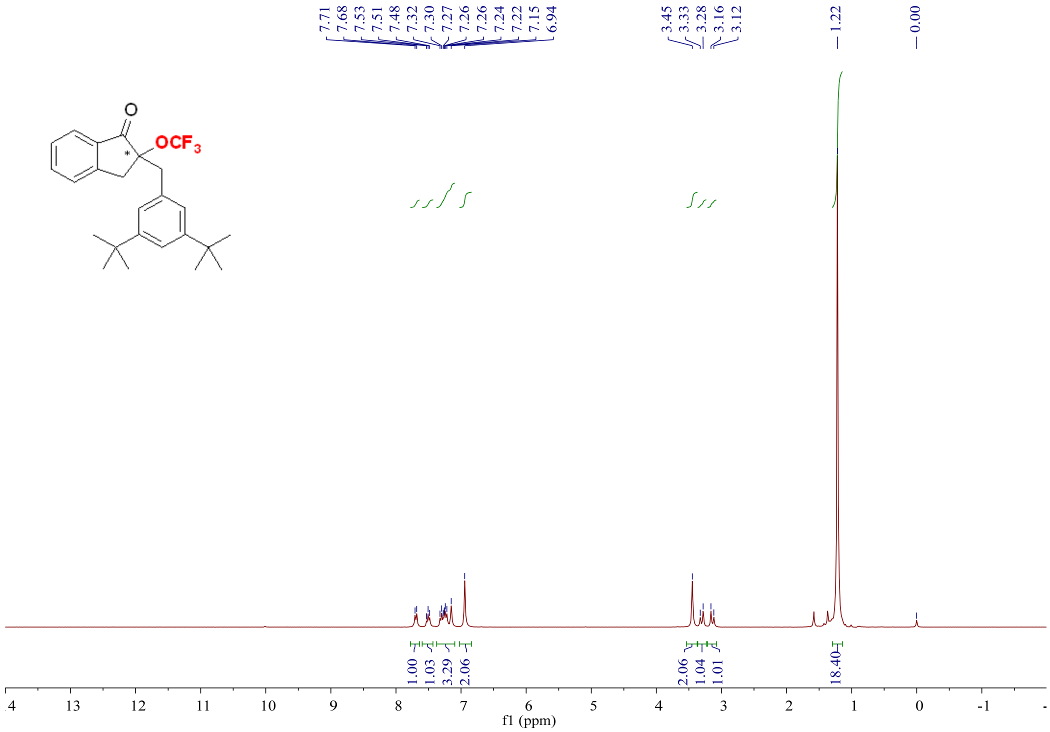
7.71
7.68
7.53
7.51
7.48
7.32
7.30
7.27
7.26
7.26
7.24
7.22
7.15
6.94
3.45
3.33
3.28
3.16
3.12
1.22
0.00
OCF3
1.00
1.03
3.29
2.06
2.06
1.04
1.01
18.40
f1 (ppm)

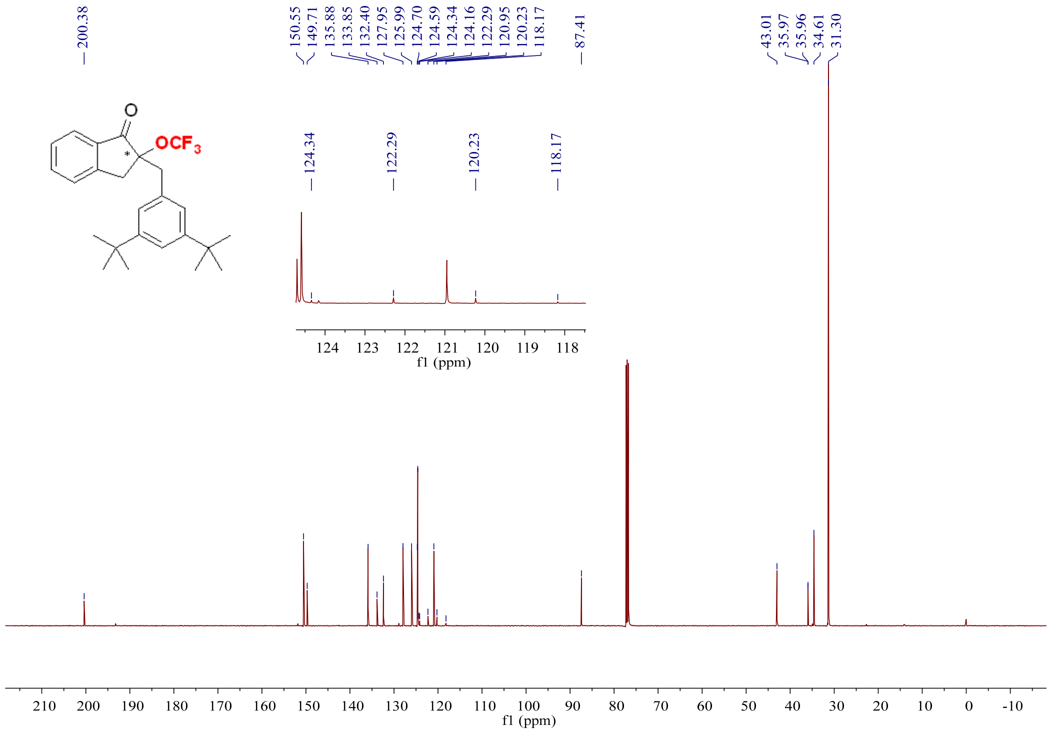
200.38
150.55
149.71
135.88
133.85
132.40
127.95
125.99
124.70
124.59
124.34
124.16
122.29
120.95
120.23
118.17
87.41
43.01
35.97
35.96
34.61
31.30
OCF3
124.34
122.29
120.23
118.17
f1 (ppm)
f1 (ppm)

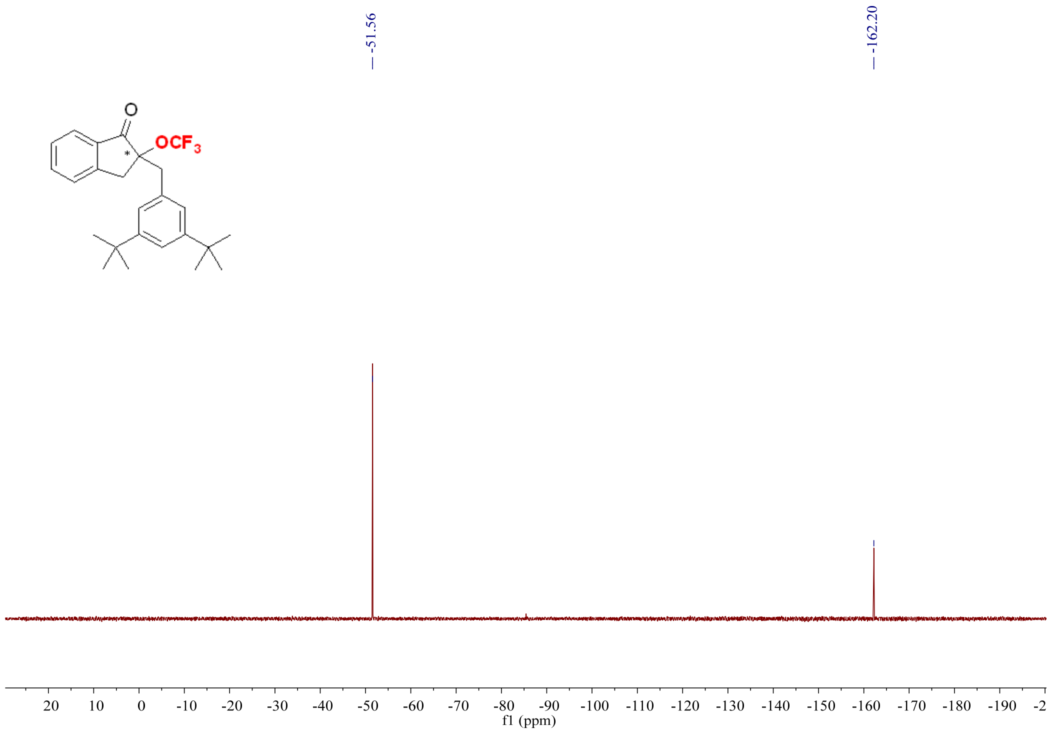

*2-Benzyl-5-bromo-2-(trifluoromethoxy)-2,3-dihydro-1H-inden-1-one (**3ba**).*

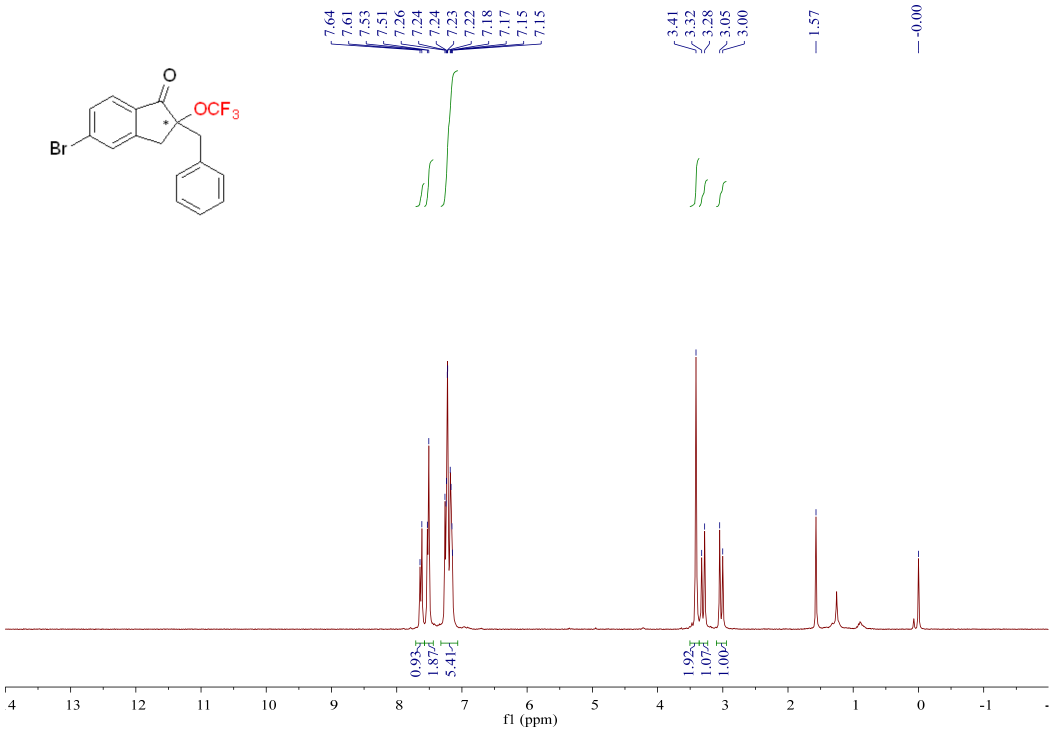

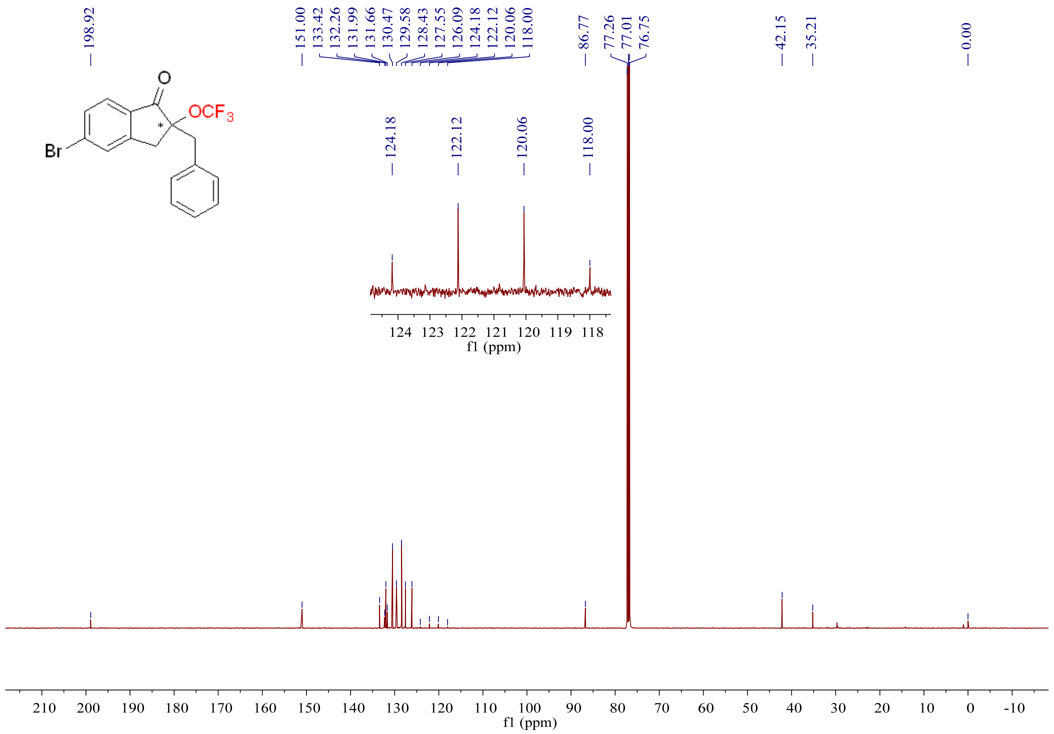

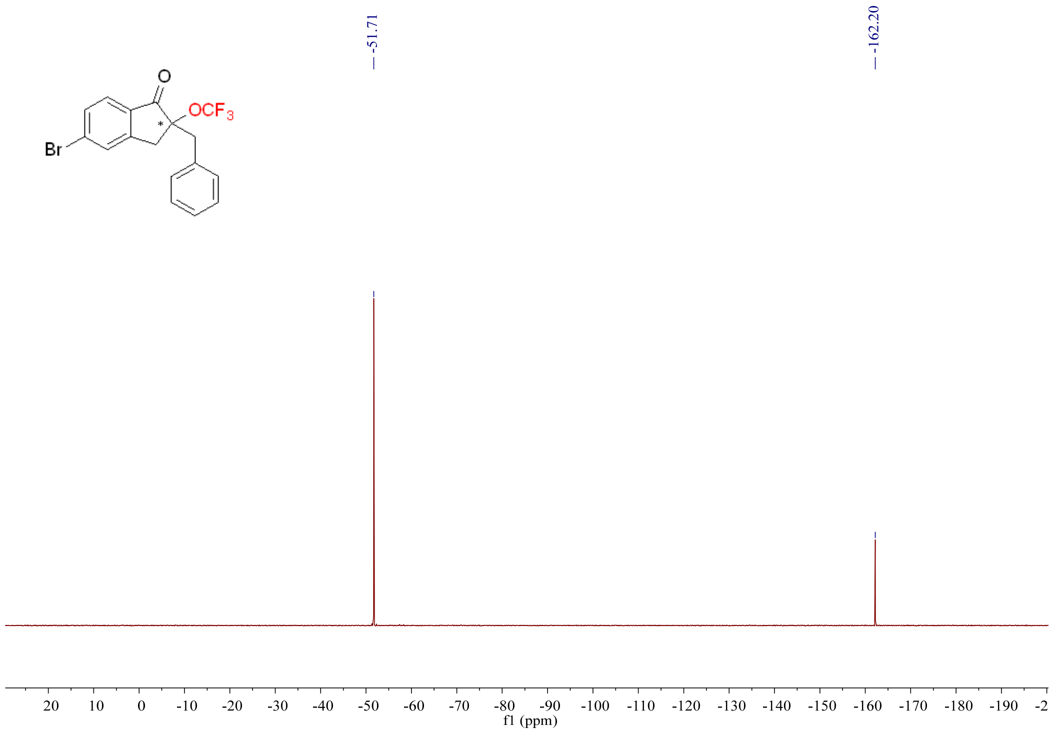

*2-Benzyl-5-fluoro-2-(trifluoromethoxy)-2,3-dihydro-1H-inden-1-one (**3ca**).*

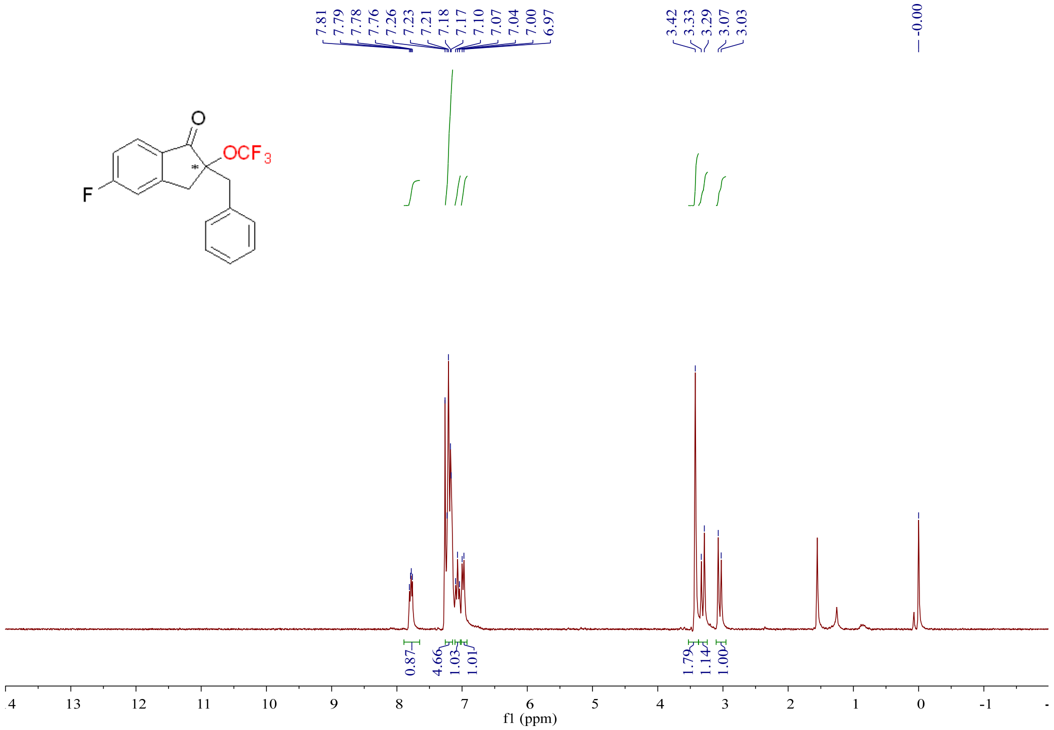
7.81 7.79 7.78 7.76 7.26 7.23 7.21 7.18 7.17 7.10 7.07 7.04 7.00 6.97
3.42 3.33 3.29 3.07 3.03
-0.00
O
OCF3
F
0.87
4.66
1.03
1.01
1.79
1.14
1.00
f1 (ppm)

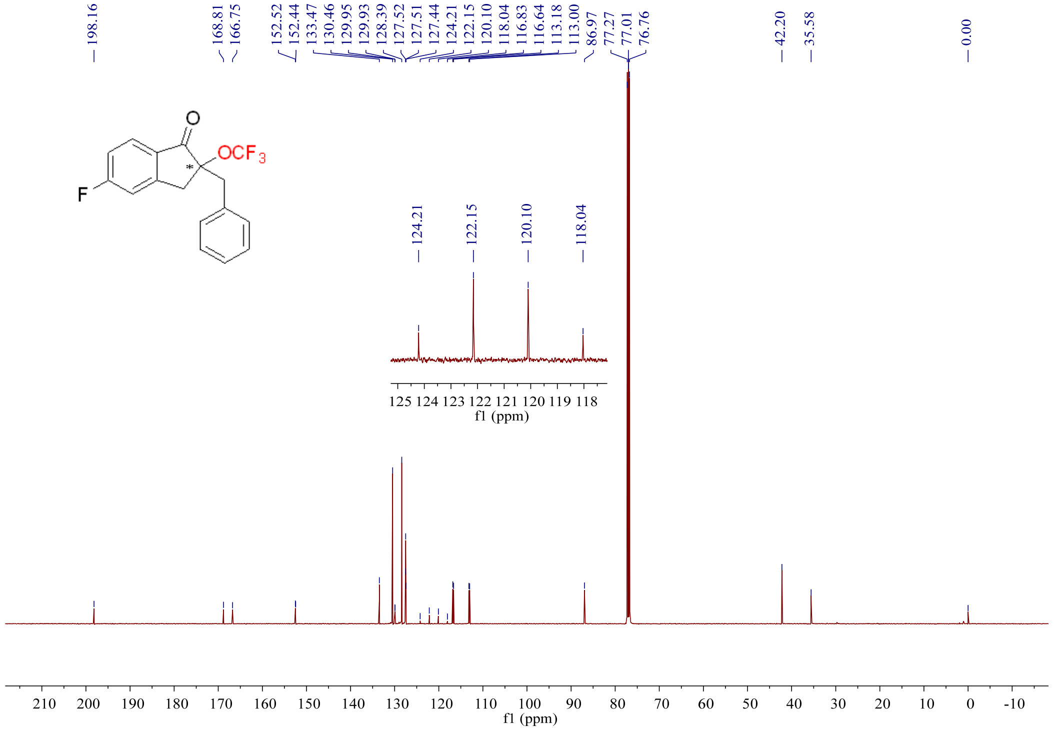
198.16
168.81 166.75
152.52 152.44 133.47 130.46 129.95 129.93 128.39 127.52 127.51 127.44 124.21 122.15 120.10 118.04 116.83 116.64 113.18 113.00
86.97 77.27 77.01 76.76
42.20
35.58
0.00
O
OCF3
F
124.21
122.15
120.10
118.04
f1 (ppm)
f1 (ppm)

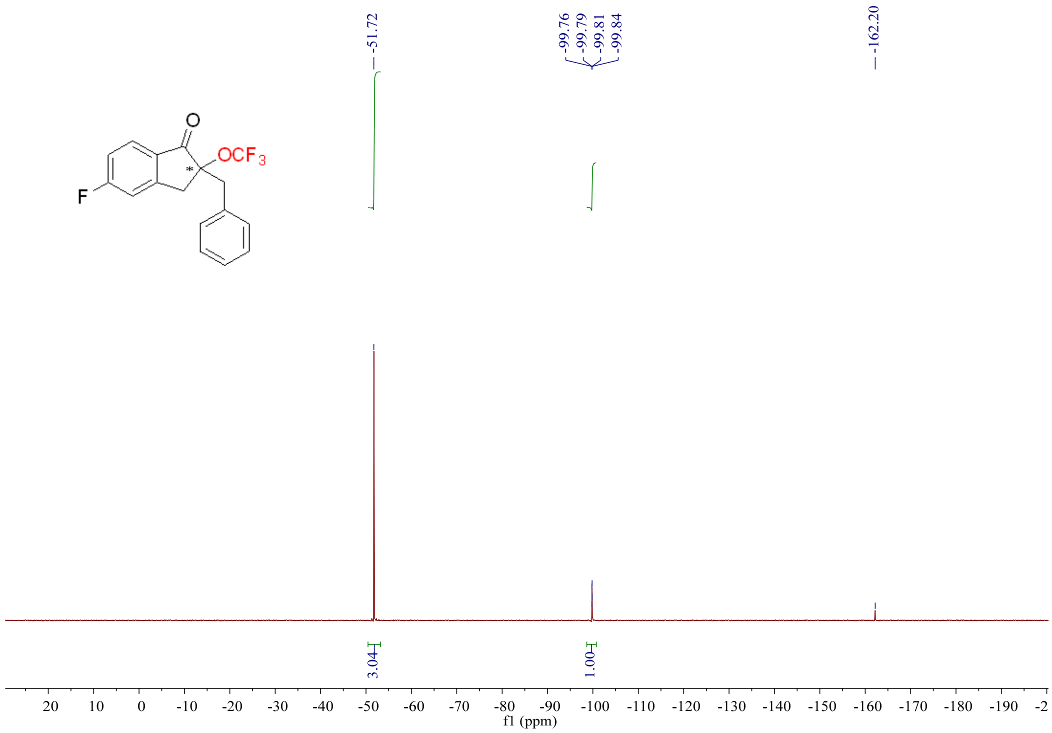

*2-Benzyl-6-fluoro-2-(trifluoromethoxy)-2,3-dihydro-1H-inden-1-one (**3da**).*

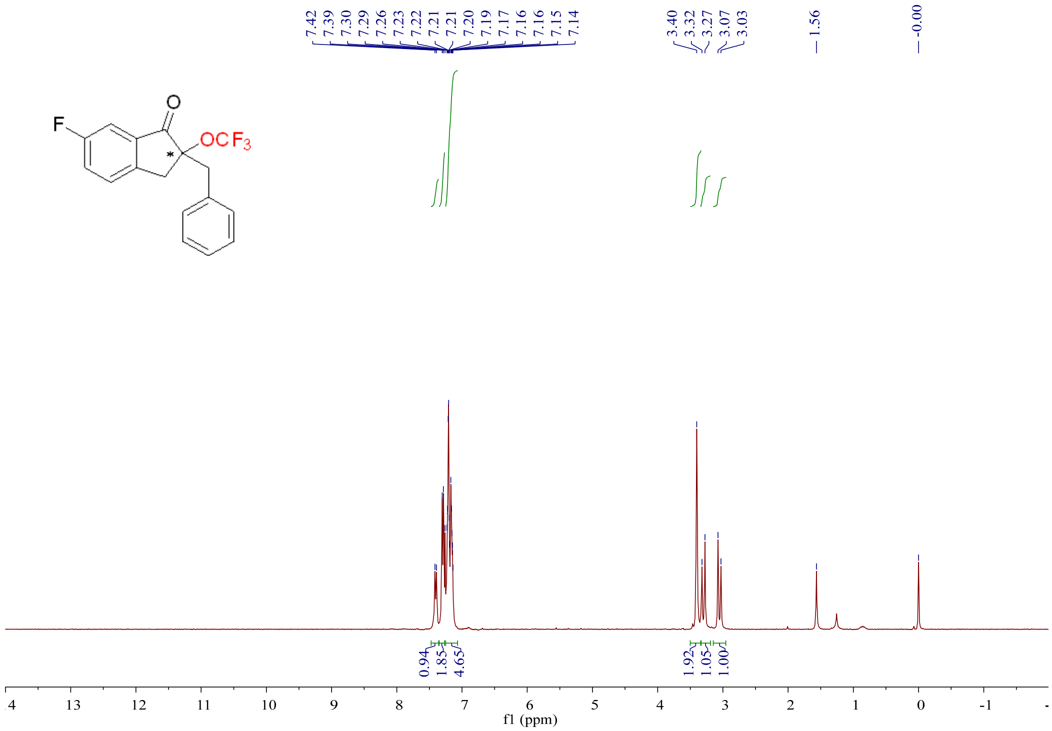

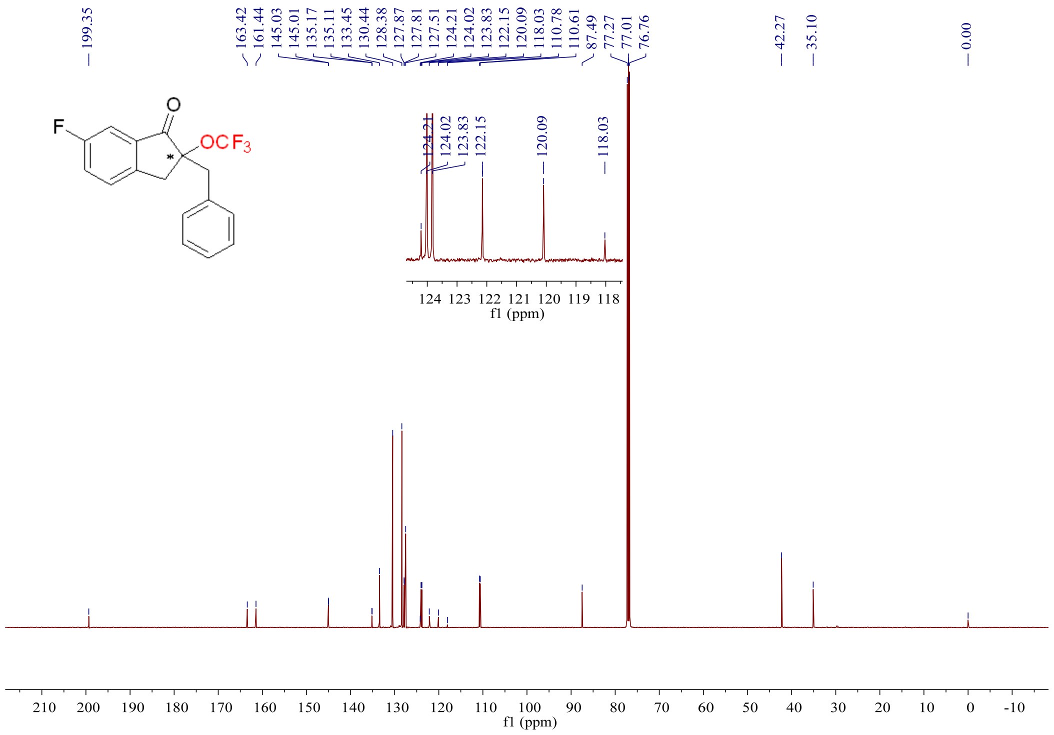

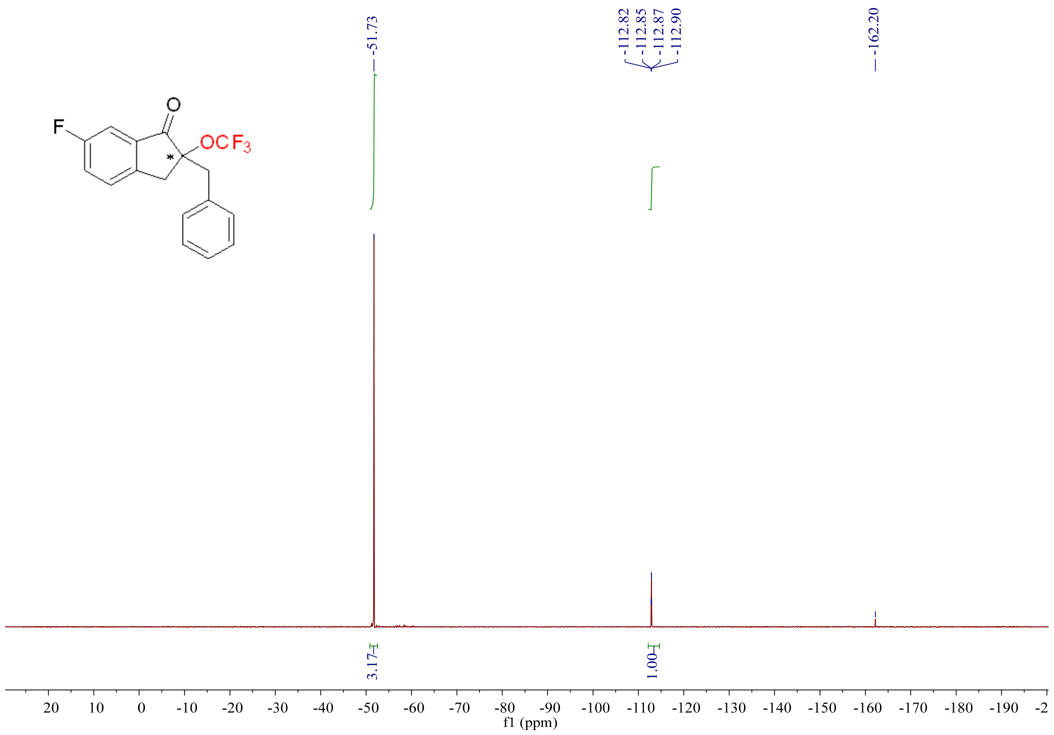

*2-Benzyl-6-methyl-2-(trifluoromethoxy)-2,3-dihydro-1H-inden-1-one (**3ea**).*

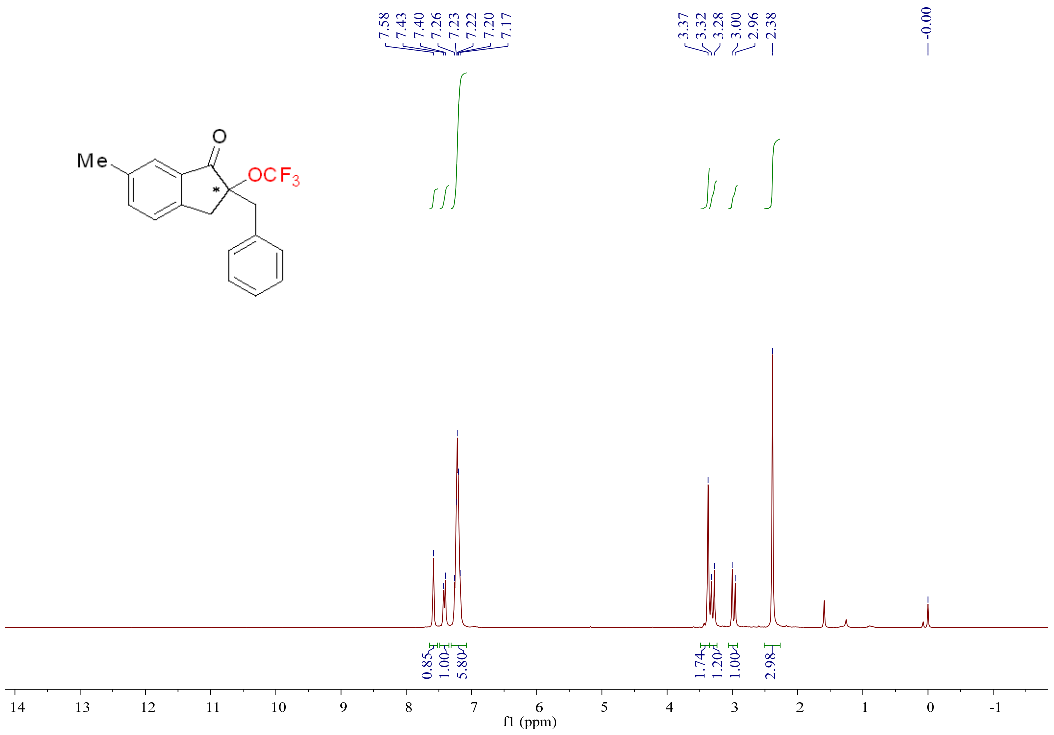

Me
O
OCF3
*
7.58
7.43
7.40
7.26
7.23
7.22
7.20
7.17
3.37
3.32
3.28
3.00
2.96
2.38
-0.00
0.85
1.00
5.80
1.74
1.20
1.00
2.98
14 13 12 11 10 9 8 7 6 5 4 3 2 1 0 -1
f1 (ppm)

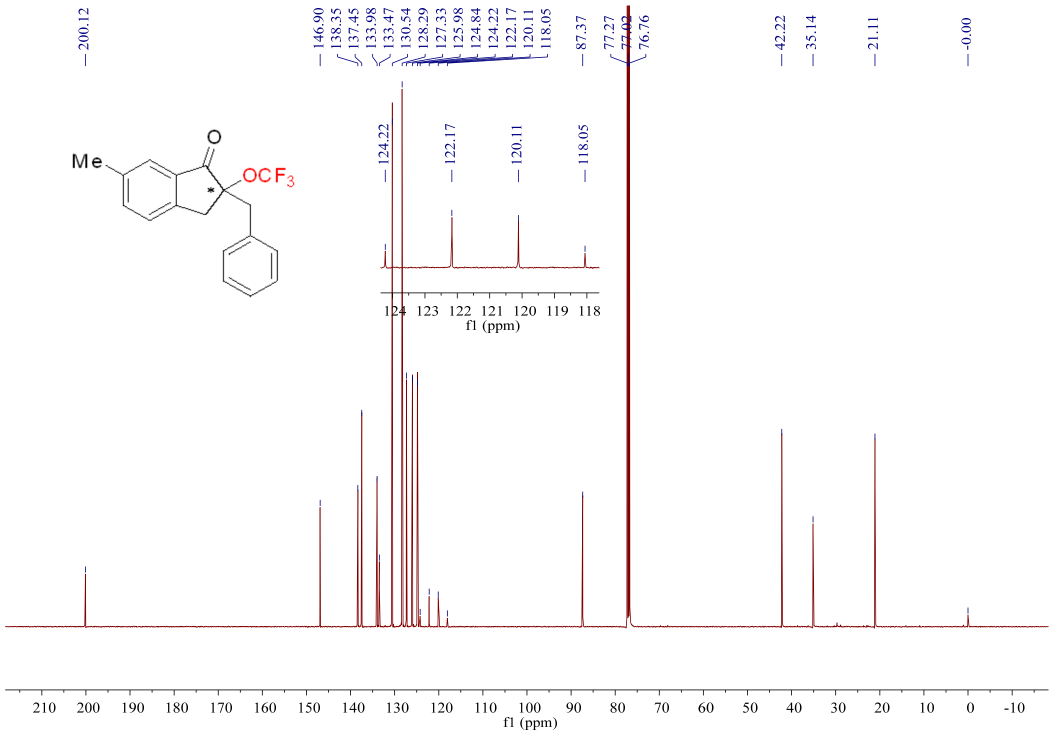

Me
O
OCF3
*
200.12
146.90
138.35
137.45
133.98
133.47
130.54
128.29
127.33
125.98
124.84
124.22
122.17
120.11
118.05
87.37
77.27
77.02
76.76
42.22
35.14
21.11
-0.00
124.22
122.17
120.11
118.05
124 123 122 121 120 119 118
f1 (ppm)
210 200 190 180 170 160 150 140 130 120 110 100 90 80 70 60 50 40 30 20 10 0 -10
f1 (ppm)

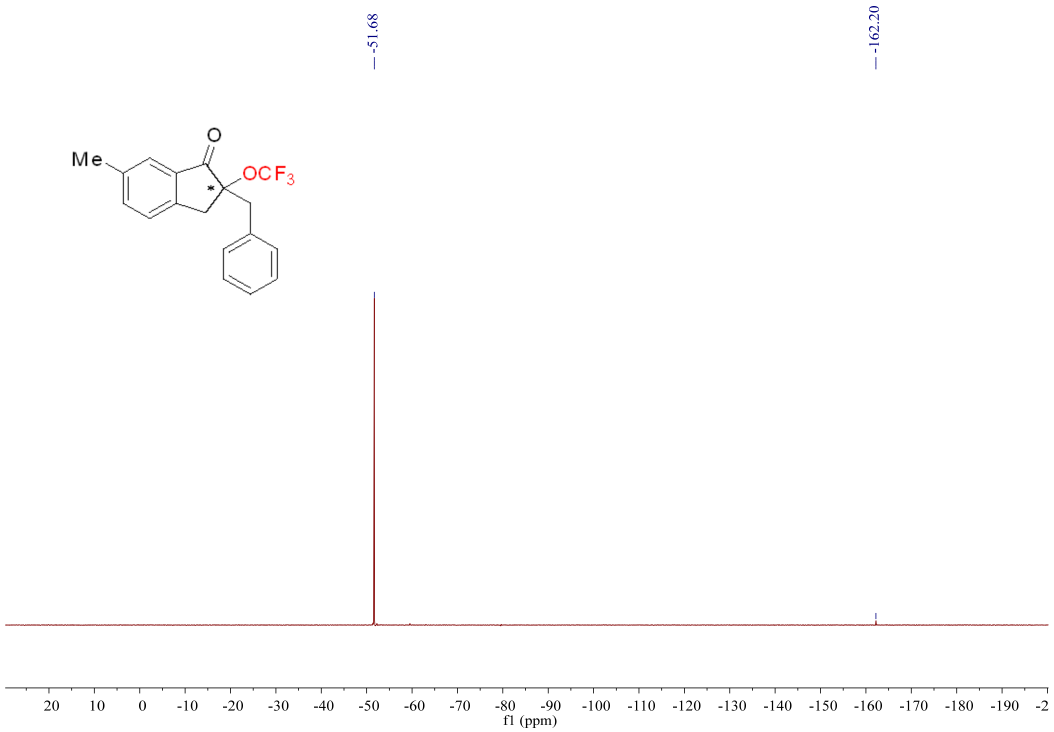

*2-Benzyl-6-methoxy-2-(trifluoromethoxy)-2,3-dihydro-1H-inden-1-one (**3fa**)*.

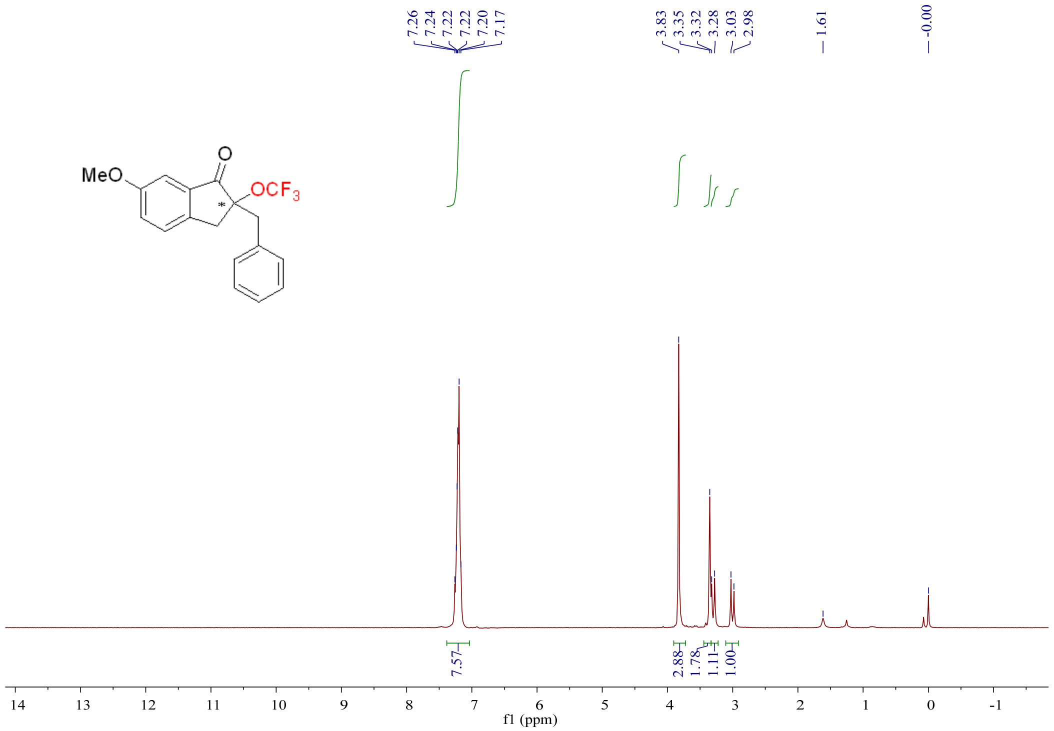

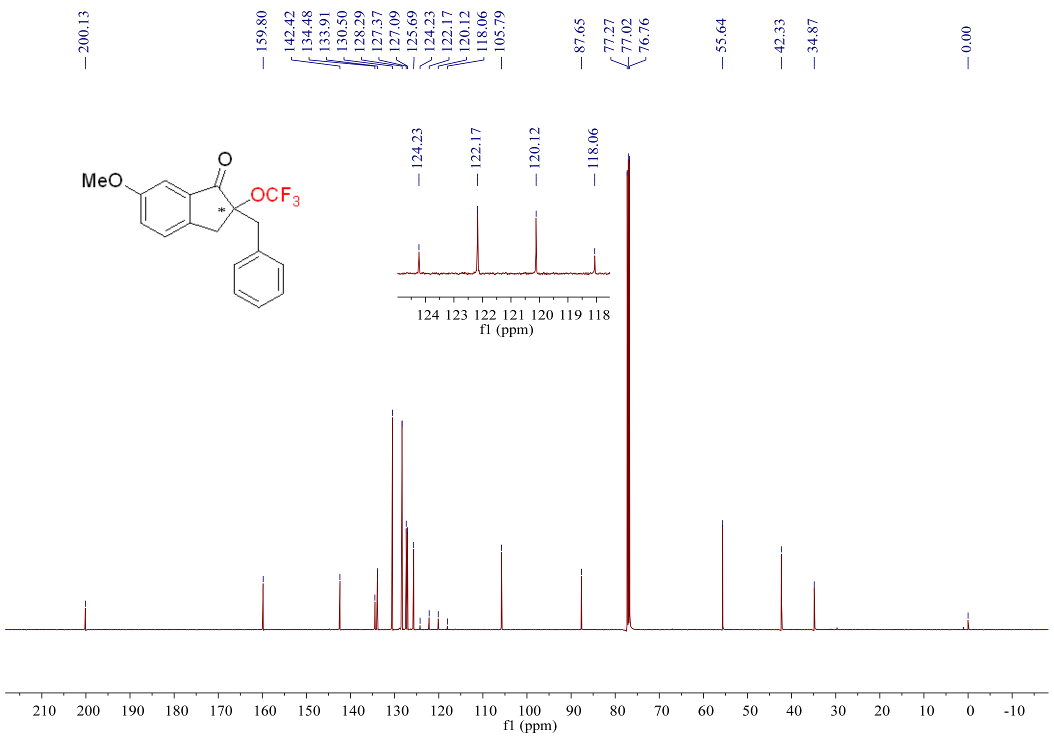

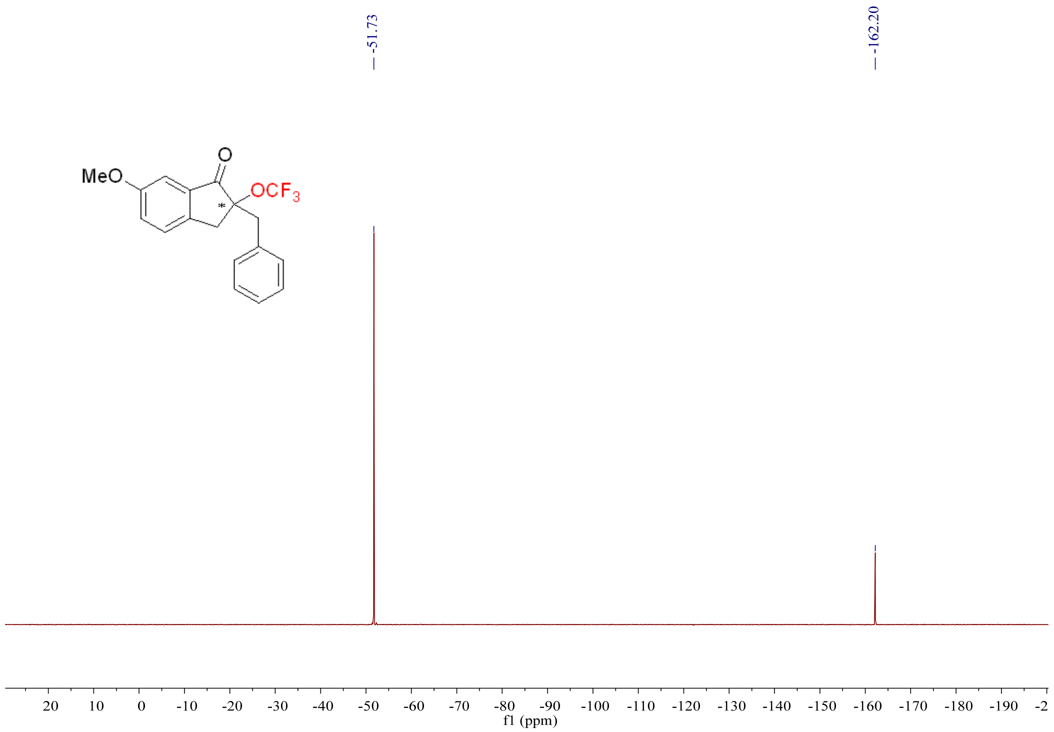

*2-Benzyl-5,6-dimethoxy-2-(trifluoromethoxy)-2,3-dihydro-1H-inden-1-one (**3ga**).*

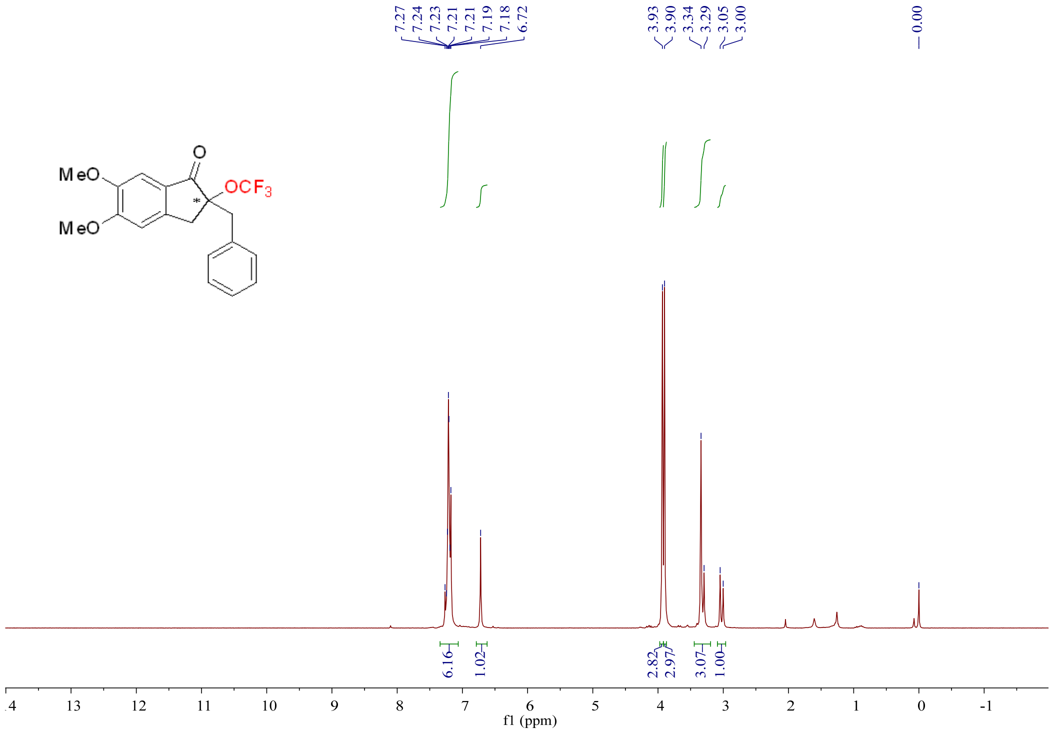

MeO
MeO
O
OCF3
*
7.27
7.24
7.23
7.21
7.21
7.19
7.18
6.72
3.93
3.90
3.34
3.29
3.05
3.00
0.00
6.16
1.02
2.82
2.97
3.07
1.00
f1 (ppm)

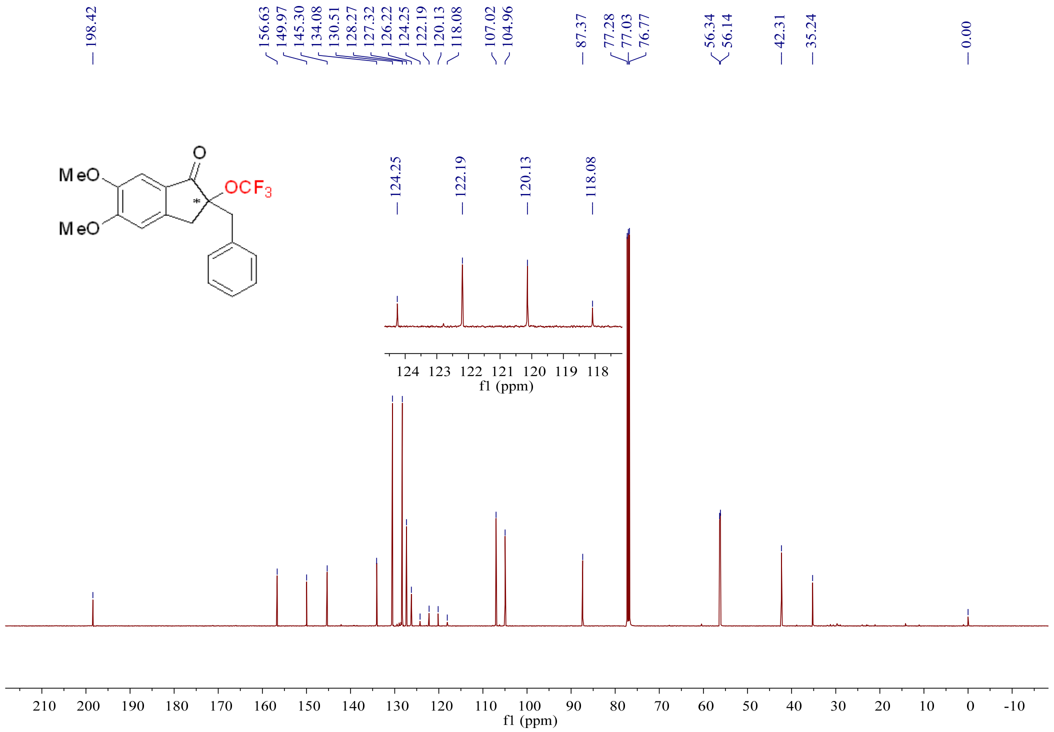

MeO
MeO
O
OCF3
*
198.42
156.63
149.97
145.30
134.08
130.51
128.27
127.32
126.22
124.25
122.19
120.13
118.08
107.02
104.96
87.37
77.28
77.03
76.77
56.34
56.14
42.31
35.24
0.00
124.25
122.19
120.13
118.08
f1 (ppm)
f1 (ppm)

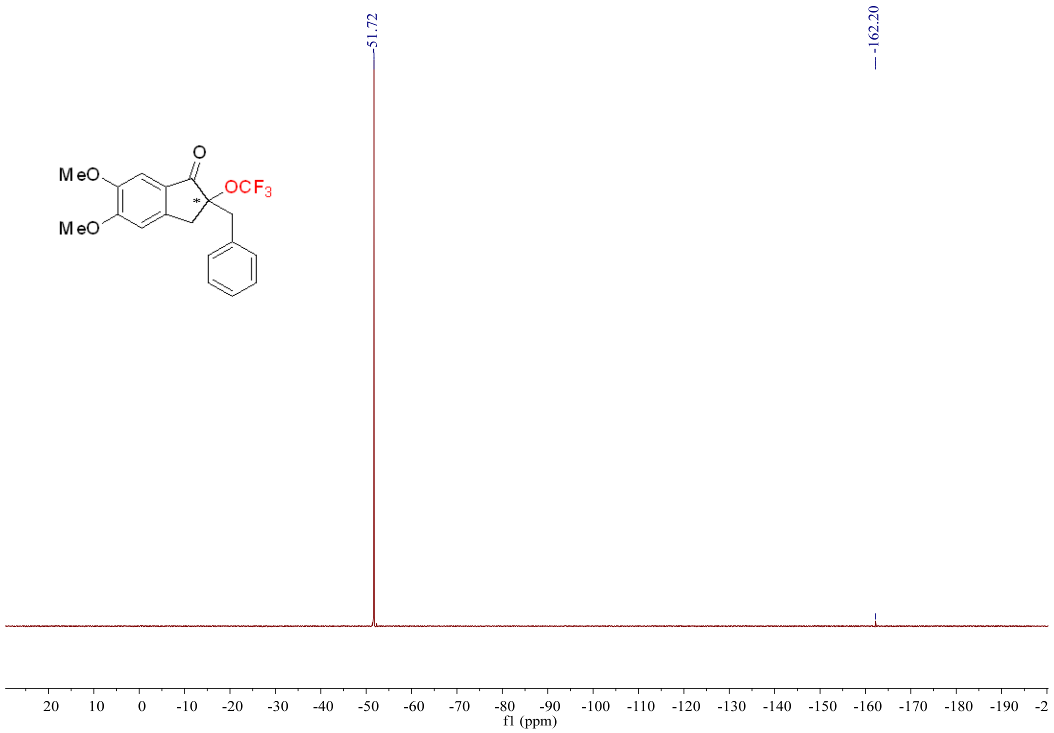

*2-Benzyl-2-(trifluoromethoxy)-3,4-dihydronaphthalen-1(2H)-one (**3ha**).*

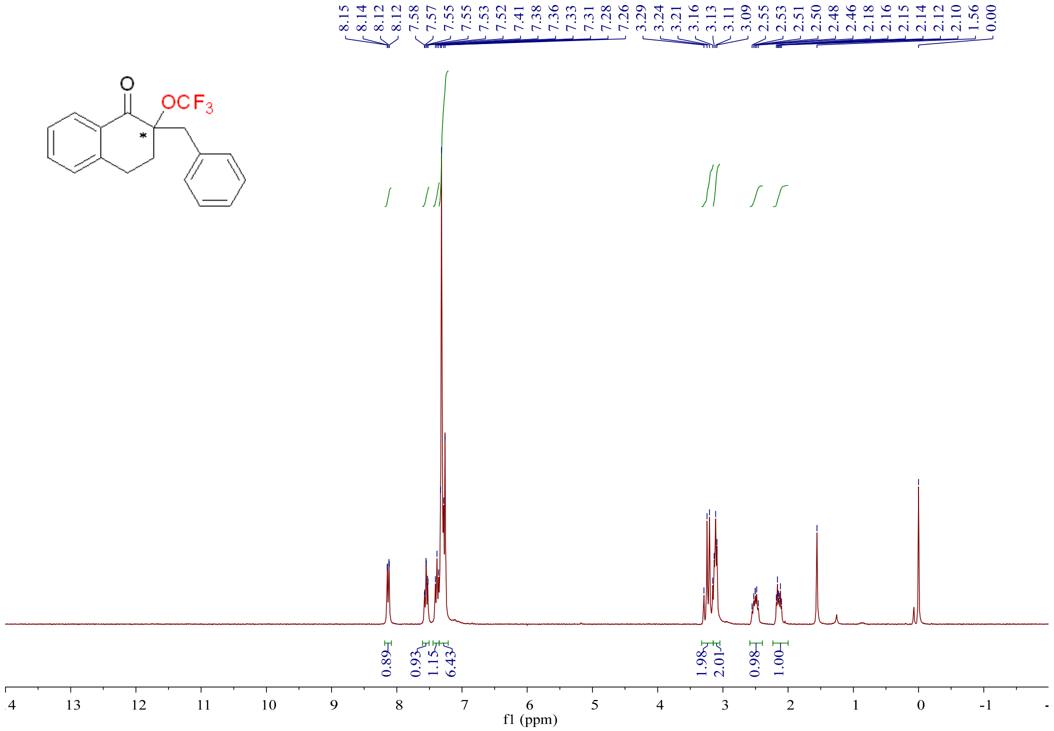

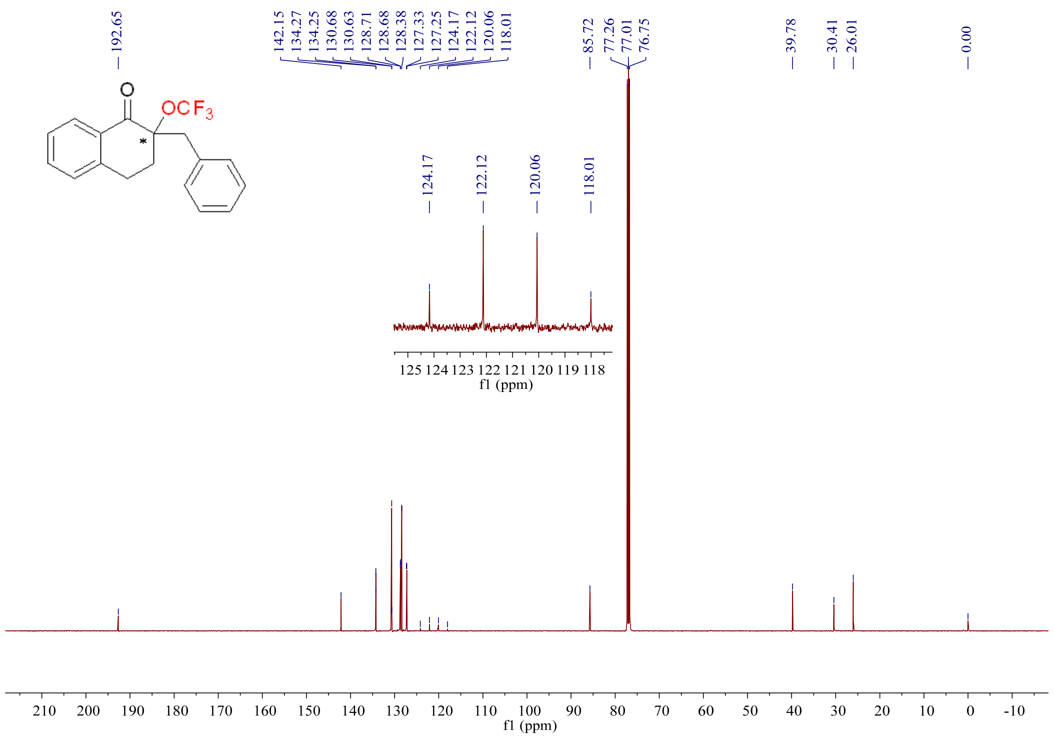

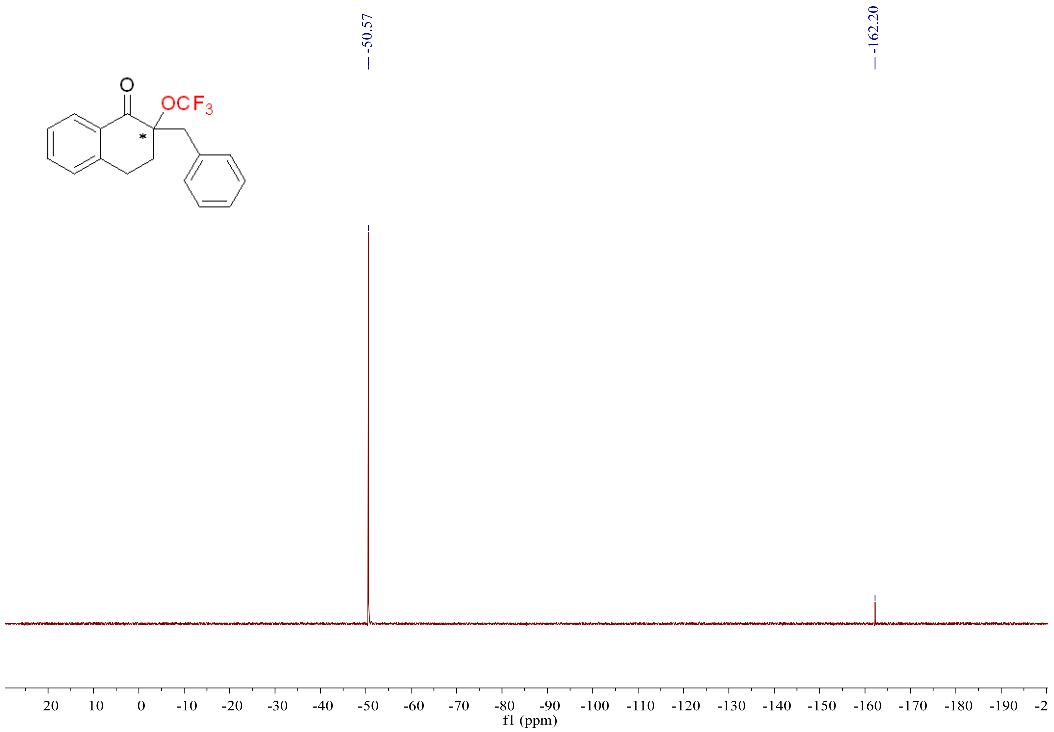

*(R)-2-allyl-2-(trifluoromethoxy)-2,3-dihydro-1H-inden-1-one (**3ai**).*

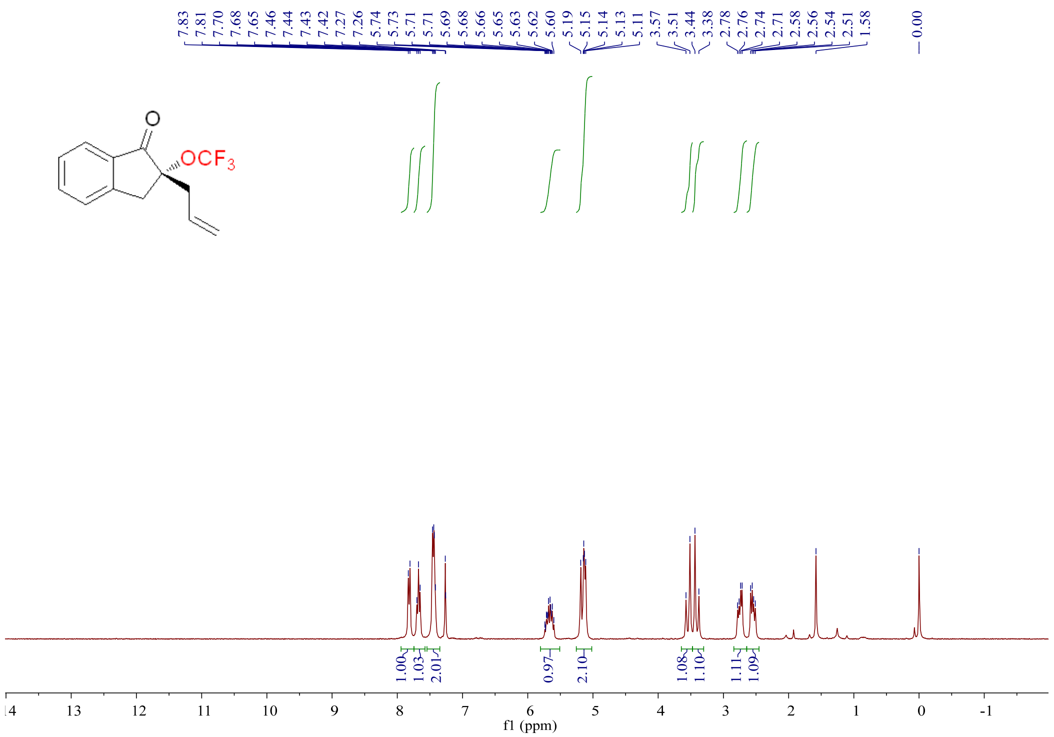

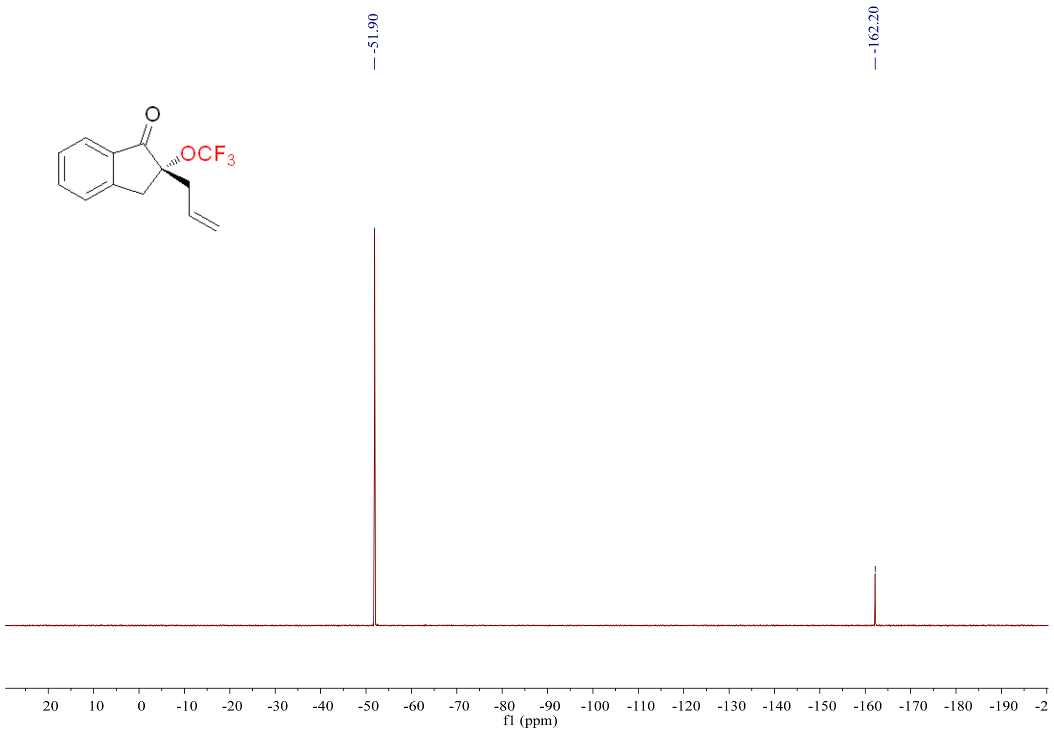

*(R)-2-allyl-5-fluoro-2-(trifluoromethoxy)-2,3-dihydro-1H-inden-1-one (**3ci**)*

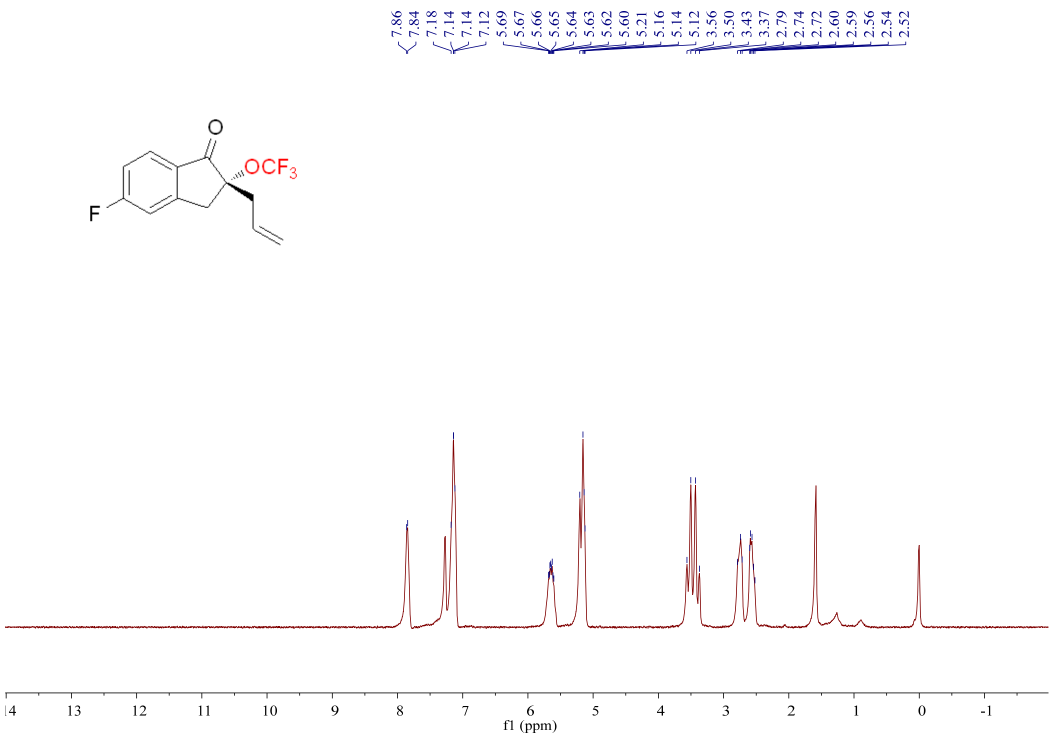

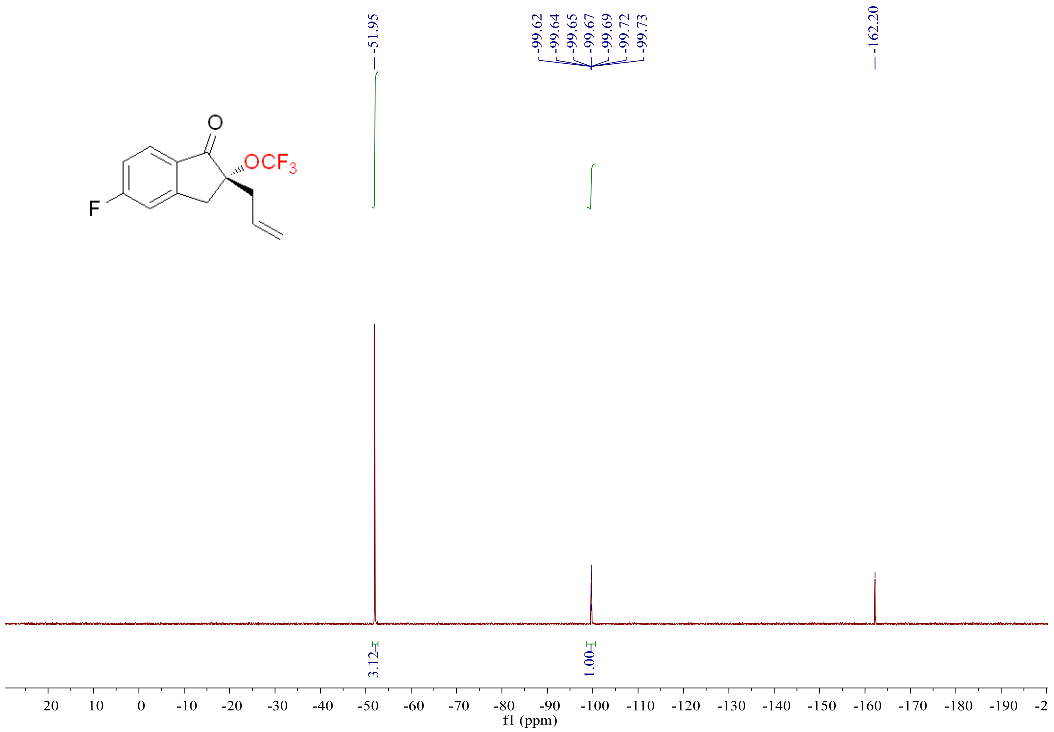

*(R)-2-allyl-6-methyl-2-(trifluoromethoxy)-2,3-dihydro-1H-inden-1-one (**3ei**)*

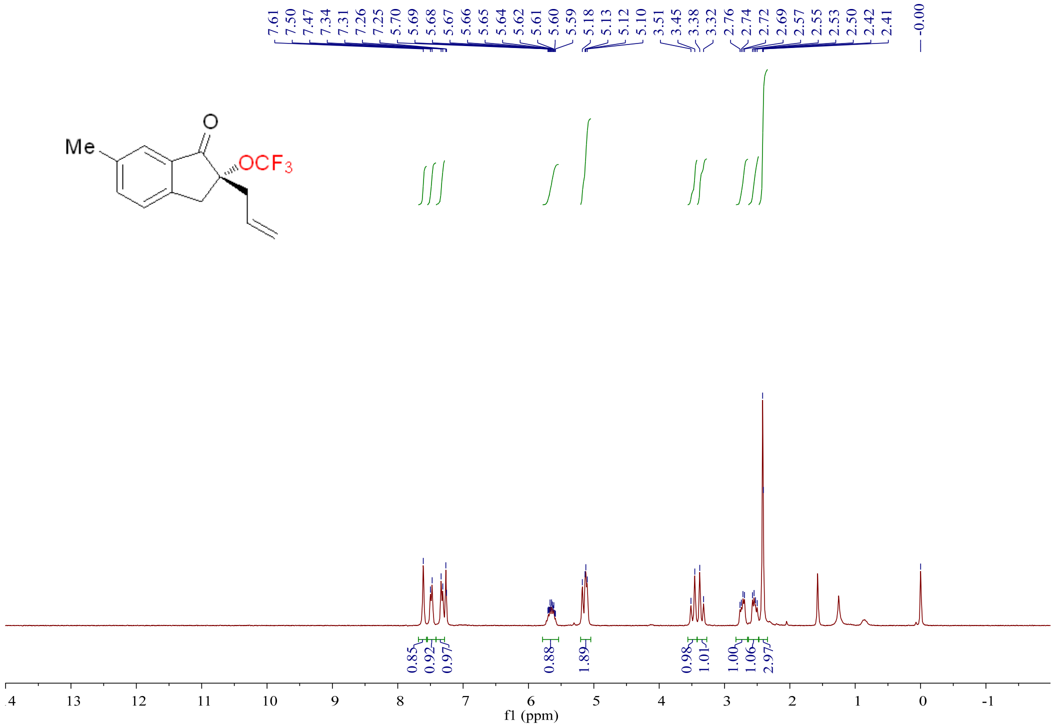
7.61
7.50
7.47
7.34
7.31
7.26
7.25
5.70
5.69
5.68
5.67
5.66
5.65
5.64
5.62
5.61
5.60
5.59
5.18
5.13
5.12
5.10
3.51
3.45
3.38
3.32
2.76
2.74
2.72
2.69
2.57
2.55
2.53
2.50
2.42
2.41
-0.00
Me
O
OCF3
0.85
0.92
0.97
0.88
1.89
0.98
1.01
1.00
1.06
2.97
f1 (ppm)

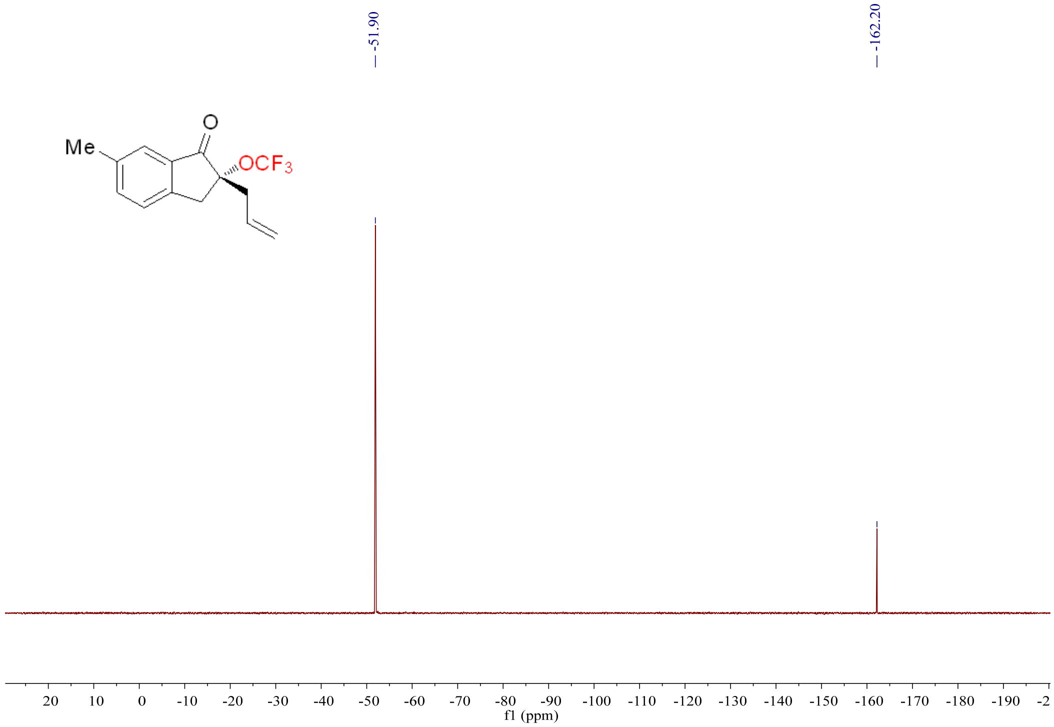
-51.90
-162.20
Me
O
OCF3
f1 (ppm)

3. HPLC data for desired compounds (+)-3.

*(+)-2-Benzyl-2-(trifluoromethoxy)-2,3-dihydro-1H-inden-1-one ((+)-**3aa**).*

CHIRALCEL® OJ-H column (*n*-hexane/isopropanol = 99.0/1.0, flow rate 1.0 mL/min, λ = 254 nm)

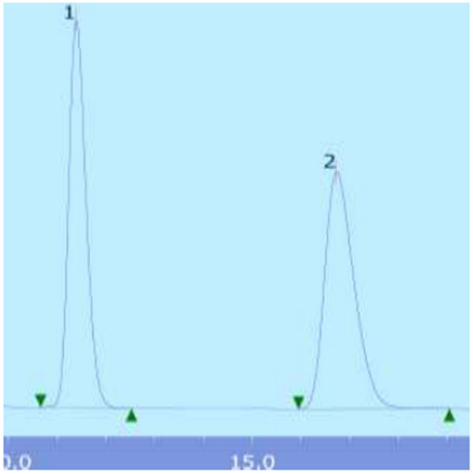

No.	tR (min)	Area (%)	Height (%)
1	11.383	49.187	62.021
2	16.725	50.813	37.979

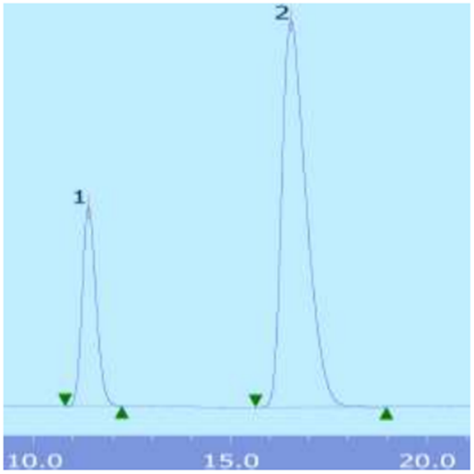

No.	tR (min)	Area (%)	Height (%)
1	11.367	22.951	34.028
2	16.525	77.049	65.972

*(+)-2-(4-Fluorobenzyl)-2-(trifluoromethoxy)-2,3-dihydro-1H-inden-1-one ((+)-**3ab**).*

CHIRALCEL® OJ-H column (*n*-hexane/isopropanol = 99.0/1.0, flow rate 1.0 mL/min, λ = 254 nm)

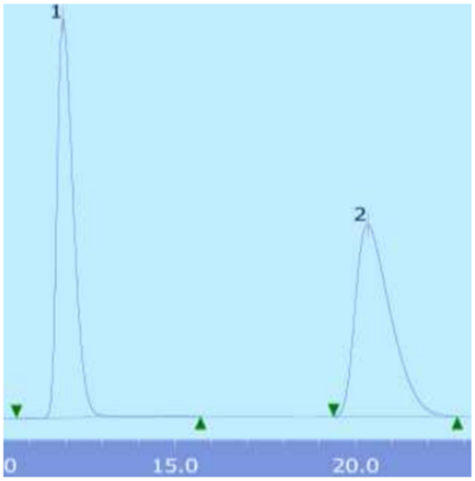

No.	tR (min)	Area (%)	Height (%)
1	11.917	49.041	67.456
2	20.325	50.959	32.544

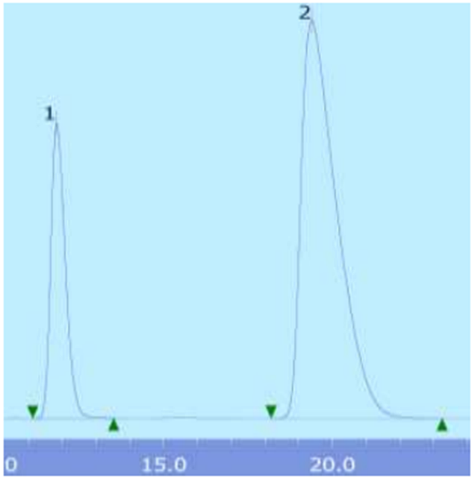

No.	tR (min)	Area (%)	Height (%)
1	11.833	23.329	42.583
2	19.383	76.671	57.417

*(+)-2-(4-Bromobenzyl)-2-(trifluoromethoxy)-2,3-dihydro-1H-inden-1-one ((+)-**3ac**).*

CHIRALCEL® OJ-H column (*n*-hexane/isopropanol = 99.0/1.0, flow rate 1.0 mL/min, λ = 254 nm)

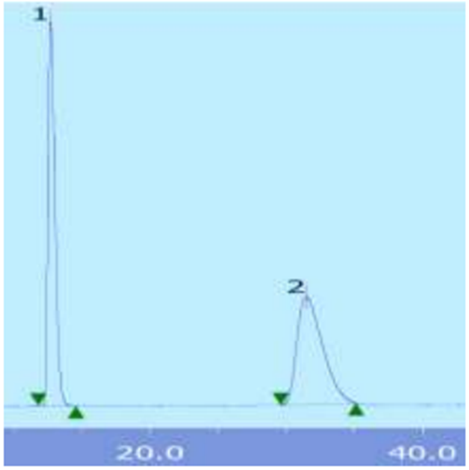

No.	tR (min)	Area (%)	Height (%)
1	12.717	49.308	78.060
2	31.458	50.692	21.940

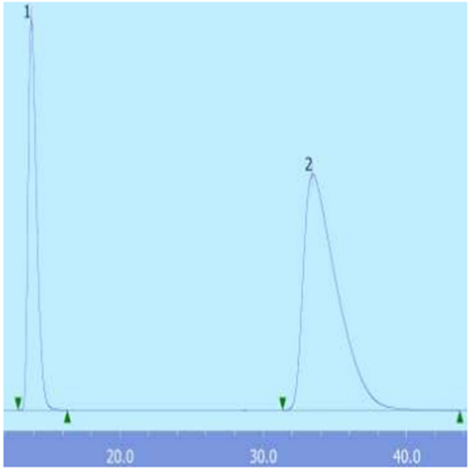

No.	tR (min)	Area (%)	Height (%)
1	13.825	29.570	62.319
2	33.433	70.430	37.681

*(+)-2-(Trifluoromethoxy)-2-(4-(trifluoromethyl)benzyl)-2,3-dihydro-1H-inden-1-one ((+)-**3ad**).*

CHIRALCEL® OJ-H column (*n*-hexane/isopropanol = 99.0/1.0, flow rate 1.0 mL/min, λ = 254 nm)

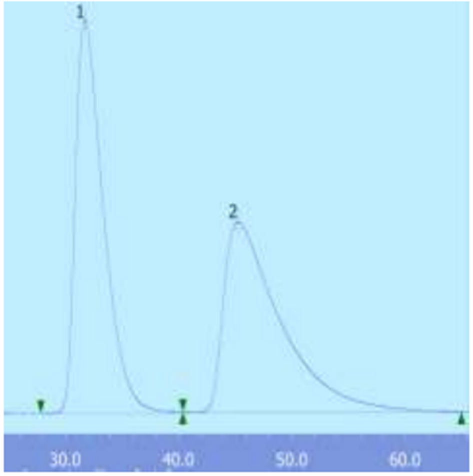

No.	tR (min)	Area (%)	Height (%)
1	31.750	50.264	67.301
2	45.242	49.736	32.699

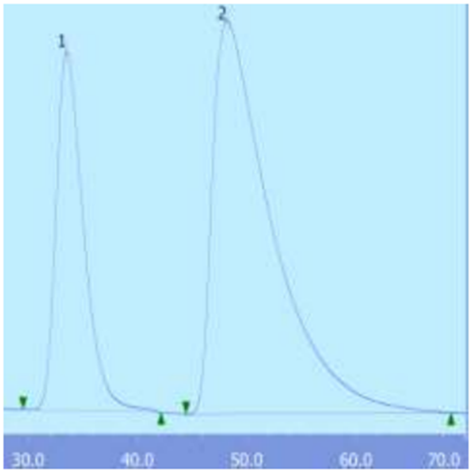

No.	tR (min)	Area (%)	Height (%)
1	33.525	29.766	47.771
2	48.208	70.234	52.229

(+)-2-(3-Fluorobenzyl)-2-(trifluoromethoxy)-2,3-dihydro-1H-inden-1-one ((+)-**3ae**).

CHIRALCEL® OJ-H column (*n*-hexane/isopropanol = 99.0/1.0, flow rate 1.0 mL/min, λ = 254 nm)

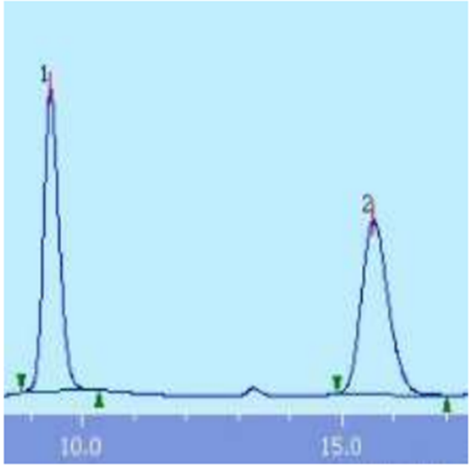

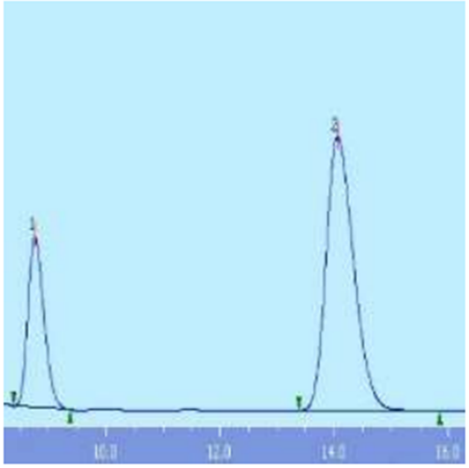

No.	tR (min)	Area (%)	Height (%)
1	9.383	50.207	63.207
2	15.592	49.793	36.529

No.	tR (min)	Area (%)	Height (%)
1	8.758	26.676	38.519
2	14.050	73.324	61.481

*(+)-2-([1,1'-Biphenyl]-4-ylmethyl)-2-(trifluoromethoxy)-2,3-dihydro-1H-inden-1-one ((+)-**3af**)*.
CHIRALPAK ID® column (*n*-hexane/isopropanol = 99.0/1.0, flow rate 1.0 mL/min, λ = 254 nm)

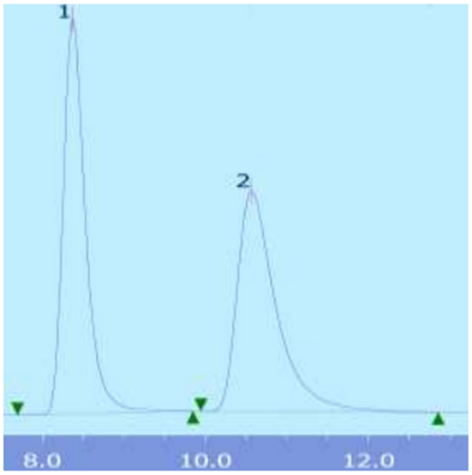

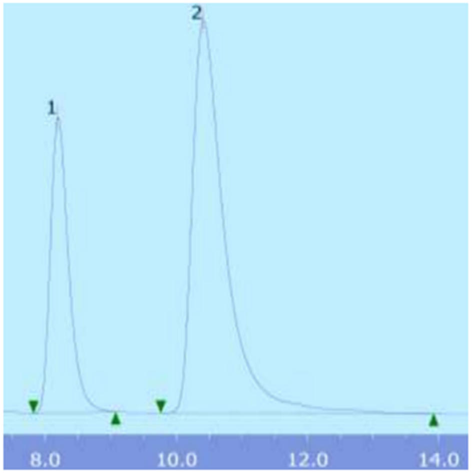

No.	tR (min)	Area (%)	Height (%)
1	8.367	50.640	64.122
2	10.567	49.360	35.878

No.	tR (min)	Area (%)	Height (%)
1	8.192	29.627	42.966
2	10.408	70.373	57.034

*(+)-2-(Naphthalen-2-ylmethyl)-2-(trifluoromethoxy)-2,3-dihydro-1H-inden-1-one ((+)-**3ag**)*.
CHIRALPAK® ID (*n*-hexane/isopropanol = 99.5/0.5, flow rate 1.0 mL/min, λ = 254 nm)

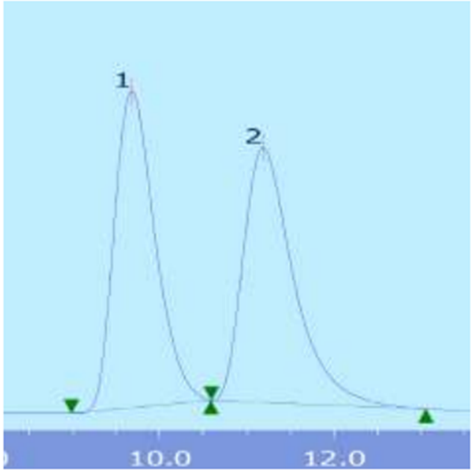

No.	tR (min)	Area (%)	Height (%)
1	9.675	50.150	55.367
2	11.183	49.850	44.633

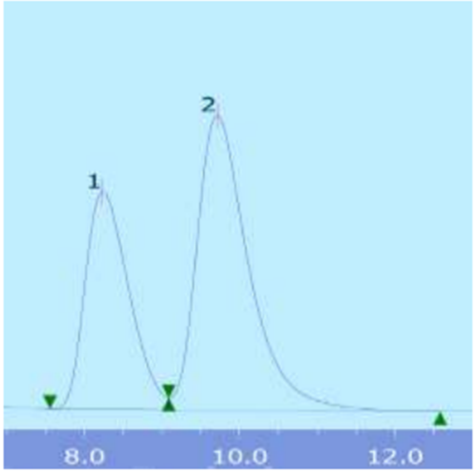

No.	tR (min)	Area (%)	Height (%)
1	8.233	38.138	42.463
2	9.708	61.862	57.537

*(+)-2-(3,5-di-tert-butylbenzyl)-2-(trifluoromethoxy)-2,3-dihydro-1H-inden-1-one ((+)-**3ah**)*.

CHIRALPAK® IF column (*n*-hexane/TBME = 95.0/5.0, flow rate 0.5 mL/min, λ = 254 nm)

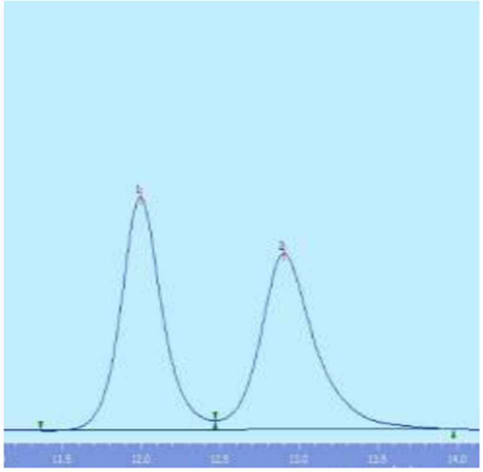

No.	tR (min)	Area (%)	Height (%)
1	11.992	49.919	56.937
2	12.900	50.081	43.063

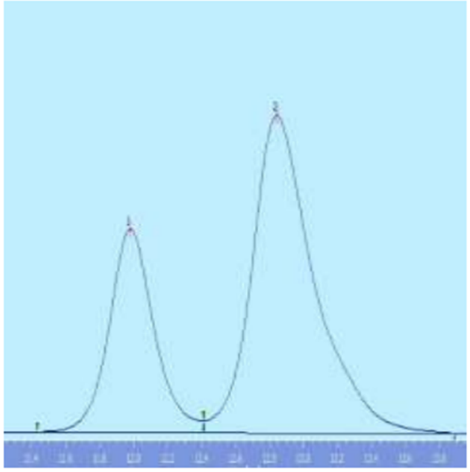

No.	tR (min)	Area (%)	Height (%)
1	11.975	31.740	39.064
2	12.842	68.260	60.936

*(+)-2-Benzyl-5-bromo-2-(trifluoromethoxy)-2,3-dihydro-1H-inden-1-one ((+)-**3ba**)*.

CHIRALCEL® OJ-H column (*n*-hexane/isopropanol = 99.0/1.0, flow rate 1.0 mL/min, λ = 254 nm)

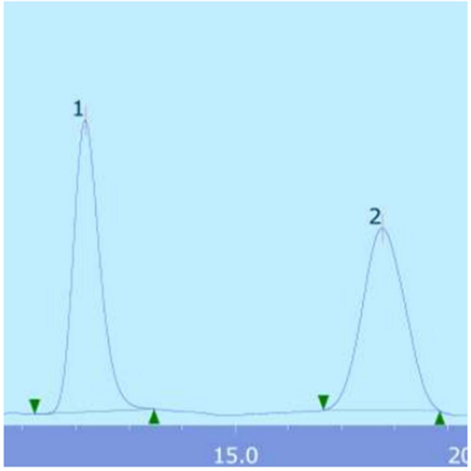

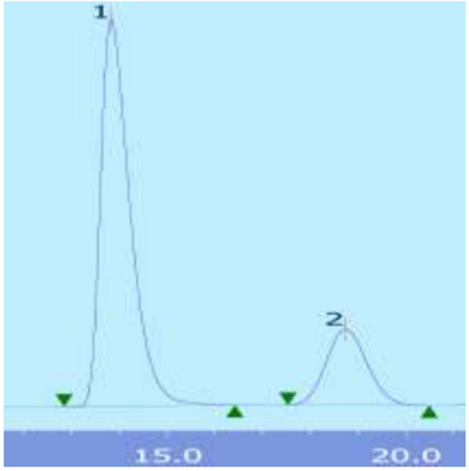

No.	tR (min)	Area (%)	Height (%)
1	12.175	49.783	61.516
2	17.758	50.217	38.484

No.	tR (min)	Area (%)	Height (%)
1	13.833	78.513	83.559
2	18.725	21.487	16.441

*(+)-2-Benzyl-5-fluoro-2-(trifluoromethoxy)-2,3-dihydro-1H-inden-1-one ((+)-**3ca**)*.

CHIRALCEL® OJ-H column (*n*-hexane/isopropanol = 99.0/1.0, flow rate 1.0 mL/min, λ = 254 nm)

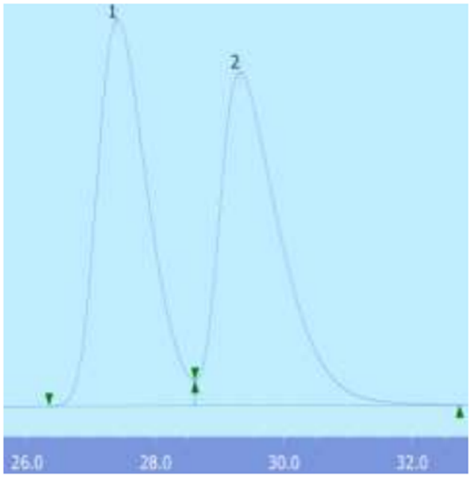

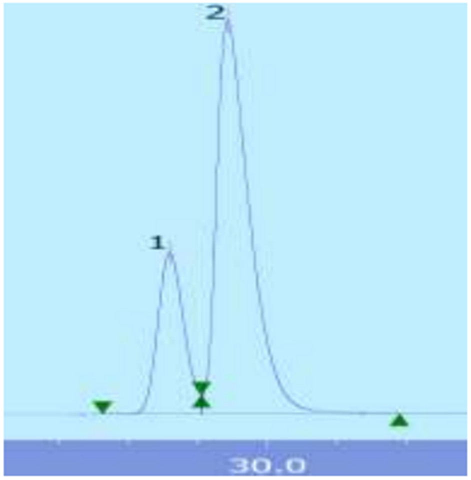

No.	tR (min)	Area (%)	Height (%)
1	27.400	49.349	53.689
2	29.317	50.651	46.311

No.	tR (min)	Area (%)	Height (%)
1	27.167	24.458	29.102
2	28.833	75.542	70.898

*(+)-2-Benzyl-6-fluoro-2-(trifluoromethoxy)-2,3-dihydro-1H-inden-1-one ((+)-**3da**)*.

CHIRALCEL® OJ-H column (*n*-hexane/isopropanol = 99.0/1.0, flow rate 1.0 mL/min, λ = 254 nm)

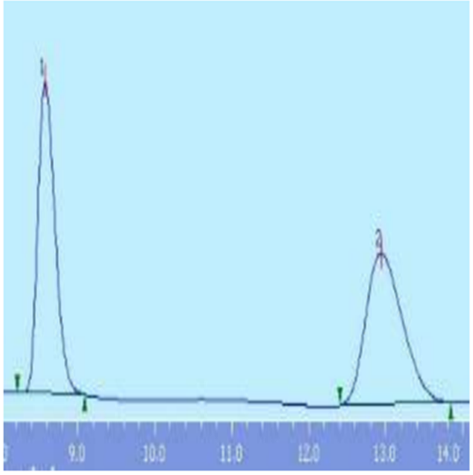

No.	tR (min)	Area (%)	Height (%)
1	8.575	49.214	67.417
2	12.925	50786	32.583

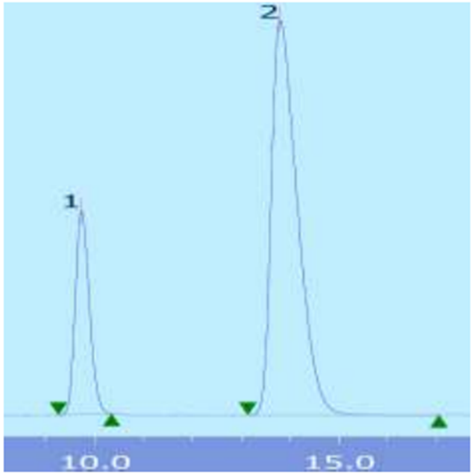

No.	tR (min)	Area (%)	Height (%)
1	9.725	21.894	33.692
2	13.792	78.106	66.038

*(+)-2-Benzyl-6-methyl-2-(trifluoromethoxy)-2,3-dihydro-1H-inden-1-one ((+)-**3ea**)*.

CHIRALCEL® OJ-H column (*n*-hexane/isopropanol = 99.0/1.0, flow rate 1.0 mL/min, λ = 254 nm)

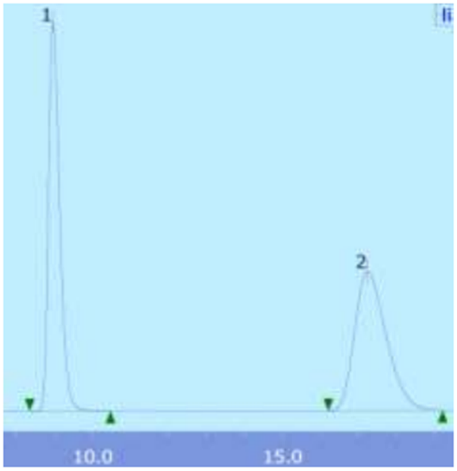

No.	tR (min)	Area (%)	Height (%)
1	8.950	49.592	73.600
2	17.233	50.408	26.400

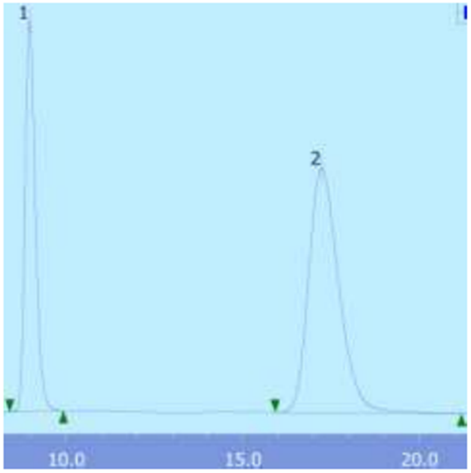

No.	tR (min)	Area (%)	Height (%)
1	8.958	35.360	61.540
2	17.217	64.640	38.460

*(+)-2-Benzyl-6-methoxy-2-(trifluoromethoxy)-2,3-dihydro-1H-inden-1-one ((+)-**3fa**)*.

CHIRALCEL® OJ-H column (*n*-hexane/isopropanol = 99.0/1.0, flow rate 1.0 mL/min, λ = 254 nm)

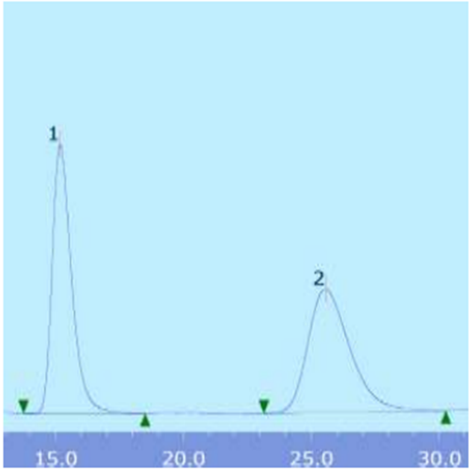

No.	tR (min)	Area (%)	Height (%)
1	15.167	50.070	68.488
2	25.533	49.930	31.512

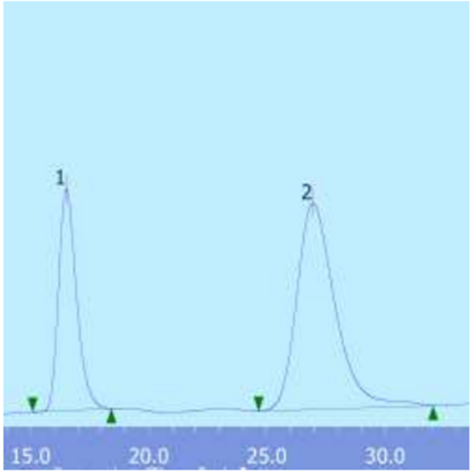

No.	tR (min)	Area (%)	Height (%)
1	16.517	33.379	51.837
2	26.942	66.621	48.163

*(+)-2-Benzyl-5,6-dimethoxy-2-(trifluoromethoxy)-2,3-dihydro-1H-inden-1-one ((+)-**3ga**)*.

A series of CHIRALPAK® IF and CHIRALPAK® IA-3 (*n*-hexane/isopropanol = 90.0/10.0, flow rate 1.0 mL/min, λ = 254 nm)

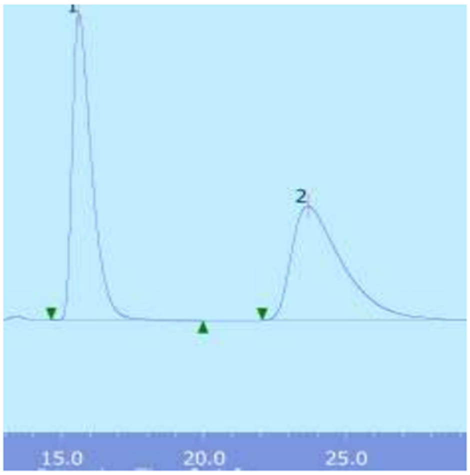

No.	tR (min)	Area (%)	Height (%)
1	15.608	50.240	72.807
2	23.642	49.760	27.193

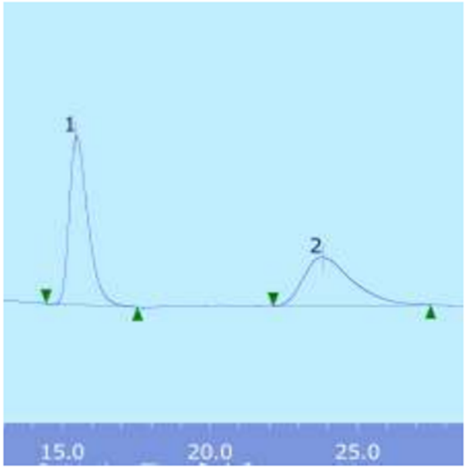

No.	tR (min)	Area (%)	Height (%)
1	15.475	56.434	77.595
2	23.808	43.566	22.405

*(+)-2-Benzyl-2-(trifluoromethoxy)-3,4-dihydronaphthalen-1(2H)-one ((+)-**3ha**)*.

CHIRALCEL® OJ-H column (*n*-hexane/isopropanol = 99.0/1.0, flow rate 1.0 mL/min, λ = 254 nm)

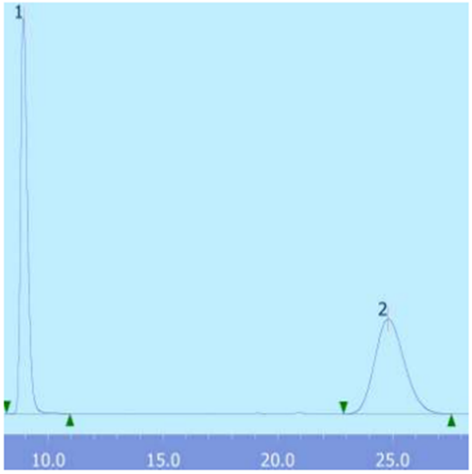

No.	tR (min)	Area (%)	Height (%)
1	8.942	49.269	80.492
2	24.800	50.731	19.508

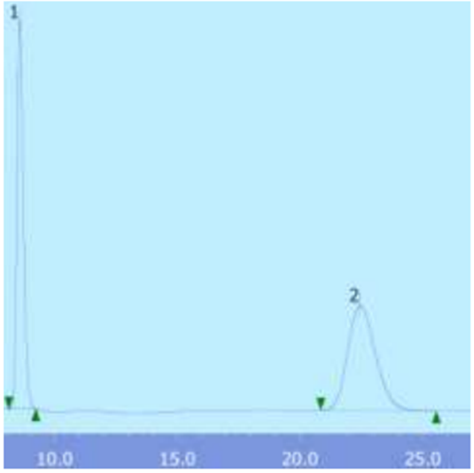

No.	tR (min)	Area (%)	Height (%)
1	8.583	46.178	78.657
2	22.442	53.822	21.343

4. HPLC data for desired compounds (−)-3 in Scheme 3.

*(−)-2-Benzyl-2-(trifluoromethoxy)-2,3-dihydro-1H-inden-1-one ((−)-**3aa**).*

CHIRALCEL® OJ-H column (*n*-hexane/isopropanol = 99.0/1.0, flow rate 1.0 mL/min, λ = 254 nm)

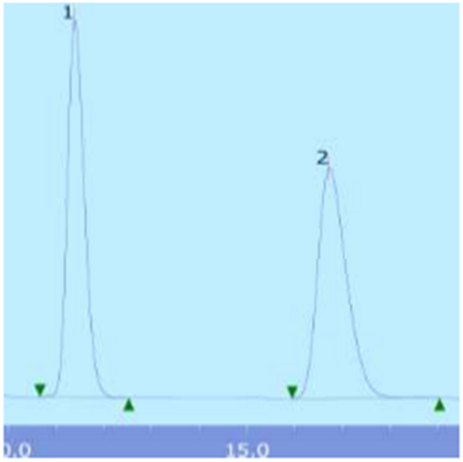

No.	tR (min)	Area (%)	Height (%)
1	11.383	49.187	62.021
2	16.725	50.813	37.979

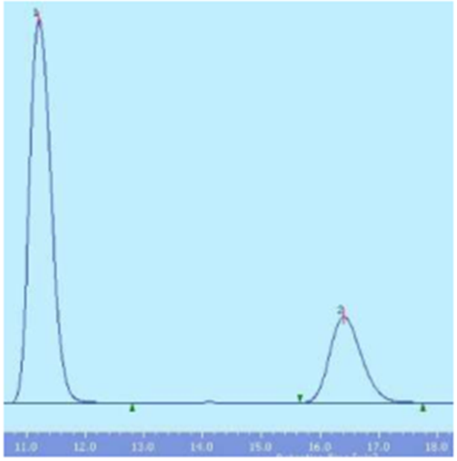

No.	tR (min)	Area (%)	Height (%)
1	11.208	75.018	81.676
2	16.400	24.982	18.324

*(−)-2-(4-Fluorobenzyl)-2-(trifluoromethoxy)-2,3-dihydro-1H-inden-1-one ((−)-**3ab**).*

CHIRALCEL® OJ-H column (*n*-hexane/isopropanol = 99.0/1.0, flow rate 1.0 mL/min, λ = 254 nm)

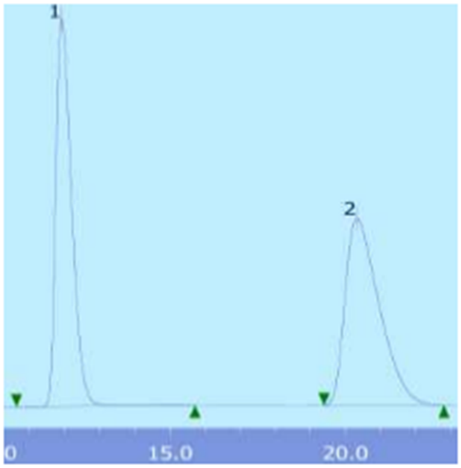

No.	tR (min)	Area (%)	Height (%)
1	11.917	49.041	67.456
2	20.325	50.959	32.544

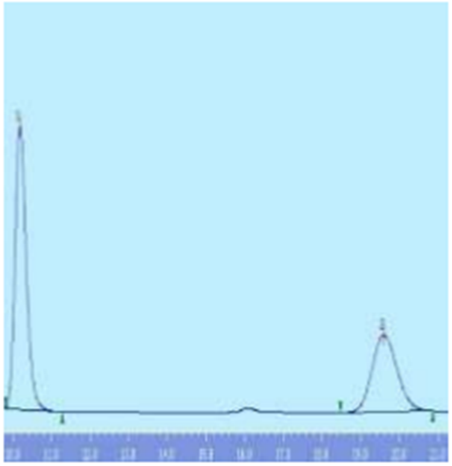

No.	tR (min)	Area (%)	Height (%)
1	11.833	62.062	78.708
2	19.383	37.938	21.292

*(−)-2-(4-Bromobenzyl)-2-(trifluoromethoxy)-2,3-dihydro-1H-inden-1-one ((−)-**3ac**)*.

CHIRALCEL® OJ-H column (*n*-hexane/isopropanol = 99.0/1.0, flow rate 1.0 mL/min, λ = 254 nm)

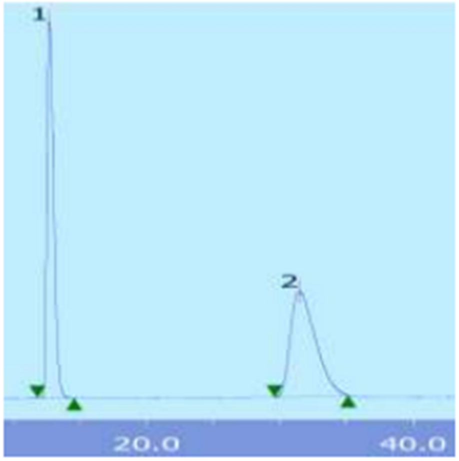

No.	tR (min)	Area (%)	Height (%)
1	12.717	49.308	78.060
2	31.458	50.692	21.940

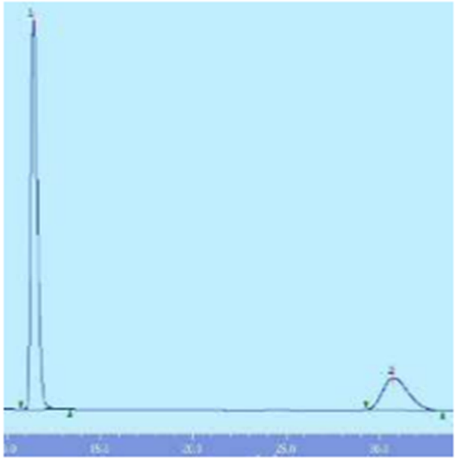

No.	tR (min)	Area (%)	Height (%)
1	11.400	75.156	92.305
2	30.725	24.844	7.695

(−)-2-(3-Fluorobenzyl)-2-(trifluoromethoxy)-2,3-dihydro-1H-inden-1-one (*(−)*-**3ae**).

CHIRALCEL® OJ-H column (*n*-hexane/isopropanol = 99.0/1.0, flow rate 1.0 mL/min, λ = 254 nm)

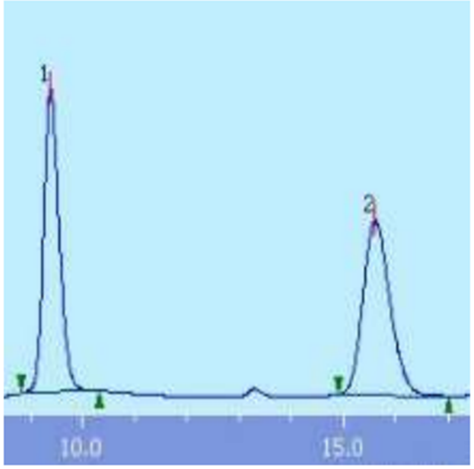

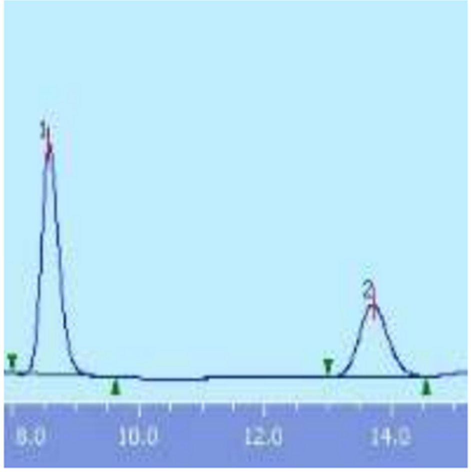

No.	tR (min)	Area (%)	Height (%)
1	9.383	50.207	63.207
2	15.592	49.793	36.529

No.	tR (min)	Area (%)	Height (%)
1	8.575	65.866	75.969
2	13.708	34.134	24.031

*(−)-2-([1,1'-Biphenyl]-4-ylmethyl)-2-(trifluoromethoxy)-2,3-dihydro-1H-inden-1-one ((−)-**3af**)*. CHIRALPAK ID® column (*n*-hexane/isopropanol = 99.0/1.0, flow rate 1.0 mL/min, λ = 254 nm)

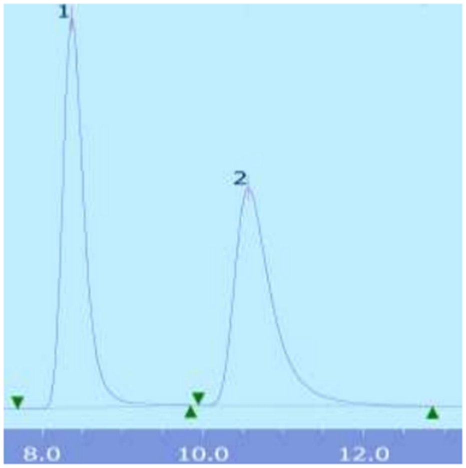

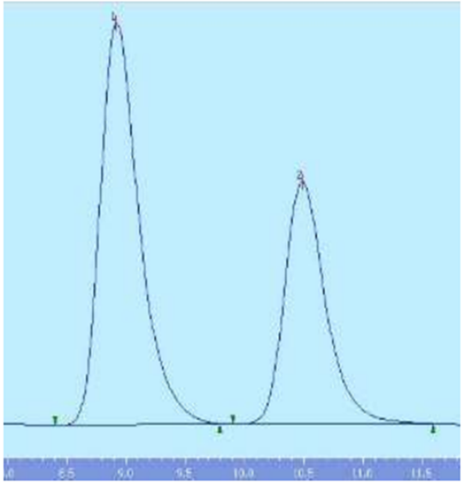

No.	tR (min)	Area (%)	Height (%)
1	8.367	50.640	64.122
2	10.567	49.360	35.878

No.	tR (min)	Area (%)	Height (%)
1	8.917	61.309	62.328
2	10.483	38.691	37.672

*(−)-2-(Naphthalen-2-ylmethyl)-2-(trifluoromethoxy)-2,3-dihydro-1H-inden-1-one ((−)-**3ag**)*. CHIRALPAK® ID (*n*-hexane/isopropanol = 99.5/0.5, flow rate 1.0 mL/min, λ = 254 nm)

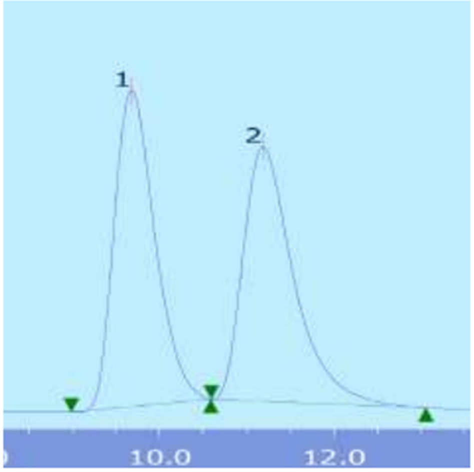

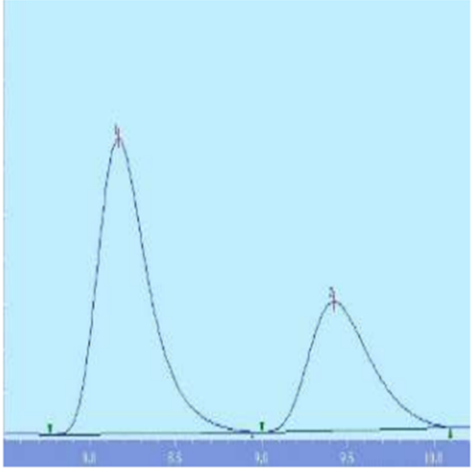

No.	tR (min)	Area (%)	Height (%)
1	9.675	50.150	55.367
2	11.183	49.850	44.633

No.	tR (min)	Area (%)	Height (%)
1	8.167	65.542	69.616
2	9.417	34.458	30.384

*(−)-2-Benzyl-6-fluoro-2-(trifluoromethoxy)-2,3-dihydro-1H-inden-1-one ((−)-**3da**).*

CHIRALCEL® OJ-H column (*n*-hexane/isopropanol = 99.0/1.0, flow rate 1.0 mL/min, λ = 254 nm)

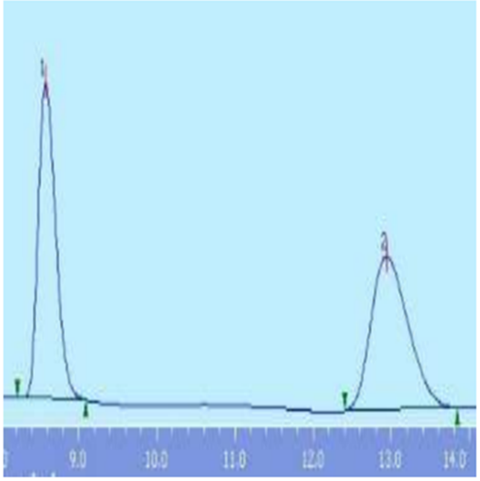

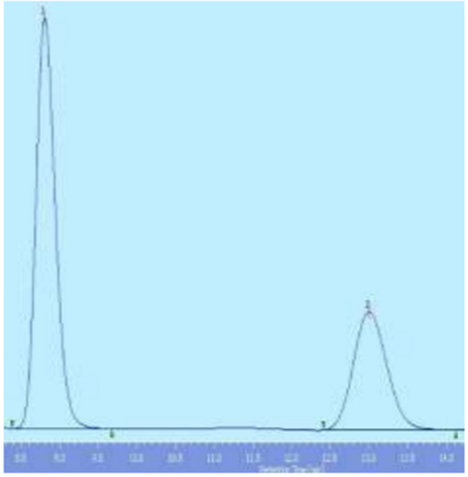

No.	tR (min)	Area (%)	Height (%)
1	8.575	49.214	67.417
2	12.925	50.786	32.583

No.	tR (min)	Area (%)	Height (%)
1	8.800	66.836	77.697
2	12.992	33.164	22.303

5. HPLC data for desired compounds 3 in Scheme 4.

*(R)-2-allyl-2-(trifluoromethoxy)-2,3-dihydro-1H-inden-1-one ((R)-**3ai**).*

CHIRALCEL® OJ-H column (n-hexane/isopropanol = 98.0/2.0, flow rate 0.5 mL/min, λ = 254 nm)

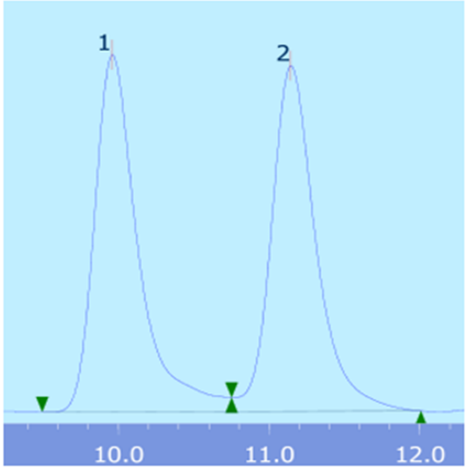

No.	tR (min)	Area (%)	Height (%)
1	9.958	50.392	50.795
2	11.142	49.608	49.205

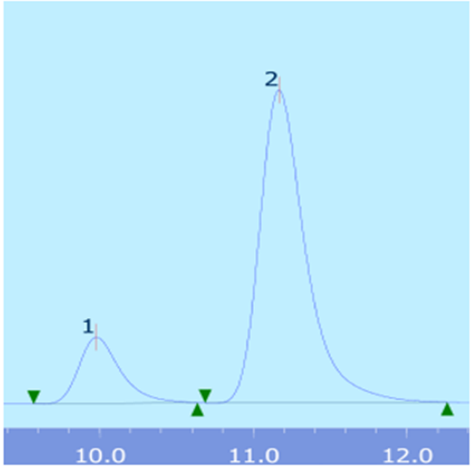

No.	tR (min)	Area (%)	Height (%)
1	9.975	16.072	17.500
2	11.167	83.928	82.500

*(R)-2-allyl-5-fluoro-2-(trifluoromethoxy)-2,3-dihydro-1H-inden-1-one ((R)-**3ci**).*

CHIRALPAK® IF column (n-hexane/TBME = 90.0/10.0, flow rate 0.5 mL/min, λ = 254 nm)

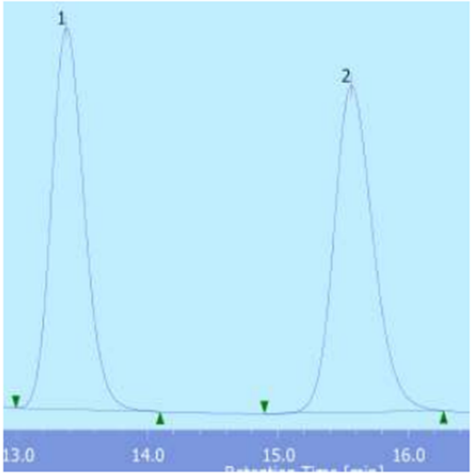

No.	tR (min)	Area (%)	Height (%)
1	13.375	49.408	53.884
2	15.558	50.592	46.116

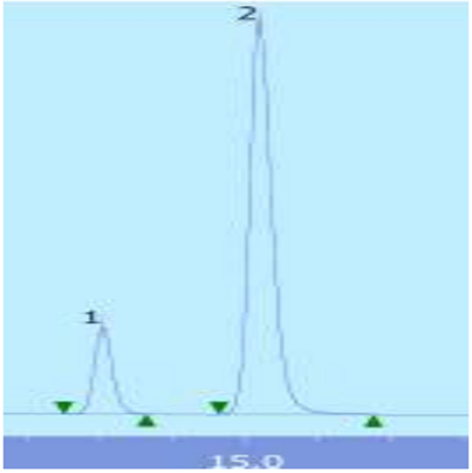

No.	tR (min)	Area (%)	Height (%)
1	13.042	14.916	18.121
2	15.192	85.084	81.879

*(R)-2-allyl-6-methyl-2-(trifluoromethoxy)-2,3-dihydro-1H-inden-1-one ((R)-**3ei**).*

CHIRALPAK® IF column (n-hexane/TBME = 90.0/10.0, flow rate 0.5 mL/min, λ = 254 nm)

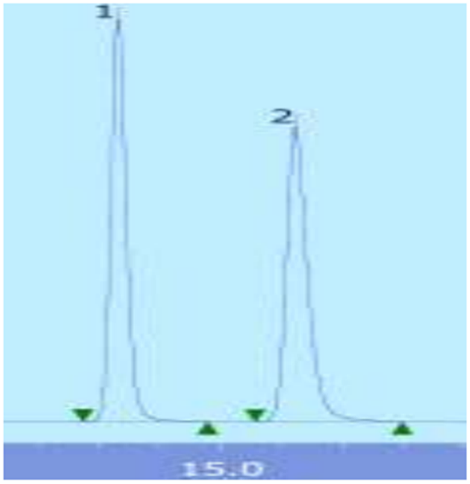

No.	tR (min)	Area (%)	Height (%)
1	13.317	49.678	57.681
2	16.217	50.322	42.319

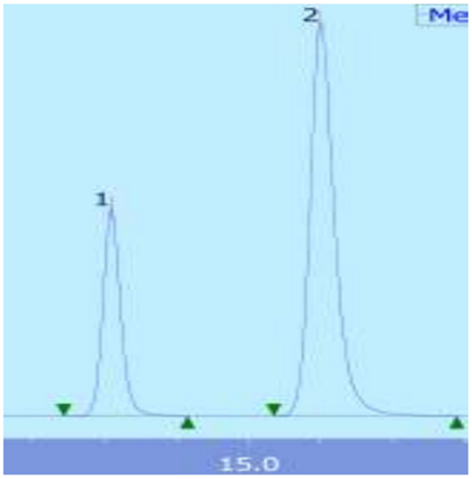

No.	tR (min)	Area (%)	Height (%)
1	13.083	27.491	34.493
2	15.992	72.509	65.507

(S)-2-allyl-2-(trifluoromethoxy)-2,3-dihydro-1H-inden-1-one ***(S-3ai)***.

CHIRALCEL® OJ-H column (*n*-hexane/isopropanol = 98.0/2.0, flow rate 0.5 mL/min, λ = 254 nm)

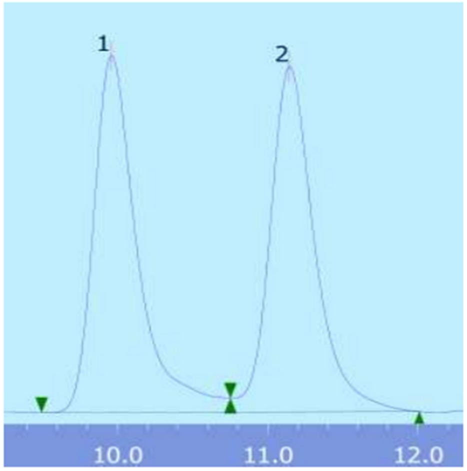

No.	tR (min)	Area (%)	Height (%)
1	9.958	50.392	50.795
2	11.142	49.608	49.205

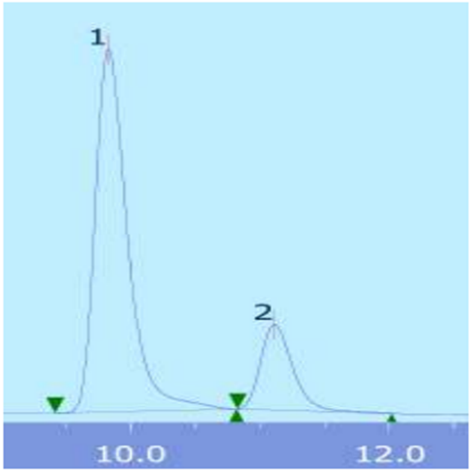

No.	tR (min)	Area (%)	Height (%)
1	9.833	81.138	80.907
2	11.108	18.862	19.093

(S)-2-allyl-5-fluoro-2-(trifluoromethoxy)-2,3-dihydro-1H-inden-1-one ***(S-3ci)***.

CHIRALPAK® IF column (*n*-hexane/TBME = 90.0/10.0, flow rate 0.5 mL/min, λ = 254 nm)

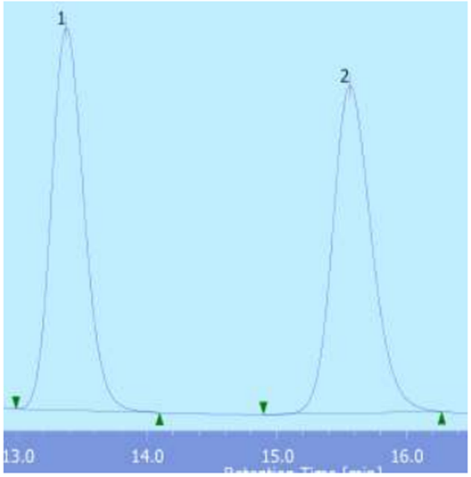

No.	tR (min)	Area (%)	Height (%)
1	13.375	49.408	53.884
2	15.558	50.592	46.116

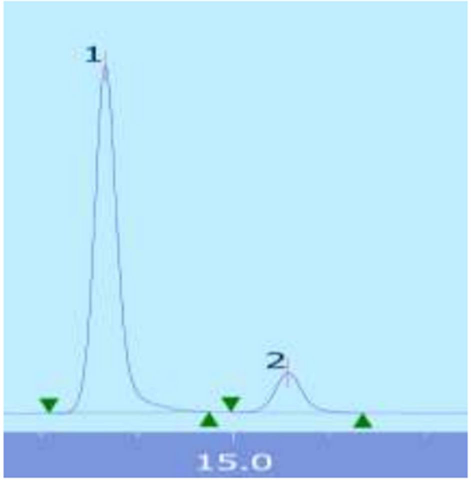

No.	tR (min)	Area (%)	Height (%)
1	13.658	88.146	89.726
2	15.567	11.854	10.274

*(S)-2-allyl-6-methyl-2-(trifluoromethoxy)-2,3-dihydro-1H-inden-1-one (**S-3ei**).*

CHIRALPAK® IF column (*n*-hexane/TBME = 90.0/10.0, flow rate 0.5 mL/min, λ = 254 nm)

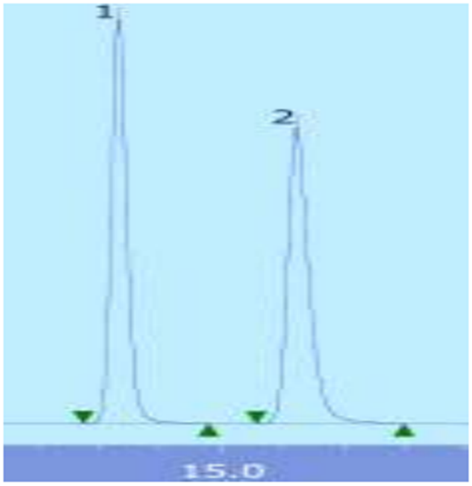

No.	tR (min)	Area (%)	Height (%)
1	13.317	49.678	57.681
2	16.217	50.322	42.319

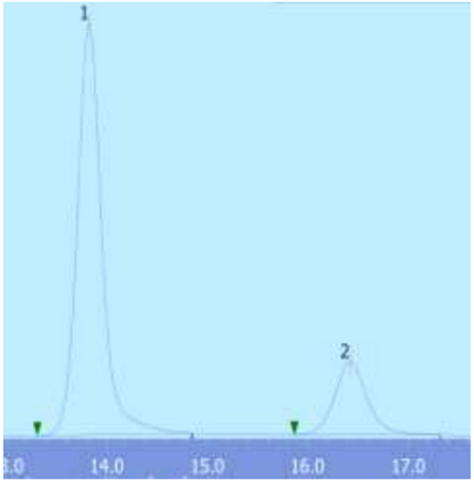

No.	tR (min)	Area (%)	Height (%)
1	13.825	81.067	85.009
2	16.425	18.933	14.991
